# Traduzione e adattamento alla lingua italiana del glossario dei termini più comunemente usati dagli elettroencefalografisti clinici e proposta per il formato del referto EEG (Revisione IFCN 2017)

**DOI:** 10.1016/j.cnp.2022.09.006

**Published:** 2022-11-08

**Authors:** Gionata Strigaro, Francesca Bisulli, Giovanni Assenza, Oriano Mecarelli, Antonello Grippo, Stefano Meletti, ON BEHLAF OF LICE-SINC Glossary Study Group, Lara Alvisi, Gaetano Cantalupo, Roberto Eleopra, Angelo Guerra, Silvia Lori, Lucio Marinelli, Laura Tassi, Paolo Tinuper, Federico Vigevano

**Affiliations:** a Neurology Unit, Department of Translational Medicine, University of Piemonte Orientale, and Azienda Ospedaliero-Universitaria “Maggiore della Carità”, Novara, Italy; b IRCCS Istituto delle Scienze Neurologiche di Bologna, UOC Clinica Neurologica, Bologna, Italy; c Department of Biomedical and Neuromotor Sciences, University of Bologna, Italy; d Unit of Neurology, Neurophysiology and Neurobiology Department of Medicine and Surgery, Università Campus Bio-Medico di Roma, Via Alvaro del Portillo, 21 - 00128 Roma, Italy; e Department of Human Neurosciences, Sapienza University, Rome and Past President of LICE, Italian League Against Epilepsy, Rome, Italy; f Neurophysiology Unit, Careggi University Hospital, Florence, Italy; g Neurology Unit, OCB Hospital, Azienda Ospedaliera-Universitaria di Modena, Modena, Italy; h Department of Biomedical, Metabolic and Neural Science, University of Modena and Reggio Emilia, Modena, Italy; a IRCCS Istituto delle Scienze Neurologiche di Bologna, UOC Clinica Neurologica, Bologna, Italy; b Department of Biomedical and Neuromotor Sciences, University of Bologna, Italy; c Neurophysiology Unit, Careggi University Hospital, Florence, Italy; d Centro Ricerca per le Epilessie in età Pediatrica (CREP), Azienda Ospedaliera Universitaria di Verona, Verona, Italy; e Fondazione IRCCS Istituto Neurologico Carlo Besta, Neurology Unit 1, Milan, Italy; f Division of Clinical Neurophysiology, Department of Neuroscience, IRCCS Ospedale Policlinico San Martino, Genoa, Italy; g “Claudio Munari” Epilepsy Surgery Centre, ASST Niguarda Hospital, Milano, Italy; h Division of Neurology, Bambino Gesù Children's Hospital IRCCS, Rome, Italy

## 1 Introduction

The Italian translation of the revised glossary of terms most commonly used by clinical electroencephalographers (Kane et al., 2017) is the result of the effort of a joint task force between a panel of experts from the Committee on “Epilepsy and Neurophysiological Techniques” of the Italian League Against Epilepsy (LICE) and the Italian Clinical Neurophysiology Society (SINC). It comes more than 40 years after the first translation from the former Società Italiana di Elettroencefalografia e Neurofisiologia (1978), based on the pioneering “glossary of terms most commonly used by clinical electroencephalographers” published in 1974 (Chatrian et al., 1974). It is a long-awaited attempt to standardize the clinical description and reporting of EEG across the Italian-speaking territory. It is believed that a shared terminology is the precondition for correct and meaningful interpretation of EEG as testified by the similar attempt of Portuguese colleagues (Gonçalves et al., 2022).

The translation underwent a series of internal revisions that ended to the “Palermo consensus” held during the 66th SINC National Congress in May 2022. Experts in neonatal EEG and EEG technicians were also involved to revise the pertaining terminology. Finally, external referees with established experience in the interpretation of the EEG in children and adults revised the entire translation.

Most of the terms has been thoroughly translated except those that have already come into common use which have been maintained in English, both single words (i.e., bin, brush, burst, mismatch negativity) and acronyms, the full list of which is reported with explanations at the end of the appendix.

The Italian version with facing text of the introduction and glossary of EEG terms as proposed by Kane et al. (2017) is hereby presented.

**Table:** 

This glossary includes the terms most commonly used in clinical EEG. It is based on the previous proposals (Chatrian et al., 1974, Noachtar et al., 1999) and includes terms necessary to describe the EEG and to generate the EEG report. All EEG phenomena should be described as precisely as possible in terms of frequency, amplitude, phase relation, waveform, localization, quantity, and variability of these parameters (Brazier et al., 1961). The description should be independent of the recording parameters such as amplification, montages, and computer program/display. Biological and technical artifacts that interfere with an adequate EEG interpretation should either be eliminated or, if this is not possible, be noted in the description.	Questo glossario comprende i termini più comunemente utilizzati nella refertazione dell'EEG in ambito clinico. Si basa sulle precedenti versioni pubblicate (Chatrian et al., 1974, Noachtar et al., 1999, Noachtar et al., 1999) e comprende i termini necessari per descrivere l'EEG e per redigere un referto EEG. Tutti i reperti EEG devono essere descritti nel modo più preciso possibile in termini di quantità e variabilità di precisi parametri, quali la frequenza, ampiezza, fase, forma d'onda e localizzazione (Brazier et al., 1961). La descrizione deve essere indipendente dai parametri di registrazione, come l'amplificazione, i montaggi e il programma/display del computer. Gli artefatti, sia di origine biologica che tecnica, che interferiscono con una corretta interpretazione dell'EEG, devono essere eliminati oppure, se ciò non fosse possibile, devono essere segnalati nella descrizione.

The EEG report should follow a standard format that includes a factual description and a clinical interpretation of the EEG record. The interpretation of the EEG requires knowledge of the patient’s age, past medical and medication history, their clinical condition during the EEG, particularly level of consciousness/vigilance and ability to co-operate. The EEG interpretation summarizes the results of the EEG and gives a clinical interpretation in light of the diagnosis and the questions posed by the referring physician. The terminology of the EEG interpretation should follow common neurological and clinical practice and use terms understandable to other physicians not specialized in EEG. A proposal for the EEG report form is given in Appendix A.	Il referto dell'EEG deve rispettare un formato standard che includa una descrizione precisa dei reperti e un'interpretazione clinica. L'interpretazione dell'EEG richiede la conoscenza dell'età del paziente, dell'anamnesi, della terapia farmacologica assunta, delle condizioni cliniche durante l'EEG, in particolare del livello di coscienza/vigilanza e della collaborazione. L'interpretazione dell'EEG riassume le caratteristiche salienti del tracciato e fornisce un'interpretazione clinica alla luce della diagnosi e dei quesiti posti dal medico richiedente l'esame. La terminologia dell'interpretazione EEG deve seguire la comune pratica neurologica e clinica e utilizzare termini comprensibili anche a medici non specialisti in elettroencefalografia. In appendice A è proposto un modello schematico di refertazione.

**Glossary**	**Glossario**

**Absence:** A generalized seizure type. Use of term discouraged when describing EEG patterns. Terms suggested, whenever appropriate: spike-and-slow-wave complex, 3 c/s spike-and-slow-wave complex, sharp-and-slow wave complex.	**Assenza:** un tipo di crisi generalizzata. L'uso di questo termine è sconsigliato per descrivere i pattern EEG per i quali è preferibile utilizzare, se appropriata, una delle seguenti (diciture): complesso di punta-onda-lenta, complesso punta-onda-lenta a 3 Hz, complesso onda puntuta-onda lenta.

**Activation procedure:** Any procedure designed to modulate EEG activity, for instance to enhance physiological waveforms or elicit abnormal paroxysmal activity. Examples include: eye closing, hyperventilation, photic stimulation, natural or drug-induced sleep, sensory stimulation (acoustic, somatosensory or pain).	**Prova di attivazione:** qualsiasi procedura finalizzata a modulare l'attività EEG, potenziando per esempio l'attività fisiologica o favorendo la comparsa di un'attività parossistica anormale. Esempi: chiusura degli occhi, iperventilazione, stimolazione luminosa, sonno naturale o farmaco-indotto, stimolazione sensoriale (acustica, tattile o dolorosa).

**Active sleep:** Normal sleep stage in neonates characterized by eye closure, intermittent periods of rapid eye movements, irregular respirations and scant body movements. The EEG shows activité moyenne in term and near term infants, and tracé discontinue (discontinuous pattern) in preterm infants <34 weeks of post menstrual age (PMA); the inter-burst interval depends on the PMA. (See quiet sleep, activité moyenne, tracé discontinue, REM sleep).	**Sonno attivo**: fase fisiologica del sonno nel neonato caratterizzata dalla chiusura degli occhi, periodi intermittenti di movimenti oculari rapidi, respiro irregolare e movimenti del corpo. L'EEG mostra “activité moyenne” (*lett.* attività intermedia; *sin.* attività a frequenze miste) nei neonati a termine e quasi a termine, e tracciato discontinuo (“tracé discontinu”) nei neonati pretermine con <34 settimane di età gestazionale (EG); l'intervallo inter-burst varia in base alla EG. (Vedi sonno quieto, activité moyenne, tracé discontinu, sonno REM).

**Activity**, **EEG:** An EEG wave or sequence of waves of cerebral origin.	**Attività, EEG:** un'onda o una sequenza di onde EEG di origine cerebrale.

**Activité moyenne:** Neonatal EEG pattern of wakefulness and active sleep in term and near term infants characterized by continuous, low to medium amplitude mixed frequency activity (25–50 µV) with a predominance of theta and delta and overriding beta activity. Synonym: mixed frequency activity. (See active sleep).	**Activité moyenne**: pattern EEG neonatale di veglia e di sonno attivo nei neonati a termine e quasi a termine, caratterizzato da un'attività continua con frequenze miste, di ampiezza da bassa a media (25-50 µV) con una predominanza dell'attività theta e delta e un'attività beta sovraimposta. Sinonimo: attività a frequenze miste o attività intermedia. (Vedi sonno attivo).

**After-discharge:** (1) EEG seizure pattern following single or repetitive electrical stimulation of a discrete area of the brain via cortical or intracerebral electrodes. (2) Burst of rhythmic activity following a transient such as an evoked potential or a spike.	**After-discharge:** (1) pattern EEG che compare in seguito a stimolazione elettrica, singola o ripetitiva, con elettrodi di superficie o intracerebrali, di un'area circoscritta del cervello; (2) burst di attività ritmica che compare dopo un transiente come un potenziale evocato o una punta.

**Aliasing:** Distortion of the EEG signal leading to misidentification of frequency, which occurs when the signal is sampled at less than twice the highest frequency present. The Nyquist theorem states that sampling rates should be at least twice the highest frequency, but accurate digitization of EEG signals requires even higher sampling rates. Comment: Distortion and aliasing can occur at the Nyquist theorem frequency (see Nyquist theorem, sampling rate).	**Aliasing:** distorsione del segnale EEG che comporta un'errata valutazione della frequenza. Si verifica quando il segnale viene campionato a meno del doppio della frequenza più alta presente nel EEG. Il teorema di Nyquist afferma che i tassi di campionamento dovrebbero essere almeno il doppio della frequenza più alta. In realtà la digitalizzazione accurata dei segnali EEG richiede tassi di campionamento ancora più elevati. Commento: distorsione e aliasing possono verificarsi alla frequenza del teorema di Nyquist (vedi teorema di Nyquist, frequenza di campionamento).

**Alpha band:** Frequency band of 8–13 Hz inclusive. Greek letter: α.	**Banda alfa:** frequenza compresa nella banda 8-13 Hz. Lettera greca: α.

**Alpha rhythm:** Rhythm at 8–13 Hz inclusive occurring during wakefulness over the posterior regions of the head, generally with maximum amplitudes over the occipital areas. Amplitude varies but is mostly below 50 µV in the adult, but often much higher in children. Best seen with the eyes closed, during physical relaxation and relative mental inactivity. Blocked or attenuated by attention, especially visual, and mental effort. Comment: use of term rhythm must be restricted to those rhythms that fulfill these criteria. Activities in the alpha band which differ from the alpha rhythm as regards their topography and/or reactivity, should either have specific appellations (for instance: the mu rhythm and alpha coma) or should be referred to as rhythms of alpha frequency or alpha activity. (See blocking, attenuation). Synonym: posterior dominant rhythm.	**Ritmo alfa:** ritmo compreso tra 8-13 Hz che si presenta durante la veglia sulle regioni posteriori dello scalpo, generalmente con massima ampiezza sulle aree occipitali. L'ampiezza è variabile ma solitamente inferiore a 50 µV nell'adulto, mentre è spesso molto più alta nei bambini. Si osserva meglio ad occhi chiusi, nelle fasi di rilassamento fisico e durante la relativa inattività mentale. Bloccato o attenuato dall'attenzione, soprattutto visiva, e dallo sforzo mentale. Commento: l'uso del termine ritmo alfa deve limitarsi a quei ritmi che soddisfano i criteri sopraenunciati. Le attività con frequenza compresa nella banda alfa che differiscono dal ritmo alfa per topografia e/o tipo di reattività, devono essere indicate con denominazioni specifiche (esempi: ritmo mu e l'alfa coma) o essere chiamate ritmi di frequenza alfa o attività alfa. (Vedi blocco, attenuazione). Sinonimo: ritmo dominante posteriore.

**Alpha variant rhythms:** An EEG rhythm recorded most prominently over the posterior regions of the head that differs in frequency, but resembles in reactivity, the alpha rhythm. Comments: (1) often at a supra- or sub-harmonic of alpha frequency and may occur when no alpha rhythm is visible. (2) Not to be confused with posterior slow waves of youth. (See fast alpha variant rhythm, slow alpha variant rhythm, and posterior slow waves of youth).	**Alfa variante:** un ritmo EEG più evidente sulle regioni posteriori dello scalpo che differisce in frequenza rispetto al ritmo alfa, ma presenta lo stesso tipo di reattività. Commenti: (1) spesso costituito da una sovra- o sub-armonica della frequenza alfa. Può presentarsi quando il ritmo alfa non è visibile. (2) da non confondere con le onde lente posteriori della adolescenza. (Vedi variante alfa rapida, variante alfa lenta e onde lente posteriori della adolescenza).

**Alpha wave:** Wave with duration of 1/8–1/13 s (77–125 ms).	**Onda alfa:** onda con durata di 1/8-1/13 s (77-125 ms).

**Amplitude, EEG:** Is a measure of the change of EEG signals with respect to the mean value, usually measured in microvolts (µV), and often expressed as the difference between the maximum and minimum deviation (i.e. peak-to-peak), or in rectified EEG from baseline-to-peak. For a variable EEG activity or modulating sinusoidal rhythm a range can be provided. Comment: EEG amplitude depicts the difference in electrical potential between electrode pairs. Size of the output deflections are dependent upon the method of derivation (i.e. montage), inter-electrode distance, and may be distorted by intervening structures, particularly the skull. Synonym: in practice voltage is synonymous with EEG amplitude, and the visual size of waveforms can be manipulated by the display gain (see voltage and display gain).	**Ampiezza, EEG:** è una misura della variazione dei segnali EEG rispetto al valore medio, di solito misurata in microvolt (µV), e spesso espressa come la differenza tra la deviazione massima e minima (cioè picco-picco), o nell’EEG rettificato dalla linea di base al picco. Quando l'attività EEG o un ritmo sinusoidale sono variabili, si può far riferimento a un intervallo di valori. Commento: l'ampiezza EEG descrive la differenza di potenziale elettrico tra una coppia di elettrodi. Le dimensioni delle deflessioni in uscita dipendono dal metodo di derivazione (cioè dal montaggio), dalla distanza tra gli elettrodi e possono essere distorte dalle strutture frapposte tra la corteccia e l'elettrodo esplorante, soprattutto dalle ossa del cranio. Sinonimo: in pratica il voltaggio è sinonimo di ampiezza, e la dimensione con cui vediamo i grafoelementi può essere manipolata modificando il display gain (vedi tensione e display gain).

**Amplitude-integrated EEG (aEEG):** Involves customized display of EEG activity following signal processing which includes an asymmetric band-pass filter (2–15 Hz), logarithmic amplitude display, rectification, smoothing and time compression (such that several hours can be viewed on a screen). Widely used in neonatal intensive care unit monitoring, for example of infants suffering an hypoxic ischemic encephalopathy. Comment: concurrent review of conventional raw EEG traces is recommended. Synonym: cerebral function monitor (CFM).	**EEG integrato in ampiezza (aEEG, amplitude-integrated EEG)**: permette di modificare la visualizzazione dell'attività EEG, dopo l'iniziale elaborazione del segnale, che include: un filtro passa-banda asimmetrico (2-15 Hz), la visualizzazione logaritmica dell'ampiezza, la rettifica, lo smussamento e la compressione temporale (per permettere, per esempio, la visualizzazione di diverse ore di registrazione sulla stessa pagina dello schermo). Ampiamente utilizzato nel monitoraggio in terapia intensiva neonatale, per esempio dei neonati con encefalopatia ipossico-ischemica. Commento: si raccomanda l’analisi concomitante delle tracce EEG convenzionali. Sinonimo: monitoraggio della funzione cerebrale (CFM, cerebral function monitoring).

**Analog-to-digital conversion (AD conversion):** Transformation of a continuous, analog signal EEG into its digital representation (a discontinuous series of discrete amplitudes). AD conversion is characterized by the sampling rate, which is the number of times per second at which the signal is transformed into digits, and the amplitude resolution, the number of numerical values which can be distinguished within the dynamic range of the system (usually expressed as the number of binary digits). (See sampling rate).	**Conversione analogico-digitale (conversione AD):** trasformazione di un segnale analogico continuo EEG nella sua rappresentazione digitale (una serie discontinua di ampiezze discrete). La conversione AD è caratterizzata da una frequenza di campionamento, che corrisponde al numero di volte al secondo in cui il segnale elettrico in ingresso viene tradotto in sequenza numerica; la risoluzione di ampiezza, ossia il numero di valori numerici che possono essere distinti all'interno della gamma dinamica del sistema (è solitamente espresso in cifre binarie). (Vedi frequenza di campionamento).

**Anterior (slow) dysrhythmia:** A normal EEG activity seen in term and near term infants at 32–44 weeks of post menstrual age; characterized by bilateral frontal delta waves (50–100 µV), seen in isolation or brief runs, typically synchronously and symmetrically.	**Disritmia lenta anteriore:** un'attività EEG normale osservata nei neonati a termine e quasi a termine, a 32-44 settimane di età gestazionale; caratterizzata da onde delta sulle derivazioni frontali, bilaterali (ampiezza 50-100 µV), isolate o raggruppate in brevi sequenze, tipicamente sincrone e simmetriche.

**Application, electrode:** The process of establishing fixation and electrical connection between an electrode and the subject’s scalp or brain.	**Applicazione, elettrodo:** il processo con cui si fissa un elettrodo stabilendo un contatto con lo scalpo o il tessuto cerebrale del soggetto.

**Arrhythmic activity:** A sequence of EEG waves with an inconstant periodicity. (See rhythmic).	**Attività aritmica:** una sequenza di onde EEG con una periodicità incostante. (Vedi ritmico).

**Arousal:** Change from a lower to a higher level of alertness as manifest in EEG activity.	**Risveglio**: attività EEG che compare in relazione al passaggio da un livello di vigilanza inferiore ad uno più alto.

**Array, electrode:** A regular arrangement of electrodes over the scalp or brain, or within the brain substance. Synonym: electrode montage.	**Matrice, elettrodo:** concatenamento/disposizione regolare di elettrodi sullo scalpo, sulla superficie corticale o all'interno del cervello. Sinonimo: montaggio degli elettrodi.

**Artifact:** (1) A physiological potential difference due to an extracerebral source present in EEG recordings, such as eye blinks and movements, electrocardiogram (ECG) or muscle contractions (EMG). (2) A modification of the EEG caused by extracerebral factors, such as instrumental distortion or malfunction, movement of the patient, or ambient electrical noise.	**Artefatto:** (1) una differenza di potenziale di origine biologica, presente nelle registrazioni EEG, dovuta a una fonte extracerebrale, come l'apertura/chiusura degli occhi od altri movimenti oculari, l'elettrocardiogramma (ECG) o le contrazioni muscolari (EMG); (2) una modificazione dell'EEG causata da fattori extracerebrali, come distorsione o malfunzionamento strumentale, movimento del paziente o interferenza elettrica ambientale.

**Asymmetry:** Unequal amplitude, frequency or morphology of EEG activity in channels over homologous areas of the two hemispheres. To be considered abnormal in practice if the amplitude difference exceeds 50% or frequency difference is equal to or greater than 1 Hz in the posterior dominant rhythm, although it should be recognized that these are essentially arbitrary values. It can be quantified by quantitative EEG (qEEG) measures, such as the Brain Symmetry Index (BSI). (See quantitative EEG).	**Asimmetria:** differente ampiezza, frequenza o morfologia dell'attività EEG sulle derivazioni corrispondenti ad aree omologhe dei due emisferi. Dal punto di vista pratico è da considerarsi anormale se la differenza di ampiezza supera il 50% o se la differenza di frequenza è uguale o superiore a 1 Hz nel ritmo dominante posteriore, sebbene si tratti di parametri arbitrari. Può essere calcolata attraverso sistemi di misurazioni EEG quantitativi (qEEG, quantitative EEG) come il Brain Symmetry Index (BSI). (Vedi EEG quantitativo).

**Asynchrony:** The non-coherent occurrence of EEG activities over regions on the same or opposite sides (hemispheres) of the head. For example, two similar waveforms occurring at separate electrodes or channels, but not simultaneously due to a time lag between the channels.	**Asincronia:** la presenza di attività EEG, non temporalmente sincrone tra loro, su regioni dello stesso lato o sui due emisferi. Per esempio, due onde di morfologia simile, registrate su elettrodi o derivazioni separati, ma che non sono attribuibili al semplice ritardo di fase temporale tra canali contigui.

**Attenuation:** Reduction in amplitude of EEG activity (for example, the alpha rhythm is usually attenuated or blocked on eye opening). May occur transiently in response to a physiological or other stimulus, such as electrical stimulation of the brain, or more permanently as a result of pathological conditions, such as cerebral atrophy or ischemia.	**Attenuazione:** riduzione dell'ampiezza dell'attività EEG (per esempio, il ritmo alfa è solitamente attenuato o bloccato dall'apertura degli occhi). Può verificarsi solo transitoriamente in risposta a uno stimolo fisiologico o di altra natura, come la stimolazione elettrica del cervello, o permanentemente, come risultato di condizioni patologiche quali l'atrofia cerebrale o l'ischemia.

**Auditory evoked potential (AEP):** Evoked potential in response to auditory stimulus (see evoked potential, brainstem auditory evoked potential).	**Potenziale evocato uditivo (AEP, auditory evoked potential):** potenziale evocato in risposta ad uno stimolo uditivo (vedi potenziale evocato, potenziale evocato uditivo del tronco encefalico).

**Augmentation:** Increase in amplitude of EEG activity (for example the alpha rhythm is characteristically augmented upon closing the eyes).	**Rinforzo:** aumento dell'ampiezza dell'attività EEG (per esempio l'ampiezza del ritmo alfa è caratteristicamente maggiore in coincidenza della chiusura degli occhi).

**Average (potential) reference electrode:** Use of term discouraged. Term suggested: common average reference. Synonym: Goldman-Offner reference (use of term also discouraged).	**Elettrodo di referenza media (di potenziale):** l'uso del termine è sconsigliato. Termine suggerito: referenza media comune. Sinonimo: referenza di Goldman-Offner (anche l'uso di questo termine è sconsigliato).

**Background activity:** Any underlying EEG activity representing the setting in which focal or transient activity, either normal or abnormal, appears and from which such underlying pattern is distinguished. Comment: there may be no background activity and it is not a synonym of an individual rhythm, such as the alpha rhythm. Synonym: on-going activity.	**Attività di fondo: **qualsiasi attività EEG che costituisca il contesto di base su cui si inscrivono, distinguendosi, l'attività focale o transitoria, normale o anormale. Commento: può non esserci attività di fondo e non è un sinonimo di un ritmo individuale/specifico, come il ritmo alfa. Sinonimo: attività in corso.

**Background slowing:** The frequency of the background rhythm which is below the normal value for age and state. Comment: not to be confused with slowing of the posterior dominant rhythm.	**Rallentamento di fondo:** frequenza del ritmo di fondo al di sotto del valore normale per età e stato. Commento: da non confondere con il rallentamento del ritmo dominante posteriore.

**Band:** Range of EEG frequency in a spectrum for a given recording or epoch, i.e. delta, theta, alpha, beta, gamma bands and high frequency oscillations.	**Banda:** intervallo di frequenze EEG in cui vengono classificate le attività elettriche rilevate in una data registrazione o epoca, per esempio bande delta, theta, alfa, beta, gamma e oscillazioni ad alta frequenza.

**Bandwidth:** The stated range of frequencies (for example 1–70 Hz) between which the response of an EEG channel is within, determined largely by the filters (see frequency response).	**Larghezza di banda:** l'intervallo stabilito delle frequenze (es. 1-70 Hz) entro cui le attività elettriche vengono rilevate da un canale EEG; è determinata in gran parte dai filtri (vedi risposta in frequenza).

**Basal electrode:** Any electrode located in proximity to the base of the skull (see foramen ovale electrode, nasopharyngeal electrode, sphenoidal electrode).	**Elettrodo basale:** qualsiasi elettrodo situato in prossimità del basicranio (vedi elettrodo del forame ovale, elettrodo nasofaringeo, elettrodo sfenoidale).

**Baseline:** (1) Strictly: line obtained when an identical voltage is applied to the two input terminals of an EEG amplifier or the “zero amplitude value” (assumed or perceived) in a given EEG trace or epoch. (2) Loosely: imaginary line corresponding to the approximate mean values of the EEG activity assessed visually in an EEG derivation over a period of time.	**Linea di base:** (1) in senso stretto: la linea ottenuta quando un voltaggio identico è applicato ai due terminali di ingresso di un amplificatore EEG, o linea corrispondente al “valore di ampiezza zero” (presunto o percepito) in una data traccia o epoca EEG; (2) in senso lato: la linea immaginaria corrispondente ai valori medi approssimativi dell'attività EEG valutata visivamente in una derivazione EEG in un determinato periodo di tempo.

**Benign epileptiform discharges of childhood:** Use of term discouraged. Terms suggested, depending on topographical location, occipital, centro-temporal or rolandic spikes (see rolandic spikes).	**Scariche epilettiformi benigne dell'infanzia:** l'uso di questo termine è sconsigliato. Termini suggeriti, a seconda della localizzazione topografica: punte occipitali, centro-temporali o rolandiche (vedi punte rolandiche).

**Benign epileptiform transient of sleep (BETS):** Use of term discouraged. A normal variant. Small sharp spikes of very short duration (<50 ms) and low amplitude (<50 µV), often followed by a small theta wave, occurring in the temporal regions during drowsiness and light sleep. Comment: this pattern is of no clinical significance and in spite of the name is in fact not epileptiform. Synonym: small sharp spikes (preferred term).	**Transiente epilettiforme benigno del sonno (BETS, benign epileptiform transient of sleep):** uso di questo termine sconsigliato. Variante fisiologica. Piccole punte aguzze di brevissima durata (<50 ms) e di bassa ampiezza (<50 µV), spesso seguite da una piccola onda theta, che si presentano sulle derivazioni temporali durante la sonnolenza e il sonno leggero. Commento: questo pattern non ha alcun significato clinico e, nonostante il nome, non è di fatto epilettiforme. Sinonimo: piccole punte aguzze (termine preferito).

**Beta band:** Frequency band of >13–30 Hz inclusive. Greek letter: β.	**Banda beta:** banda di frequenza compresa tra i 13-30 Hz. Lettera greca: β.

**Beta rhythm or activity:** Any EEG rhythm between > 13 and 30 Hz (wave duration > 33–76 ms). Most characteristically recorded over the fronto-central regions of the head during wakefulness. Amplitude of fronto-central beta rhythm varies but is mostly below 30 µV. Blocking or attenuation of the beta rhythm by contralateral movement or tactile stimulation is especially obvious in electrocorticograms. Other beta rhythms are most prominent in other locations or are diffuse, and may be drug-induced (for example alcohol, barbiturates, benzodiazepines and intravenous anaesthetic agents).	**Ritmo o attività beta:** ritmo EEG tra i 13 e 30 Hz (durata dell'onda 33-76 ms). Si registra più caratteristicamente sulle regioni fronto-centrali dello scalpo durante la veglia. L'ampiezza del ritmo beta fronto-centrale varia ma è per lo più inferiore a 30 µV. Il blocco o l'attenuazione del ritmo beta con il movimento o la stimolazione tattile controlaterale sono caratteristiche particolarmente evidenti negli elettrocorticogrammi. Altri ritmi beta sono per lo più evidenti in altre localizzazioni o sono diffusi, e possono essere indotti da farmaci (per esempio alcol, barbiturici, benzodiazepine e agenti anestetici endovenosi).

**Bilateral**: Involving both sides of the head (or body).	**Bilaterale:** interessa entrambi i lati dello scalpo (o del corpo).

**Bilateral independent periodic discharges (BIPDs):** BIPDs are 2 bilateral independent (i.e. asynchronous) surface-negative bi- or di-, tri- or poly-phasic complexes consisting of spike, sharp, or polyspike components, with variably following slow waves, lasting 60–600 ms (typically 200 ms) that occupy at least 50% of a standard 20 minute EEG. Amplitude ranges from 50 to 150 µV (occasionally up to 300 μV), which may be asymmetric, usually recurring at 0.5–2 c/s (but very occasionally with intervals of up to 10 s). BIPDs are broadly distributed and waveform morphology stays fairly constant for a given patient and EEG, with intervening background activity usually attenuated and slow. Most BIPDs are ephemeral phenomena that usually resolve within weeks. They occur with acute marked focal destructive lesions (for example cerebral infarcts, tumors or herpes simplex encephalitis) or more subacute/chronic pathologies (for example epilepsy and vascular compromise). Synonyms: bilateral independent periodic epileptiform discharges, cerebral bigeminy (use of terms discouraged). (See periodic discharges).	**Scariche periodiche bilaterali indipendenti (BIPD, bilateral independent periodic discharges): **le BIPD sono 2 complessi bilaterali indipendenti (cioè asincroni) bi-, di-, tri- o poli-fasici, prevalentemente (N.d.T.) negativi, con componenti a tipo punta, onda puntuta o polipunta, incostantemente seguiti da onde lente, della durata di 60-600 ms (in genere 200 ms), e che occupano almeno il 50% di un EEG standard di 20 minuti. L'ampiezza varia da 50 a 150 µV (occasionalmente fino a 300 μV), possono essere asimmetrici, solitamente ricorrenti a 0,5-2 Hz (ma molto occasionalmente con intervalli fino a 10 s). Le BIPD sono ampiamente distribuite e la morfologia dei grafoelementi rimane abbastanza costante per un dato paziente ed EEG, con la contestuale attività di fondo solitamente attenuata e lenta. La maggior parte delle BIPD sono fenomeni transitori che di solito si risolvono in poche settimane. Si verificano secondariamente a lesioni distruttive focali acute (per esempio infarti cerebrali, tumori o encefalite da herpes simplex) o a patologie più subacute/croniche (per esempio epilessia o insulto vascolare). Sinonimi: scariche epilettiformi periodiche bilaterali indipendenti; bigeminismi cerebrali (l'uso dei termini è sconsigliato). (Vedi scariche periodiche).

**Bilateral independent periodic lateralized epileptiform discharges (BIPLEDs)**: Use of term discouraged. See bilateral independent periodic discharges (preferred term).	**Scariche epilettiformi periodiche lateralizzate bilaterali indipendenti (BIPLEDs, bilateral independent periodic lateralized epileptiform discharges):** l'uso del termine è sconsigliato. Vedi scariche periodiche bilaterali indipendenti (termine preferito).

**Bin width:** Time, usually expressed in milliseconds (ms), elapsing between two successive sampling points in digital EEG (see digital EEG). Synonym: ordinate period.	**Larghezza del bin:** tempo, solitamente espresso in millisecondi (ms), che intercorre tra due punti di campionamento successivi nell'EEG digitale (vedi EEG digitale). Sinonimo: periodo ordinale.

**Biological calibration:** See calibration and common EEG input test.	**Calibrazione biologica: **vedi calibrazione e test di ingresso EEG comune.

**Biphasic wave:** Complex consisting of two wave components developed on alternate sides of the baseline. Synonym: diphasic wave.	**Onda bifasica:** complesso costituito da due componenti d'onda sviluppate sui lati alterni della linea di base. Sinonimo: onda difasica.

**Bipolar derivation:** (1) Recording from a pair of exploring electrodes. (2) Method of organizing the linkages of electrodes to recording channels. (See exploring electrode, bipolar montage, channel).	**Derivazione bipolare: **(1) registrazione da una coppia di elettrodi esploranti; (2) metodo di organizzazione dei collegamenti degli elettrodi ai canali di registrazione. (Vedi elettrodo esplorante, montaggio bipolare, canale).

**Bipolar montage:** Multiple bipolar channels, with no electrode being common to all channels. In most instances, bipolar channels are linked, i.e. adjacent channels from electrodes along the same line of electrodes, or chain, have one electrode in common; so the reference electrode (input terminal 2) in one channel becomes the exploring electrode (input terminal 1) in the next channel of the chain (see channel, reference and exploring electrode).	**Montaggio bipolare:** montaggio con canali bipolari multipli, senza elettrodo comune a tutti i canali. Nella maggior parte dei casi, i canali bipolari sono collegati, ossia canali adiacenti connessi ad elettrodi lungo la stessa linea o catena, hanno un elettrodo in comune; così l'elettrodo di referenza (terminale di ingresso 2) in un canale diventa l'elettrodo esplorante (terminale di ingresso 1) nel canale successivo della catena (vedi canale, elettrodo di riferimento ed esplorante).

**Blocking:** (1) Apparent, temporary obliteration or attenuation of EEG rhythms in response to physiological or other stimuli, such as eye opening on alpha rhythm, or a change in state (see attenuation). (2) A condition of temporary EEG amplifier saturation, caused when the output voltages of the amplifier exceed its operating range sensitivity (see clipping).	**Blocco:** (1) apparente, temporanea scomparsa o attenuazione dei ritmi EEG in risposta a stimoli fisiologici o di altro tipo, come l'apertura degli occhi per il ritmo alfa, o un cambiamento di stato (vedi attenuazione); (2) una condizione di saturazione temporanea dell'amplificatore EEG, che si verifica quando i voltaggi di uscita superano la dinamica dell'amplificatore (vedi clipping).

**Brainstem auditory evoked potential (BAEP):** Far-field auditory evoked potentials generated largely in the brainstem in response to click stimulus and recorded from the surface as a result of volume conduction (see evoked potential, far-field potential, volume conduction).	**Potenziale evocato uditivo del tronco encefalico (BAEP, brainstem auditory evoked potential):** potenziali evocati uditivi far-field (lett. campo lontano), generati in gran parte nel tronco encefalico, in risposta allo stimolo acustico (clic); vengono registrati dalla superficie encefalica come risultato della conduzione di volume (vedi potenziale evocato, potenziale far-field, conduzione di volume).

**Breach rhythm:** EEG activity recorded over or nearby a defect in the skull vault (for example after a fracture, burr hole or craniotomy), of increased amplitude when compared to homologous areas on the opposite side of the head (usually by a factor of less than 3). The rhythm is composed of fast activity with a spiky appearance along with alpha and/or mu rhythms, due to lack of attenuation and distortion by the skull. Comment: a physiological variant to be distinguished from epileptiform activity, although it may be associated with underlying brain injury and therefore a liability to focal seizures.	**Ritmo di breccia:** attività EEG registrata in corrispondenza o nelle vicinanze di un difetto nella teca cranica (per esempio dopo una frattura, un foro o una craniotomia). Mostra ampiezza aumentata rispetto ad aree omologhe controlaterali (di solito di un fattore inferiore a 3). Morfologicamente è composto da un'attività rapida con un aspetto puntuto frammisto a ritmi alfa e/o mu, a causa della mancanza di attenuazione e distorsione da parte del cranio. Commento: una variante fisiologica da distinguere dall'attività epilettiforme, anche se può essere associata a una lesione cerebrale sottostante e quindi a una predisposizione a crisi focali.

**Buffer amplifier:** An amplifier, generally with a voltage gain of 1, a high input impedance, and a low output impedance, used to isolate the input signal from the loading effects of an immediately following circuit. In some electroencephalographs, each input is connected to a buffer amplifier located in the jack box to reduce cable artifact and interference.	**Amplificatore tampone:** un amplificatore, generalmente con un guadagno di voltaggio di 1, con un'alta impedenza di ingresso e una bassa impedenza di uscita, utilizzato per isolare il segnale di ingresso dagli effetti di carico di un circuito immediatamente successivo. In alcuni elettroencefalografi, ogni ingresso è collegato a un amplificatore tampone situato nella scatola dei connettori per ridurre l'artefatto e le interferenze del cavo.

**Build-up:** Colloquialism. Used to describe progressive increase in voltage of EEG activity or the appearance of slow waves of increasing amplitude. For example during hyperventilation when often associated with decrease in frequency. Sometimes applied to seizure pattern (see seizure pattern).	**Aumento:** colloquiale. Usato per descrivere il progressivo aumento di voltaggio dell'attività EEG o la comparsa di onde lente di ampiezza crescente. Per esempio, durante l'iperventilazione, quando è spesso associato ad una diminuzione della frequenza. A volte può costituire il pattern di una crisi (vedi pattern delle crisi).

**Burst:** A group of waves with a minimum of four phases and duration longer than 500 ms which appear and disappear abruptly and are distinguished from background activity by differences in frequency, form and/or amplitude. Comments: (1) term does not imply abnormality. (2) Not a synonym of paroxysm (see paroxysm).	**Burst: **un gruppo di onde con un minimo di quattro fasi e una durata superiore a 500 ms che appaiono e scompaiono bruscamente e si distinguono dall'attività di fondo per differenze di frequenza, forma e/o ampiezza. Commenti: (1) il termine non implica anormalità; (2) non è un sinonimo di parossismo (vedi parossismo).

**Burst suppression:** Pattern characterized by paroxysmal bursts of theta and/or delta waves, at times intermixed with sharp and faster waves, alternating with intervening periods of attenuation or suppression (below 10 µV) lasting more than 50% of the record. Comments: EEG pattern that indicates either severe brain dysfunction or is typical for some anesthetic drugs at certain levels of anesthesia. Comment: burst suppression pattern with identical bursts after anoxic brain injury has been reported to portend a poor neurological prognosis.	**Burst suppression: **pattern caratterizzato da burst parossistiche di onde theta e/o delta, a volte mescolate a onde puntute più rapide, alternate a periodi di attenuazione (< 20 µV) o soppressione (< 10 µV), che durano più del 50% della registrazione. Commenti: pattern EEG che indica una grave disfunzione cerebrale oppure è l’effetto di alcuni farmaci anestetici a certi livelli di anestesia. Commento: si ritiene che il pattern burst suppression con burst identiche, secondario ad una lesione cerebrale anossica, sia predittivo di una prognosi neurologica negativa.

**Calibration**: Historically, analogue procedure of testing and recording the responses of EEG channels to voltage differences applied to the input terminals of their respective amplifiers. DC (usually) or AC voltages of magnitude comparable to the amplitudes of EEG waves are used in this procedure. In the digital era, instrumental system calibration is either performed with an external signal generator or verified by an internal signal generator within the instrument, which is governed by the software of the system.	**Calibrazione:** storicamente, procedura analogica di prova e registrazione delle risposte dei canali EEG alle differenze di voltaggio applicate ai terminali di ingresso dei rispettivi amplificatori. In questa procedura vengono utilizzati voltaggi a corrente diretta (più spesso) o a corrente alternata di ampiezza paragonabile alle ampiezze delle onde EEG. Nell'era digitale, la calibrazione del sistema strumentale viene eseguita con un generatore di segnale esterno o verificata da un generatore di segnale interno allo strumento, che è governato dal software del sistema.

**Cap, head**: A cap that is fitted over the head to hold electrodes in position. Synonym: electrode cap.	**Cuffia, testa:** una cuffia che viene applicata sullo scalpo per tenere gli elettrodi in posizione. Sinonimo: cuffia per elettrodi.

**Channel**: Complete system for the detection, amplification and display of potential differences between a pair of electrodes, or a computed reference (for example common average reference). Digital EEG machines simulate a multichannel display by tracing several voltage time plots on a visual display.	**Canale:** sistema completo per il rilevamento, l'amplificazione e la visualizzazione delle differenze di potenziale tra una coppia di elettrodi o tra un elettrodo e una referenza calcolata (per esempio la referenza media comune). Le macchine EEG digitali simulano una visualizzazione multicanale tracciando diversi diagrammi temporali di voltaggio su un display visivo.

**Circumferential bipolar montage**: A montage consisting of linked bipolar derivations encircling the head. Commonly bilateral longitudinal temporal electrode chains are linked together.	**Montaggio bipolare circonferenziale:** un montaggio costituito da derivazioni bipolari interconnesse che circondano lo scalpo. Comunemente vengono collegate tra loro le catene di elettrodi longitudinali temporali dei due lati.

**Clipping**: Distortion of the recorded signal which makes it appear flat-topped in the display, caused by excess output voltage overloading the amplifiers (see blocking).	**Clipping:** distorsione del segnale registrato che ne determina un appiattimento sul display; causata da un eccesso di tensione di uscita che sovraccarica gli amplificatori (vedi blocco).

**Closely spaced electrodes**: Additional scalp electrode placed at a shorter distance than that specified by the International ten-twenty system (for example see the ten-ten system).	**Elettrodi ravvicinati:** elettrodo da scalpo supplementare posto a una distanza inferiore a quella specificata dal sistema internazionale dieci-venti (per esempio vedi il sistema dieci-dieci).

**Common average reference**: Computational average potential of all or most electrode signals used as a reference electrode. Synonyms: average (potential) reference and Goldman-Offner electrode (use of terms discouraged). (See reference electrode, Laplacian montage).	**Referenza media comune:** potenziale medio calcolato su tutti o sulla maggior parte dei segnali degli elettrodi, usato come referenza. Sinonimi: referenza media (di potenziale) ed elettrodo di Goldman-Offner (l'uso dei termini è sconsigliato). (Vedi elettrodo di referenza, montaggio laplaciano).

**Common EEG input test**: Procedure in which the same pair of EEG electrodes is connected to the two input terminals of all channels of the electroencephalograph. Comment: used as adjunct to calibration procedure (see calibration). Synonym: biological calibration.	**Test di ingresso EEG comune:** procedura in cui la stessa coppia di elettrodi EEG è collegata ai due terminali di ingresso di tutti i canali dell'elettroencefalografo. Commento: usato come aggiunta alla procedura di calibrazione (vedi calibrazione). Sinonimo: calibrazione biologica.

**Common mode rejection**: A characteristic of differential amplifiers whereby they provide markedly reduced amplification of common mode signals, compared to differential signals. Expressed as common mode rejection ratio (CMRR), i.e. ratio of amplifications of differential and common mode signals. Example:amplification,differential/amplification,commonmode=100,000/1=100,000:1	**Reiezione a modo comune:** una caratteristica degli amplificatori differenziali per la quale questi riducono l'ampiezza dei segnali a modo comune, rispetto ai segnali differenziali. Viene espressa come rapporto di reiezione a modo comune (CMRR, common mode rejection ratio), cioè il rapporto delle amplificazioni dei segnali differenziali e a modo comune. Esempio:amplificazione,differenziale/amplificazione,modocomune=100.000/1=100.000:1

**Common mode signal**: Common component of the two signals applied to the two input terminals of a differential EEG amplifier. Comment: in EEG recording, external capacitive interference frequently occurs as a common mode signal.	**Segnale a modo comune:** componente comune di due segnali applicati ai due terminali d'ingresso di un amplificatore EEG differenziale. Commento: nella registrazione EEG, l'interferenza capacitiva esterna si presenta spesso come segnale a modo comune.

**Common reference electrode**: A reference electrode that is common to all channels.	**Elettrodo di referenza comune:** un elettrodo di referenza comune a tutti i canali.

**Common reference montage**: A montage in which each of the channels have the same reference electrode (see channel, referential derivation, reference electrode).	**Montaggio con referenza comune:** un montaggio in cui ognuno dei canali ha lo stesso elettrodo di riferimento (vedi canale, derivazione referenziale, elettrodo di riferimento).

**Complex**: A sequence of two or more waveforms having a characteristic composite morphology; and when recurring are seen with a fairly consistent form, distinguished from background activity. (For example see spike-and-slow-wave complex).	**Complesso:** una sequenza di due o più grafoelementi che hanno una morfologia composta in maniera caratteristica; quando ricorrono sono visibili con una forma relativamente riproducibile e appaiono distinte dall'attività di fondo (per esempio vedi complesso di punta-onda-lenta).

**Contingent negative variation (CNV)**: An event-related slow negative potential elicited in the interval between a conditional stimulus and an associated contingent second ‘imperative’ stimulus, to which the subject is required to make a voluntary response. It comprises a progressive negative-going change maximal at the vertex, which requires special recording techniques so not seen in conventional EEG recording. Synonym: “expectancy wave”. (See event-related potential).	**Variazione negativa contingente (CNV, contingent negative variation):** un potenziale negativo lento evento-correlato elicitato nell'intervallo tra uno stimolo condizionante e un secondo stimolo condizionato “imperativo” associato, al quale il soggetto è tenuto a dare una risposta volontaria. È costituito da una modificazione progressiva in polarità negativa, massima al vertice, che richiede tecniche di registrazione speciali e non si vede nella registrazione EEG convenzionale. Sinonimo: “onda di attesa”. (Vedi potenziale evento-correlato).

**Continuous EEG (cEEG)**: Prolonged EEG recording and often analysis for monitoring of electrical brain activity. Data may be collected with analog or digital systems, the latter enabling post-acquisition processing with a number of quantitative EEG techniques. cEEG is generally practiced in the intensive care unit with application varying according to the clinical situation: monitoring the EEG as a surrogate of cerebral metabolism (to detect hypoxia or ischemia), nonconvulsive seizures or status epilepticus, and to monitor the effects of treatment. The aim is to detect EEG changes when the cerebral dysfunction is reversible. There have been recommendations that cEEG is used to improve prognostication of coma after cardiac arrest. (See quantitative EEG).	**EEG continuo (cEEG, continuous EEG):** registrazione EEG prolungata, spesso accompagnata da analisi EEG, per il monitoraggio dell'attività elettrica cerebrale. I dati possono essere raccolti con sistemi analogici o digitali. Questi ultimi consentono l'elaborazione post-acquisizione con una serie di tecniche EEG quantitative. È generalmente utilizzato nelle unità di terapia intensiva, con applicazioni variabili a seconda del contesto clinico: monitoraggio dell'EEG come surrogato del metabolismo cerebrale (per rilevare ipossia o ischemia), crisi non convulsive o stato epilettico, monitorare gli effetti dei trattamenti. L'obiettivo è quello di rilevare tempestivamente i cambiamenti EEG in caso di disfunzione cerebrale reversibile. Il cEEG è raccomandato per migliorare la valutazione prognostica del coma post arresto cardiaco. (Vedi EEG quantitativo).

**Continuous slow activity**: Uninterrupted ongoing slow activity (theta and delta bands) which may be rhythmic, arrhythmic or polymorphic, which may wax and wane but not regress, and is of variable amplitude and morphology. Typically it is non-responsive to external stimuli and clearly exceeds the amount considered physiologically normal for the patient’s age. (See intermittent slow activity).	**Attività lenta continua:** attività lenta ininterrotta e persistente (bande theta e delta) che può essere ritmica, aritmica o polimorfa, può aumentare o diminuire ma non regredire, e di ampiezza e morfologia variabile. Tipicamente non risponde a stimoli esterni e supera chiaramente la quantità di attività lenta considerata fisiologicamente normale per l'età del paziente. (Vedi attività lenta intermittente).

**Continuous spike and waves during sleep (CSWS):** An epileptic encephalopathy syndrome of electrical status epilepticus during sleep (ESES) associated with neurocognitive dysfunction. Seizures are typically infrequent. Comment: often used interchangeably with electrical status epilepticus during sleep (ESES). Synonyms: encephalopathy with status epilepticus during slow sleep. (See slow wave sleep, electrical status epilepticus during sleep).	**Punta-onda continue durante il sonno (CSWS, continuous spike and waves during sleep):** una sindrome da encefalopatia epilettica con stato epilettico elettrico durante il sonno (ESES, electrical status epilepticus during sleep) associata a disfunzione cognitiva. Le crisi sono tipicamente poco frequenti. Commento: spesso usato in modo intercambiabile con ESES. Sinonimi: encefalopatia con stato epilettico durante il sonno lento. (Vedi sonno ad onde lente, stato epilettico elettrico durante il sonno).

**Coronal bipolar montage**: Synonym: see transverse bipolar montage.	**Montaggio bipolare coronale:** sinonimo: vedi montaggio bipolare trasversale.

**Cortical electrode**: Electrode applied directly upon or inserted in to the cerebral cortex.	**Elettrodo corticale:** elettrodo applicato direttamente sulla corteccia cerebrale o inserito in essa.

**Cortical electroencephalogram**: See electrocorticogram.	**Elettroencefalogramma corticale:** vedi elettrocorticogramma.

**Cortical electroencephalography**: See electrocorticography.	**Elettroencefalografia corticale:** Vedi elettrocorticografia.

**Cycle**: The complete sequence of recurrent almost sinusoidal oscillatory potential changes undergone by individual waveform components of regularly repeated EEG waves or complexes.	**Ciclo:** sequenza completa dei cambiamenti di un'onda, di un grafoelemento semplice o di complessi EEG che si ripetono identici ad intervalli di tempo regolari; periodo di tempo necessario al loro compiersi.

**Cycles per second (c/s)**: Unit of frequency defined as the number of complete cycles in one second. Synonym: hertz (Hz). (See frequency).	**Cicli al secondo (c/s):** unità di frequenza definita come il numero di cicli completi in un secondo. Sinonimo: Hertz (Hz). (Vedi frequenza).

**Deep sleep**: Non-REM sleep stage N3 is dominated (≥20%) by slow delta waves of frequency 0.5 to 2 Hz and peak-to-peak amplitude >75 µV, measured over the frontal regions. Synonym: slow wave sleep. (See light sleep).	**Sonno profondo:** la fase N3 del sonno NREM è dominata (≥20%) da onde delta lente di frequenza da 0,5 a 2 Hz e ampiezza da picco a picco >75 µV, misurate sulle regioni frontali. Sinonimo: sonno a onde lente. (Vedi sonno leggero).

**Delta band**: Frequency band of 0.1–<4 Hz. Greek letter: δ. Comment: for practical purposes lower frequency limit is 0.5 Hz, as DC potential differences are not monitored in conventional EEGs.	**Banda delta**: banda di frequenza di 0,1–<4 Hz. Lettera greca: δ. Commento: ai fini pratici il limite inferiore di frequenza è 0,5 Hz, poiché le differenze di potenziale in corrente continua (DC) non sono registrabili negli EEG convenzionali.

**Delta brush**: Normal neonatal graphoelement, seen at 26–40 weeks of post menstrual age (PMA), maximal around 32–34 weeks, and rare at term; combination of delta wave (0.3–1.5 c/s; 50–300 µV) with superimposed fast activity (>8 Hz; 10–60 µV). Localization changes with PMA. Synonyms: ripples of prematurity, spindle-delta bursts (use of terms discouraged). (See also extreme delta brush).	**Delta brush**: normale grafoelemento neonatale, osservabile tra le settimane 26-40 di età gestazionale, con massima espressione a 32-34 settimane, e più raramente a termine; combinazione di onda delta (0,3-1,5 Hz; 50-300 µV) con attività rapida sovraimposta (>8 Hz; 10-60 µV). La localizzazione è variabile e dipende dall’epoca gestazionale. Sinonimi: onda lenta increspata (della prematurità), complessi fuso-delta (l'uso dei termini è sconsigliato). (Vedi anche extreme delta brush).

**Delta wave**: Wave with duration of ¼-2 s (250–2000 ms).	**Onda delta:** onda con durata di ¼-2 s (250-2000 ms).

**Depth electrode**: Electrode (usually a multicontact electrode) implanted within the brain substance.	**Elettrodo di profondità:** elettrodo (di solito un elettrodo multicontatto) impiantato all'interno della sostanza cerebrale.

**Depth electroencephalogram**: Recording of electrical activity of the brain by means of electrodes implanted within the brain substance, usually deep structures such as both hippocampi (see for example stereotactic [stereotaxic] depth electroencephalogram).	**Elettroencefalogramma di profondità:** registrazione dell'attività elettrica cerebrale con elettrodi impiantati direttamente all'interno della sostanza cerebrale; di solito si tratta di strutture profonde come gli ippocampi (vedi per esempio elettroencefalogramma di profondità stereotassico).

**Depth electroencephalography**: Technique of recording intra-cranial depth electroencephalogram (see for example stereotactic [stereotaxic] depth electroencephalography).	**Elettroencefalografia di profondità:** tecnica di registrazione dell'elettroencefalogramma intracerebrale di profondità (vedi per esempio elettroencefalogramma stereotassico di profondità).

**Derivation**: (1) The process of recording from, or computing voltage differences between, a pair of electrodes in an EEG channel. (2) The EEG record obtained by this process.	**Derivazione:** (1) il processo di registrazione da, o il calcolo delle differenze di voltaggio tra, una coppia di elettrodi in un canale EEG; (2) la registrazione EEG ottenuta da questo processo.

**Desynchronization**: Terms suggested: blocking or attenuation, depending on circumstance. The term desynchronization is acceptable when referring to the mechanisms presumably responsible for blocking or attenuation. It is also used in describing attenuation of a frequency band based on power spectra analysis of the EEG signal (for instance “event-related desynchronization”). (See blocking and attenuation).	**Desincronizzazione:** termini suggeriti: blocco o attenuazione, a seconda delle circostanze. Il termine desincronizzazione è accettabile quando ci si riferisce ai meccanismi presumibilmente responsabili del blocco o dell'attenuazione. È anche usato per descrivere l'attenuazione di una banda di frequenza basata sull'analisi degli spettri di potenza del segnale EEG (per esempio “desincronizzazione legata agli eventi”). (Vedi blocco e attenuazione).

**Diffuse**: Colloquialism: an EEG activity spread over large areas of both sides of the head (see generalized). This does not imply abnormality as a normal rhythm may be diffusely distributed (for example alpha activity in some individuals, or slow waves in deep sleep). Comment: where possible the topographic distribution, symmetry and synchrony should be specified.	**Diffuso:** termine colloquiale: un'attività EEG diffusa su ampie aree di entrambi i lati dello scalpo (vedi generalizzato). Questo non implica di per sé qualcosa di patologico, poiché un ritmo normale può essere distribuito in modo diffuso (per esempio l'attività alfa in alcuni individui, o le onde lente nel sonno profondo). Commento: dove possibile si dovrebbe specificare la distribuzione topografica, la simmetria e la sincronia.

**Differential amplifier**: An amplifier whose output is proportional to the voltage difference between its two input terminals. Comment: electroencephalographs make use of differential amplifiers in their input stages.	**Amplificatore differenziale:** un amplificatore la cui uscita è proporzionale alla differenza di voltaggio tra i suoi due terminali di ingresso. Commento: gli elettroencefalografi fanno uso di amplificatori differenziali nei loro stadi di ingresso.

**Differential signal**: Difference between two signals applied to the respective two input terminals of a differential EEG amplifier.	**Segnale differenziale:** differenza tra due segnali applicati ai rispettivi due terminali d'ingresso di un amplificatore EEG differenziale.

**Digital EEG**: (1) The representation of an analog EEG signal by a series of numbers related to successive measurements of the magnitude of the signal at equal time intervals. (2) The practice of electroencephalography using digital representation of EEGs.	**EEG digitale:** (1) la rappresentazione di un segnale EEG analogico mediante una serie di numeri relativi a misure successive della grandezza del segnale a intervalli di tempo uguali; (2) la pratica dell'elettroencefalografia utilizzando la rappresentazione digitale dell'EEG.

**Diphasic wave**: Complex consisting of two wave components developed on alternate sides of the baseline. Synonym: Biphasic wave.	**Onda difasica:** complesso costituito da due componenti d'onda che si sviluppano sui lati opposti della linea di base. Sinonimo: onda bifasica.

**Dipole**: An EEG signal vector produced by a separation of negative (sink) and positive (source) potential poles (or current). A dipole is characterized by its strength, location and orientation. Depending on their orientation, dipoles can be radial (perpendicular to the surface), tangential (parallel to the surface) or a combination of these (oblique). Comment: an equivalent current dipole is a theoretical construct commonly used in source imaging to model a generator of an EEG signal located in the center of gravity of the source (for example an evoked potential, or an epileptiform discharge). Distributed source models are computed using a large number of small dipoles, distributed within the source space.	**Dipolo:** un vettore di segnale EEG prodotto da una separazione di poli di potenza/potenziale elettrico (o corrente) negativo (dispersore) e positivo (sorgente). Un dipolo è caratterizzato dalla sua potenza, posizione/topografia e orientamento. A seconda del loro orientamento, i dipoli possono essere radiali (perpendicolari alla superficie), tangenziali (paralleli alla superficie) o una combinazione di questi (obliqui). Commento: un dipolo di corrente equivalente è un costrutto teorico comunemente usato nella rappresentazione della sorgente per modellare un generatore di un segnale EEG situato nel centro di gravità della sorgente (per esempio un potenziale evocato, o una scarica epilettiforme). I modelli di sorgente distribuita sono calcolati usando un gran numero di piccoli dipoli, distribuiti nello spazio della sorgente.

**Direct coupled amplifier**: An amplifier in which successive stages are connected (coupled) by devices, the performance of which is not frequency dependent.	**Amplificatore ad accoppiamento diretto:** un amplificatore in cui gli stadi successivi sono collegati (accoppiati) da dispositivi, le cui prestazioni non dipendono dalla frequenza.

**Direct current (DC-) amplifier**: An amplifier that is capable of recording DC (zero frequency) voltages and slowly varying voltages.	**Amplificatore a corrente continua (DC, direct current)**: un amplificatore in grado di registrare voltaggi in corrente continua (frequenza zero) e voltaggi lentamente variabili.

**Discharge:** Waveforms with no more than 3 phases (i.e. crosses the baseline no more than twice) or any waveform lasting 0.5 s or less, regardless of the number of phases. Interpretive term of action potentials and post-synaptic potentials commonly used to designate interictal epileptiform and seizure patterns (see epileptiform pattern, seizure pattern).	**Scarica:** forme d'onda con non più di 3 fasi (cioè attraversa la linea di base non più di due volte) o qualsiasi forma d'onda di durata pari o inferiore a 0,5 s, indipendentemente dal numero di fasi. Termine interpretativo dei potenziali d'azione e dei potenziali postsinaptici comunemente usato per indicare i pattern epilettiformi critici e intercritici (vedi pattern epilettiforme, pattern critico).

**Disk electrode**: Typically a metal disk attached to the scalp with an adhesive such as collodion or adherent conductive paste.	**Elettrodo a coppetta:** un disco di metallo attaccato al cuoio capelluto con una sostanza adesiva come il collodio o una pasta adesiva e conduttiva.

**Disorganization**: Gross alteration in frequency, form, topography and/or quantity of physiologic EEG rhythms in: (1) an individual record, relative to previous records in the same subject or the rhythms of homologous regions on the opposite side of the head, or (2) relative to findings in normal subjects of similar age and similar state of vigilance. (See organization).	**Disorganizzazione:** grossolana alterazione della frequenza, della forma, della topografia e/o della quantità dei ritmi EEG fisiologici in: (1) una singola registrazione, rispetto alle registrazioni precedenti nello stesso soggetto o ai ritmi di regioni omologhe sul lato opposto dello scalpo, o (2) rispetto ai risultati in soggetti normali di età simile e di simile stato di vigilanza. (Vedi organizzazione).

**Display Gain**: Manipulation of the data after acquisition to change the visual size of waveforms in order to aid visual inspection. Comment: results of increase or decrease of the display gain are similar to changes of sensitivity during acquisition.	**Display gain (lett. guadagno di visualizzazione):** manipolazione dei dati dopo la loro acquisizione per modificare la dimensione visiva delle forme d'onda al fine di facilitare l'ispezione visiva. Commento: i risultati dell'aumento o della diminuzione del display gain sono simili ai cambiamenti di sensibilità durante l'acquisizione.

**Distortion:** An instrument-induced alteration of waveform (see artifact, clipping).	**Distorsione**: un'alterazione della forma d'onda indotta dallo strumento (vedi artefatto, clipping).

**Duration:** (1) The interval from beginning to end of an individual wave or complex. Comment: the duration of the cycle of individual components of a sequence of regularly repeating waves or complexes is referred to as the period of the wave or complex. (2) The time that a sequence of waves or complexes or any other distinguishable feature lasts in an EEG record.	**Durata: **(1) l'intervallo dall'inizio alla fine di una singola onda o complesso. Commento: la durata del ciclo dei singoli componenti di una sequenza di onde o complessi che si ripetono regolarmente viene definita periodo dell'onda o del complesso; (2) il tempo di durata, in una registrazione EEG, di una sequenza di onde o di complessi o di qualsiasi altra attività.

**Electrical status epilepticus during sleep (ESES):** an EEG pattern seen in childhood which consists of continuous or near continuous spike-and-slow-waves during sleep. Discharges may be seen in wakefulness, often with a frontal or temporal emphasis, but increase markedly in sleep and typically abate in REM sleep. Quantification of epileptiform activity is not standardized but some use spike-and-slow-wave index of >50% or >85%. Most children have or develop seizures and present with neurocognitive dysfunction. Comment: often used synonymously with continuous spike and waves during sleep (CSWS). (See index, slow wave sleep, continuous spike and waves during sleep).	**Stato epilettico elettrico durante il sonno (ESES, electrical status epilepticus during sleep):** un pattern EEG che si osserva nell'infanzia costituito da complessi punta-onda-lenta continui o subcontinui durante il sonno. Le scariche possono essere osservate in veglia, spesso più evidenti sulle regioni frontali o temporali; aumentano notevolmente nel sonno e tipicamente diminuiscono nel sonno REM. La quantificazione dell'attività epilettiforme non è standardizzata, ma può essere utilizzato un cut-off di rappresentazione sul tracciato di complessi punta-onda-lenta per >50% o >85% della durata di registrazione. La maggior parte dei bambini con ESES hanno o svilupperanno crisi epilettiche, oltre a presentare disturbi neurocognitivi. Commento: spesso usato come sinonimo punta-onda continue durante il sonno (CSWS, continuous spike and waves during sleep). (Vedi indice, sonno a onde lente, punta-onda continue durante il sonno).

**Electrocerebral inactivity:** Absence over all regions of the head of identifiable electrical activity of cerebral origin, whether spontaneous or induced by physiological stimuli or pharmacological agents. Comment: strict technical recording standards should be observed in suspected cerebral death (Stecker et al., 2016). Tracings of electrocerebral inactivity should be clearly distinguished from low voltage EEGs (see low voltage EEG). Synonyms: electrocerebral silence, flat or isoelectric EEG (use of terms discouraged).	**Inattività elettrocerebrale**: assenza su tutte le regioni dello scalpo di attività elettrica identificabile come di origine cerebrale, sia spontanea che indotta da stimoli fisiologici o agenti farmacologici. Commento: in caso di sospetta morte encefalica si devono adottare rigorosi standard tecnici di registrazione (Stecker et al., 2016). I tracciati di inattività elettrocerebrale devono essere chiaramente distinti dagli EEG di basso voltaggio (vedi EEG di basso voltaggio). Sinonimi: silenzio elettrico cerebrale, EEG piatto o isoelettrico (l'uso di questi termini è sconsigliato). N.d.T.: assenza di attività elettrica cerebrale.

**Electrocorticogram (ECoG)**: Record of EEG activity obtained by means of electrodes applied directly over or inserted in to the cerebral cortex. Comment: electrocorticograms can be performed intraoperatively and extraoperatively after surgical implantation (see subdural electrode).	**Elettrocorticogramma (ECoG):** registrazione dell'attività EEG utilizzando elettrodi appoggiati sulla superficie corticale. Commento: gli elettrocorticogrammi possono essere eseguiti durante l'intervento chirurgico o successivamente all'impianto (vedi elettrodo subdurale).

**Electrocorticography (ECoG)**: Technique of recording electrical activity of the brain by means of electrodes applied over or implanted in to the cerebral cortex. Comment: electrocorticography can be performed intraoperatively and extraoperatively after surgical implantation (see subdural electrode).	**Elettrocorticografia (ECoG):** tecnica di registrazione dell'attività elettrica cerebrale attraverso elettrodi appoggiati sulla superficie corticale. Commento: l'elettrocorticografia può essere eseguita durante l'intervento chirurgico o successivamente all'impianto (vedi elettrodo subdurale).

**Electrode, EEG**: A conducting device applied over or inserted in a region of the scalp or brain.	**Elettrodo, EEG:** un dispositivo conduttore applicato su una regione dello scalpo o inserito in una regione del cervello.

**Electrodecrement**: A period of amplitude attenuation usually with superimposed fast activity.	**Elettrodecremento:** un periodo di attenuazione dell'ampiezza di solito associato ad attività rapida sovraimposta.

**Electrode impedance**: Total effective resistance to alternating current (AC), arising from ohmic resistance and reactance. Measured between pairs of electrodes or, in some electroencephalographs, between each individual electrode and all the other electrodes connected in parallel. Expressed in ohms (generally kilo-ohms, kΩ). Comments: (1) over the EEG frequency range, because the capacitance factor is small, electrode impedance is usually equal to electrode resistance. (2) Not a synonym of input impedance of EEG amplifier (see electrode resistance, input impedance).	**Impedenza dell'elettrodo:** resistenza effettiva totale alla corrente alternata (AC, alternating current), derivante dalla resistenza e dalla reattanza. Misurata tra coppie di elettrodi o, in alcuni elettroencefalografi, tra ogni singolo elettrodo e tutti gli altri elettrodi collegati in parallelo. Espressa in ohm (generalmente kilo-ohm, kΩ). Commenti: (1) nella gamma di frequenza EEG, poiché il fattore di capacità è piccolo, l'impedenza dell'elettrodo è solitamente uguale alla resistenza dell'elettrodo; (2) non è un sinonimo di impedenza di ingresso dell'amplificatore EEG (vedi resistenza dell'elettrodo, impedenza di ingresso).

**Electrode resistance**: Total effective resistance to direct current (DC), through the interface between an EEG electrode and the scalp or brain. Measured between pairs of electrodes or, in some electroencephalographs, between each individual electrode and all the other electrodes connected in parallel. Expressed in ohms (generally kilo-ohms, kΩ). Comment: measurement of electrode resistance with DC currents results in varying degrees of electrode polarization (see electrode impedance).	**Resistenza elettrodica:** resistenza effettiva totale alla corrente continua (DC, direct current), attraverso l'interfaccia tra un elettrodo EEG e lo scalpo o il cervello. Misurata tra coppie di elettrodi o, in alcuni elettroencefalografi, tra ogni singolo elettrodo e tutti gli altri elettrodi collegati in parallelo. Espressa in ohm (generalmente kilo-ohm, kΩ). Commento: la misurazione della resistenza dell'elettrodo con correnti continue risulta in diversi gradi di polarizzazione dell'elettrodo (vedi impedenza dell'elettrodo).

**Electroencephalogram (EEG)**: Record of electrical activity of the brain taken by means of electrodes placed on the surface of the head, unless otherwise specified.	**Elettroencefalogramma (EEG)**: registrazione dell'attività elettrica cerebrale attraverso elettrodi posti sullo scalpo, se non diversamente specificato.

**Electroencephalograph (EEG)**: Instrument employed to record electroencephalograms.	**Elettroencefalografo (EEG):** strumento impiegato per registrare gli elettroencefalogrammi.

**Electroencephalographic**: Appertaining to bioelectrical recording, irrespective of the method employed (in the present context EEG, ECoG, SEEG, etc.).	**Elettroencefalografico:** relativo alla registrazione bioelettrica, indipendentemente dal metodo impiegato (nel presente contesto EEG, ECoG, SEEG, ecc.).

**Electroencephalography (EEG)**: (1) The science relating to the electrical activity of the brain. (2) The practice of recording and interpreting electroencephalograms.	**Elettroencefalografia (EEG):** (1) la scienza che studia l'attività elettrica cerebrale; (2) la pratica della registrazione e dell'interpretazione degli elettroencefalogrammi.

**Encoche frontale**: Normal neonatal graphoelement in term and near term infants, between 34 and 44 weeks post menstrual age. Frontal broad diphasic sharp waves (50–100 µV); typically bilateral, but may be unilateral. Usually seen in transition from active to quiet sleep. Synonym: anterior sharp transient, transient frontal sharp wave. (See active and quiet sleep).	**Encoche frontale (lett. incisura frontale):** grafoelemento fisiologico dell'età neonatale, nei neonati a termine e quasi a termine, tra le settimane 34 e 44 di età gestazionale. Onde frontali ampie e difasiche (50-100 µV); tipicamente bilaterale, può anche essere unilaterale. N.d.T.: in alcune occasioni possono presentarsi in maniera ripetitiva (treni/sequenze). Di solito si osserva durante la transizione da sonno attivo a sonno quieto. Sinonimo: incisura frontale, onda aguzza frontale fisiologica, transiente aguzzo anteriore fisiologico (Vedi sonno attivo e sonno quieto).

**Epicortical electrode**: Use of term discouraged. Synonym: subdural electrode (preferred term).	**Elettrodo epicorticale:** uso del termine sconsigliato. Sinonimo: elettrodo subdurale (termine preferito).

**Epidural electrode**: Electrode located over the dural covering of the cerebrum.	**Elettrodo epidurale:** elettrodo posizionato sopra la dura madre.

**Epileptiform pattern**: Describes transients distinguishable from background activity with a characteristic morphology typically, but neither exclusively nor invariably, found in interictal EEGs of people with epilepsy. Epileptiform patterns have to fulfill at least 4 of the following 6 criteria:	**Pattern epilettiforme:** descrive dei grafoelementi, che si stagliano dall'attività di fondo, con una morfologia caratteristica che si trova tipicamente, ma non esclusivamente né invariabilmente, negli EEG intercritici delle persone con epilessia. Un pattern epilettiforme deve soddisfare almeno 4 dei seguenti 6 criteri:

(1) Di- or tri-phasic waves with sharp or spiky morphology (i.e. pointed peak).	(1) Onde di- o tri-fasiche con morfologia puntuta (cioè picco appuntito).

(2) Different wave-duration than the ongoing background activity, either shorter or longer.	(2) Diversa durata dell'onda rispetto all'attività di fondo in corso, sia essa più breve o più lunga.

(3) Asymmetry of the waveform: a sharply rising ascending phase and a more slowly decaying descending phase, or vice versa.	(3) Asimmetria della forma d'onda: una fase ascendente fortemente pendente e una fase discendente più lenta, o viceversa.

(4) The transient is followed by an associated slow after-wave.	(4) il grafoelemento è seguito da una onda lenta.

(5) The background activity surrounding epileptiform discharges is disrupted by the presence of the epileptiform discharges.	(5) L'attività di fondo che circonda le scariche epilettiformi è perturbata per la presenza delle scariche epilettiformi.

(6) Distribution of the negative and positive potentials on the scalp suggests a source of the signal in the brain, corresponding to a radial, oblique or tangential orientation of the source (see dipole). This is best assessed by inspecting voltage maps constructed using common-average reference.	(6) La distribuzione dei potenziali negativi e positivi sullo scalpo suggerisce una sorgente del segnale, nonché il suo orientamento, radiale, obliquo o tangenziale (vedi dipolo). Ciò si valuta meglio ispezionando le mappe di voltaggio costruite usando una referenza media comune.
Synonyms: interictal epileptiform discharge, epileptiform activity.	Sinonimi: scarica epilettiforme intercritica, attività epilettiforme.

**Epoch**: EEG segment with a defined duration. Duration of epochs is determined arbitrarily but should be specified.	**Epoca:** segmento EEG con una durata definita. La durata delle epoche è determinata arbitrariamente ma dovrebbe essere specificata.

**Equipotential**: Applies to regions of the head or electrodes that are at the same potential at a given instant in time. Synonyms: isopotential line, isopotential.	**Equipotenziale:** si applica alle regioni della testa o agli elettrodi che sono allo stesso potenziale in un dato istante di tempo. Sinonimi: linea isopotenziale, isopotenziale.

**Event-related potential (ERP)**: Refer to long latency responses (>70 ms) associated with an event, such as a deviant stimulus (as in mismatch negativity, P3 or P300), anticipation of a response (as in Bereitschaftspotential), or anticipation of a stimulus demanding a response (as in contingent negative variation). Applied mainly to slow (on account of their lower frequency content) ‘endogenous’ evoked potentials elicited by controlled manipulation of the psychological context. Thought to reflect some aspect of higher sensory processing, and therefore sometimes referred to as “cognitive potentials”, such as: attention, expectancy, novelty detection, stimulus salience, target recognition, task relevance, information delivery, decision making, stimulus evaluation time, template matching, memory, and closure of cognitive epoch. (See evoked potential, contingent negative variation, mismatch negativity, P3 or P300).	**Potenziale evento-correlato (ERP, event-related potential):** si riferisce a risposte a lunga latenza (>70 ms) associate a un evento, come ad esempio uno stimolo deviante/discordante (come nella negatività da discordanza, P3 o P300), l'anticipazione di una risposta (come nel Bereitschaftspotential), o l'anticipazione di uno stimolo che richiede una risposta (come nella variazione negativa contingente). Applicato principalmente ai potenziali evocati “endogeni” lenti (a causa del loro contenuto di frequenza più basso) suscitati da una manipolazione controllata del significato attribuito agli stimoli presentati. Si ritiene che riflettano una elaborazione sensoriale superiore, e quindi a volte vengono chiamati “potenziali cognitivi”, come: attenzione, aspettativa, rilevamento di novità, salienza dello stimolo, riconoscimento del bersaglio, rilevanza del compito, consegna delle informazioni, processo decisionale, tempo di valutazione dello stimolo, corrispondenza del modello, memoria e chiusura dell'epoca cognitiva. (Vedi potenziale evocato, variazione negativa contingente, negatività da discordanza, P3 o P300).

**Evoked magnetic field**: Magnetic counterpart of EEG evoked potential. (See evoked potential, magnetoencephalography).	**Campo magnetico evocato:** controparte magnetica del potenziale evocato EEG. (Vedi potenziale evocato, magnetoencefalografia).

**Evoked potential (EP)**: Wave or complex elicited by and time-locked to a physiological or non-physiological stimulus or event, the timing of which can be reliably assessed. Comment: computer summation (averaging) techniques are especially suitable for detecting these and other event-related potentials from the surface of the head. See event-related potential.	**Potenziale evocato (EP, evoked potential):** onda o complesso evocato da e temporalmente legato a uno stimolo o evento fisiologico o non fisiologico, la cui latenza può essere valutata in modo affidabile. Commento: le tecniche di sommazione (media) computerizzate sono particolarmente adatte a rilevare questi e altri potenziali evento-correlati dalla superficie dello scalpo. Vedi potenziale evento-correlato.

**Exploring electrode**: An electrode that registers electrical potentials from excitable tissue of the nervous system, historically connected to the input terminal 1 of an EEG amplifier, against a reference electrode, connected to the input terminal 2. Synonym: active electrode (use discouraged as all recording electrodes may be considered ‘active’, including any reference electrode). (See reference electrode).	**Elettrodo esplorante:** un elettrodo che registra i potenziali elettrici dal tessuto eccitabile del sistema nervoso, storicamente collegato al terminale di ingresso 1 di un amplificatore EEG, verso un elettrodo di riferimento, collegato al terminale di ingresso 2. Sinonimo: elettrodo attivo (l'uso è sconsigliato in quanto tutti gli elettrodi di registrazione possono essere considerati “attivi”, compreso qualsiasi elettrodo di riferimento). (Vedi elettrodo di riferimento)

**Extracerebral potential**: Any potential that does not originate in the brain, generally referred to as an artifact in EEG. May arise from electrical interference external to the subject and recording system, the subject, the electrodes and their connections to the subject and the electroencephalograph, and the electroencephalograph itself (see artifact).	**Potenziale extracerebrale:** qualsiasi potenziale che non ha origine nel cervello, generalmente indicato come un artefatto sull'EEG. Può essere generato da interferenze elettriche esterne al soggetto e al sistema di registrazione, dal soggetto, dagli elettrodi e dalle loro connessioni al soggetto e all'elettroencefalografo, e dall'elettroencefalografo stesso (vedi artefatto).

**Extreme delta brush**: A particular pattern characterized by near continuous widespread rhythmic delta activity (1–3 c/s) with superimposed bursts of beta frequency activity (20–30 Hz) on top of each delta wave. Mostly symmetric and synchronous; do not vary with sleep-wake cycles or significantly with stimulation. The pattern has been described in autoimmune encephalitis associated with anti-N-Methyl D-Aspartate receptor antibodies. Named after a resemblance to the delta brush seen in preterm infants (see delta brush).	**Extreme delta brush:** un pattern particolare caratterizzato da un'attività delta ritmica diffusa quasi continua (1-3 Hz) con sovraimposizione, in ogni onda delta, di burst di attività di frequenza beta (20-30 Hz). È per lo più simmetrico e sincrono, non varia con i cicli sonno-veglia o significativamente con la stimolazione. Tale pattern è stato descritto nell'encefalite autoimmune associata ad anticorpi anti-recettori per N-Methyl D-Aspartato. Prende il nome da una somiglianza con il delta brush osservabile nei neonati pretermine (vedi delta brush).

**Far-field potential**: A potential generated in a deep neural structure and recorded by electrodes on the head at a distance from the generator, as a result of volume conduction and not mediated by neuronal activity. (See volume conduction, and for example brainstem auditory evoked potentials).	**Potenziale far-field (lett. campo lontano):** un potenziale generato in una struttura cerebrale profonda e registrato da elettrodi posti sul capo ad una certa distanza dal generatore, come risultante della conduzione di volume e non mediato quindi dall'attività neuronale. (Vedi conduzione di volume e come esempio potenziali evocati uditivi tronco-encefalici).

**Fast activity**: Activity of frequency higher than the alpha band, i.e. beta and gamma activity, and high frequency oscillations.	**Attività rapida:** attività di frequenza superiore alla banda alfa, cioè attività di banda beta e gamma, e oscillazioni ad alta frequenza.

**Fast alpha variant rhythm**: A normal variant. Characteristic rhythm at 14–20 Hz, detected most prominently over the posterior regions of the head. May alternate or be intermixed with alpha rhythm, of which it is usually a harmonic frequency. Blocked or attenuated by attention, especially visual, and mental effort.	**Variante rapida del ritmo alfa:** una variante normale. Ritmo a 14-20 Hz, evidente soprattutto nelle regioni posteriori del capo. Può alternarsi o inframmezzarsi al ritmo alfa, di cui è una frequenza armonica. È bloccato o attenuato dall'attenzione, soprattutto visiva, e dallo sforzo mentale.

**Fast ripples**: Part of the high frequency oscillation (HFO) bandwidth, usually defined as being in the range 250–1000 Hz (see high frequency oscillations).	**Fast ripples:** oscillazioni ad alta frequenza (HFO, high frequency oscillations) comprese tra 250 e 1000 Hz (vedi oscillazioni ad alta frequenza).

**Fast wave**: Wave with duration shorter than alpha waves, i.e. under 1/13 s.	**Onda rapida:** onda con durata più breve delle onde alfa, cioè sotto 1/13 s (superiore a 13 Hz).

**Focal**: Limited to a small area of the brain in one hemisphere (see regional, multifocal). Focal epileptic seizures are conceptualized as originating within networks limited to one hemisphere, and usually associated with an initially localized epileptiform EEG pattern (see epileptiform pattern).	**Focale:** limitato a una piccola area del cervello in un emisfero (vedi regionale, multifocale). Le crisi epilettiche focali si ritengono originare all'interno di network localizzati in un emisfero, e quindi di solito associate a un pattern EEG epilettiforme inizialmente localizzato (vedi pattern epilettiforme).

**Focus**: A limited region of the scalp, cerebral cortex, or depth of the brain displaying a given EEG activity, either normal or abnormal.	**Focus:** una regione limitata dello scalpo, della corteccia cerebrale o delle regioni cerebrali profonde che mostra una determinata attività EEG, normale o anormale.

**Foramen ovale electrode**: A multicontact electrode bundle inserted through the foramen ovale to lie in proximity to the mesial temporal cortex. Comment: used for presurgical assessment of epilepsy of suspected mesial temporal lobe origin. (See basal electrode).	**Elettrodo del forame ovale:** un elettrodo multicontatto inserito attraverso il forame ovale per posizionarsi in prossimità della corteccia temporale mesiale. Commento: è utilizzato per la valutazione prechirurgica dell'epilessia a presunta origine dal lobo temporale mesiale. (Vedi elettrodo basale).

**Fourteen and 6-Hz positive burst or spikes**: A normal variant. Burst of arch-shaped waves or spikes at 13–17 c/s and/or 5–7 c/s, but most commonly at 14 and/or 6 c/s, usually seen bilaterally over the posterior temporal and adjacent areas, typically during drowsiness and light sleep, with incidence peaking in adolescence. The sharp peaks of its component waves are positive with respect to other regions. Amplitude varies but is generally below 75 µV. Comments: (1) best demonstrated by referential recording using contralateral earlobe or common average reference electrodes. (2) This pattern has been termed “pseudo-epileptiform” (i.e. not associated with a liability to epileptic seizures). Synonym: ctenoids (use of term discouraged).	**Burst di onde arcuate o punte positive a 14 e/o 6 Hz:** una variante normale. Burst di onde arcuate o punte a 13-17 Hz e/o a 5-7 Hz, ma più comunemente a 14 e/o 6 Hz, di solito bilaterali sulle aree temporali posteriori e quelle adiacenti, tipicamente a comparsa durante la sonnolenza e il sonno leggero, con incidenza massima nell'adolescenza. La componente appuntita delle onde è a polarità positiva rispetto ad altre regioni. L'ampiezza varia ma è generalmente inferiore a 75 µV. Commenti: (1) pattern meglio evidente nelle registrazioni referenziali, utilizzando come referenza un elettrodo posto sul lobo auricolare controlaterale o la referenza media; (2) questo pattern è stato definito “pseudo-epilettiforme” (cioè non ritenuto associabile alle crisi epilettiche). Sinonimo: ctenoidi (uso del termine sconsigliato).

**Frequency**: Number of complete cycles of repetitive waves or complexes in 1 s. Measured in cycles per second (c/s) or Hertz (Hz). Comment: the term Hz seems appropriate when applied to sinusoidal waves such as alpha activity, but seems inappropriate when applied to complex waveforms such as spike-and-slow-waves, which may be more correctly quantified by c/s. This principle has been applied throughout this glossary.	**Frequenza:** numero di cicli completi di onde ripetitive o di complessi in 1 s. Misurato in cicli al secondo (c/s) o Hertz (Hz). Commento: il termine Hz sembra appropriato quando viene applicato a onde sinusoidali come l'attività alfa, ma sembra inappropriato quando viene applicato a forme d'onda più complesse come i complessi punta-onda lenta, che possono essere più correttamente quantificate in c/s. N.d.T. questo principio non è stato applicato nella traduzione del glossario, in cui si è scelto il termine univoco di Hz.

**Frequency response**: Characteristics of an amplifier showing the relative response to the activities of different frequencies with respect to the response of 10 Hz activity. The bandwidth of EEG channels is determined by the low and high frequency filters and the particular frequency response characteristics of the recording system.	**Risposta in frequenza:** caratteristica di un amplificatore che mostra la risposta relativa alle attività di frequenze diverse rispetto alla risposta dell'attività a 10 Hz. La larghezza di banda dei canali EEG è determinata dai filtri a bassa e alta frequenza e dalle particolari caratteristiche di risposta in frequenza del sistema di registrazione.

**Frequency response curve:** A graph depicting the relationships between output trace detection or amplifier output and input frequency, for a particular setting of low and high frequency filters.	**Curva di risposta in frequenza:** un grafico che rappresenta le relazioni tra la rilevazione della traccia di uscita o l'uscita dell'amplificatore e la frequenza di ingresso, per una particolare impostazione dei filtri a bassa e alta frequenza.

**Frequency spectrum**: The distribution of amplitude and phases of different frequency components against frequency. This is typically demonstrated by a Fourier transform of the EEG. Comment: In most applications the amplitude spectrum is presented only (for example in delta, theta, alpha, beta, gamma bands), and not phase information. (See power spectrum, quantitative EEG).	**Spettro di frequenza:** la distribuzione dell'ampiezza e delle fasi delle diverse componenti di frequenza del segnale. Questo è il risultato dell'applicazione della trasformata di Fourier al segnale EEG. Commento: nella maggior parte delle applicazioni viene presentato solo lo spettro di ampiezza (per esempio delle bande delta, theta, alfa, beta e gamma), e non le informazioni riguardo la fase. (Vedi spettro di potenza, EEG quantitativo).

**Frontal intermittent rhythmic delta activity (FIRDA**): Fairly regular, approximately sinusoidal or sawtooth waves, mostly occurring in bursts at 1.5–2.5 Hz synchronously over the frontal areas of both sides of the head (occasionally unilateral). Comment: most commonly associated with mild to moderate unspecified encephalopathy in responsive ambulant patients, often in association with cerebrovascular disease. Synonym: occasional frontally predominant brief 2/s GRDA.	**Attività delta ritmica intermittente frontale (FIRDA, frontal intermittent rhythmic delta activity):** onde abbastanza regolari, approssimativamente sinusoidali o a dente di sega, che si verificano per lo più in burst a 1,5-2,5 Hz in modo sincrono sulle aree frontali di entrambi i lati del capo (occasionalmente unilaterali). Commento: pattern comunemente associato con encefalopatia aspecifica di entità lieve-moderata in pazienti deambulanti e reattivi, spesso in associazione con patologia cerebrovascolare. Sinonimo: breve ed occasionale attività delta ritmica diffusa a 2 Hz, a predominanza frontale (GRDA, generalized rhythmic delta activity).

**Fronto-central theta**: A normal variant. Theta rhythm located in the midline, just anterior to the vertex, that occurs during psychological stress and cognitive tasks, particularly problem solving. It appears predominantly in young healthy adults (<30 years of age). The pattern is considered to be a normal response to cognitive tasks. Synonyms: frontal midline theta, Ciganek rhythm.	**Theta fronto-centrale:** una variante normale. Ritmo theta localizzato sulla linea mediana, appena anteriormente al vertice, che si osserva durante lo stress psicologico e i tasks cognitivi, in particolare durante il problem solving. Compare prevalentemente nei giovani adulti sani (<30 anni). Il pattern è considerato una risposta normale ai tasks cognitivi. Sinonimi: theta frontale della linea mediana, ritmo di Cigánek.

**Gain, voltage**: The ratio of output signal voltage, Vo, to input signal voltage, Vi, of an EEG channel. For example: Voltagegain=Vo/Vi=10V/10μV=1,000,000	**Guadagno, voltaggio:** il rapporto tra il voltaggio del segnale in uscita, (Vo) e il voltaggio del segnale di ingresso (Vi) di un canale EEG. Per esempio: Voltagegain=Vo/Vi=10V/10μV=1.000.000
The voltage gain (G) is often expressed in decibels (dB), a logarithmic ratio, defined as G=20log10(Vo/Vi)dB	Il guadagno di voltaggio (G) è spesso espresso in decibel (dB), un rapporto logaritmico, definito come G=20log10(Vo/Vi)dB
Examples: a voltage gain of 10 corresponds to G = 20 dB, of 1,000 to G = 60 dB, of 1,000,000 to G = 120 dB. Gain controls are used to attenuate and equalize the sensitivities of all channels (see sensitivity).	Esempi: un guadagno di voltaggio di 10 corrisponde a G = 20 dB, di 1.000 a G = 60 dB, di 1.000.000 a G = 120 dB. I controlli di guadagno sono utilizzati per attenuare ed equalizzare le sensibilità di tutti i canali (vedi sensibilità).

**Gamma band**: Frequency band from >30 to 80 Hz. Greek letter: γ. Comment: The graphic resolution of computer displays may limit the visual appreciation of higher frequencies. However, this does not justify limiting unduly the high frequency response of the EEG channels; for EEG waves include transients such as spikes and sharp waves with components at frequencies above 50 Hz.	**Banda gamma:** banda di frequenza da >30 a 80 Hz. Lettera greca: γ. Commento: La risoluzione grafica dei display dei computer può limitare la visualizzazione delle frequenze più alte. Tuttavia, questo non giustifica il taglio delle risposte ad alta frequenza dei canali EEG; infatti, le onde EEG comprendono transienti come punte e onde puntute con componenti a frequenze superiori a 50 Hz.

**Gamma rhythm or activity**: An EEG rhythm above >30–80 Hz (wave duration 12.5–33 ms). Comment: most commonly recorded with intracranial electrodes from actively engaged or driven neural networks.	**Ritmo o attività gamma:** un ritmo EEG superiore a >30-80 Hz (durata dell'onda 12,5-33 ms). Commento: più comunemente registrato con elettrodi intracranici inseriti in circuiti neuronali primariamente attivati oppure coinvolti secondariamente.

**Generalization**: Bilateral propagation of EEG activity from limited areas to all regions of the head (see generalized).	**Generalizzazione:** propagazione bilaterale dell'attività EEG, da aree limitate a tutte le regioni del capo (vedi generalizzato).

**Generalized**: Loosely: an EEG activity spread over all regions of the head, usually with a frontal, but rarely with an occipital, maximum (see diffuse). Strictly: bilateral EEG discharges appearing reasonably symmetrically and synchronously over homologous regions of the head (see symmetric and synchronous). For example generalized epileptic seizures are conceptualized as originating at some point within, and rapidly engaging (i.e. synchronizing), bilaterally distributed networks. Comment: “generalized” is still used as a term for describing seizure types and epilepsy syndromes, although no seizure pattern involves the whole brain simultaneously (see secondary bilateral synchrony).	**Generalizzato:** in senso generale: un'attività EEG diffusa su tutte le regioni dello scalpo, di solito con una prevalenza frontale, o più raramente occipitale (vedi diffuso). Più specificamente: scariche EEG bilaterali che appaiono sufficientemente simmetriche e sincrone su regioni omologhe del capo (vedi simmetrico e sincrono). Per esempio, le crisi epilettiche generalizzate sono concettualmente ad esordio in qualche punto all'interno di networks a distribuzione bilaterale, che vengono rapidamente coinvolti (cioè sincronizzati). Commento: “generalizzato” è ancora usato come termine per descrivere tipi di crisi e sindromi epilettiche, anche se nessun modello di crisi coinvolge l'intero cervello simultaneamente (vedi: sincronia bilaterale secondaria).

**Generalized paroxysmal fast activity (GPFA):** Bilateral synchronous bursts of spikes of 2–10 s duration, with frequency between 10 and 25 Hz (typically around 10 Hz) and maximum in the frontal regions that only occurs during sleep. GPFA is considered a feature of Lennox-Gastaut syndrome. Comment: when the bursts are longer than 5 s a tonic seizure is often recorded (this may be discrete, and only detected with surface EMG electrodes). Synonyms: bursts of fast rhythms, fast paroxysmal rhythms, runs of rapid spikes (use of terms discouraged).	**Attività parossistica rapida generalizzata (GPFA, generalized paroxysmal fast activity):** burst bilaterali e sincrone (di 2-10 s di durata) di punte con frequenza compresa tra 10 e 25 Hz (tipicamente intorno a 10 Hz), con prevalenza nelle regioni frontali, che si verificano soltanto durante il sonno. La GPFA è considerata una caratteristica tipica della sindrome di Lennox-Gastaut. Commento: quando le bursts durano più di 5 s si accompagnano spesso ad una crisi tonica (che può essere di modesta espressività clinica e rilevata solo con elettrodi EMG di superficie). Sinonimi: bursts di ritmi rapidi, ritmi rapidi parossistici, treni di punte rapide (l'uso di termini è scoraggiato).

**Generalized periodic discharges (GPDs)**: GPDs are generalized, synchronous, periodic or quasi-periodic complexes that occupy at least 50% of the record. They are high amplitude (typically >100 µV) and have duration of about 0.5 s, with an intervening background activity amplitude usually not more than 35 µV. The morphology of GPDs is variable and consists of sharp or spike and slow wave complexes, triphasic-like waves, and slow wave complexes. Repetition rate usually lies between 0.5 and 2.0 c/s. They occur most commonly in coma, usually after severe cerebral anoxia following cardiac arrest, in Creutzfeldt-Jacob disease, and with toxicity (for example baclofen or lithium). With anoxic insults, the periodicity typically ranges from 1.5 to 3.5 c/s. Most patients have a poor neurological prognosis or die, although not invariably. Synonym: generalized periodic epileptiform discharges (use of term discouraged). (See periodic discharges).	**Scariche periodiche generalizzate (GPDs, generalized periodic discharges):** sono rappresentate da complessi generalizzati, sincroni, periodici o quasi-periodici, che occupano almeno il 50% della registrazione. Hanno un'ampiezza elevata (tipicamente >100 µV) e una durata di circa 0,5 s, inscritte su un'attività di fondo con un'ampiezza che di solito non supera i 35 µV. La morfologia delle GPDs è variabile e consiste in complessi punta-onda-lenta o onda puntuta/onda lenta, oppure onde puntute trifasiche o infine complessi di onde lente. Il tasso di ripetizione di solito è compreso tra 0,5 e 2,0 Hz. Si osservano più comunemente nel coma, di solito come conseguenza di una grave anossia cerebrale per un arresto cardiaco, nella malattia di Creutzfeldt-Jacob e in seguito ad intossicazioni (per esempio da baclofen o litio). Negli insulti anossici, la periodicità varia tipicamente da 1,5 a 3,5 Hz. La maggior parte dei pazienti con questo pattern ha una prognosi infausta, compromettendo anche la sopravvivenza. Sinonimo: scariche epilettiformi periodiche generalizzate (l'uso del termine è sconsigliato). (Vedi: scariche periodiche).

**Generalized periodic epileptiform discharges (GPEDs)**: Use of term discouraged. See generalized periodic discharges (preferred term).	**Scariche epilettiformi periodiche generalizzate (GPEDs, generalized periodic epileptiform discharges):** uso del termine sconsigliato. Vedi scariche periodiche generalizzate (termine preferito).

**Graphoelement:** Any EEG pattern (transient, potential or rhythm) that is distinguished from the ongoing background activity, which may be physiological or pathological. It is characterized by its name, morphology, location, duration, frequency (when rhythmic), mode of appearance and the relationship to activating or modulating factors (for example hypnagogic hypersynchrony).	**Grafoelemento:** qualsiasi pattern EEG (transiente, potenziale o ritmo) che si distingue nettamente dall'attività di fondo, che può essere fisiologico o patologico. È caratterizzato dal nome specifico, dalla morfologia, dalla localizzazione, dalla durata, dalla frequenza (quando è ritmico), dalla modalità di comparsa e dal rapporto con fattori attivanti o modulanti (vedi ad esempio l'ipersincronia ipnagogica).

**Ground connection**: Conducting path between the subject, the electroencephalograph and earth.	**Collegamento a terra:** cavo di connessione fra il soggetto, l'elettroencefalografo e la terra.

**Ground projection**: Projection of an artifact, such as blink artifact, recorded from a ground electrode into an exploring electrode whose impedance is high.	**Proiezione a terra:** proiezione di un artefatto, come l'artefatto da ammiccamento, registrato da un elettrodo di terra verso un elettrodo esplorante la cui impedenza è alta.

**Harness, head**: A combination of straps fitted over the head to hold pad electrodes in position. Commercial EEG recording electrodes caps are an alternative.	**Cuffia:** una rete elastica applicata sul capo per tenere in posizione gli elettrodi a tampone. (Valida alternativa: cuffie precablate).

**Hertz (Hz)**: Unit of frequency. Synonym: cycles per second (c/s).	**Hertz (Hz):** unità di frequenza. Sinonimo: cicli al secondo (c/s).

**High frequency filter (or low pass filter**): A circuit that reduces the sensitivity of the EEG signals to relatively high frequencies (for example above 70 Hz). For each setting of the high frequency filter, this attenuation is expressed as percent reduction in signal amplitude at a given frequency, relative to frequencies unaffected by the filter, i.e. in the mid-frequency band of the signal. Synonym: low pass filter. Comment: at present high frequency filter designations and their significance are not yet standardized for all instruments of different manufactures. For instance, for a given instrument, a position of the high frequency filter control designated as 70 Hz may indicate a 30% (3 dB), or other stated percent, reduction in sensitivity at 70 Hz, compared to the sensitivity, for example, at 10 Hz.	**Filtro ad alta frequenza (o filtro passa-basso):** un circuito che riduce la sensibilità dei segnali EEG a frequenze relativamente alte (per esempio sopra i 70 Hz). Per ogni impostazione del filtro ad alta frequenza, questa attenuazione è espressa come riduzione percentuale dell'ampiezza del segnale a una data frequenza, rispetto alle frequenze non influenzate dal filtro, cioè nella banda delle medie frequenze del segnale. Sinonimo: filtro passa-basso. Commento: attualmente le denominazioni dei filtri ad alta frequenza e il loro significato non sono ancora standardizzati per tutti gli strumenti di diversi produttori. Per esempio, per un dato strumento, una posizione del controllo del filtro ad alta frequenza designato come 70 Hz può indicare una riduzione del 30% (3 dB), o altra percentuale dichiarata, della sensibilità a 70 Hz, rispetto alla sensibilità, per esempio, a 10 Hz.

**High frequency oscillations (HFOs)**: Transient bursts of EEG activity, spontaneous or evoked, with frequencies beyond 80 Hz. Divided into ripples (80–250 Hz) and fast ripples (250–500 Hz). (See ripples and fast ripples).	**Oscillazioni ad alta frequenza (HFOs, high frequency oscillations):** bursts transienti di attività EEG, spontanea o evocata, con frequenze oltre 80 Hz. Le HFOs si suddividono in ripples (80-250 Hz) e fast ripples (250-500 Hz). (Vedi ripples e fast ripples).

**High frequency response**: Sensitivity of an EEG channel to relatively high frequencies. Determined by the high frequency response of the amplifier and the high frequency filter used. Expressed as percent reduction in output trace deflection at certain specific high frequencies, relative to other frequencies in the mid-frequency band of the channel.	**Risposta ad alta frequenza:** sensibilità di un canale EEG a frequenze relativamente alte. È determinata dalla risposta ad alta frequenza dell'amplificatore e dal filtro ad alta frequenza utilizzato. È espressa come riduzione percentuale della deflessione della traccia in uscita a determinate specifiche alte frequenze, rispetto ad altre frequenze nella banda di frequenza media del canale.

**High pass filter**: Synonym: low frequency filter.	**Filtro passa alto:** sinonimo: filtro a bassa frequenza.

**Hypersynchrony**: When describing EEG patterns that are attributed to increased synchronization of neuronal activity (for example hypnagogic hypersynchrony).	**Ipersincronia:** quando si descrivono i pattern EEG che sono attribuiti a una maggiore sincronizzazione dell'attività neuronale (per esempio ipersincronia ipnagogica).

**Hyperventilation**: Deep and regular respiration performed for a period of several minutes. Used as an activation procedure. Synonym: overbreathing (see activation).	**Iperventilazione:** respirazione profonda e regolare eseguita per un periodo di alcuni minuti. Usata come procedura di attivazione. Sinonimo: iperpnea (vedi attivazione).

**Hypnagogic hypersynchrony:** A normal variant. Paroxysmal bursts of 3–5 c/s, high amplitude (75–350 µV) diffuse, but maximal fronto-central, sinusoidal activity occurring at the onset of sleep in normal infants and children, aged 3 months to 13 years (but typically 4–9 years).	**Ipersincronismo ipnagogico:** è una variante normale. Sequenze, talora con aspetto parossistico, di attività sinusoidale a 3-5 Hz, di ampiezza elevata (75-350 µV), diffusa, a massima espressione fronto-centrale, che si osserva all'inizio del sonno in bambini normali, dai 3 mesi ai 13 anni (ma tipicamente a 4-9 anni).

**Hypsarrhythmia**: Characteristic interictal EEG pattern typically, but not invariably, seen in infants with Infantile Spasms (West syndrome). Consists of diffuse very high amplitude (>300 µV) irregular slow waves interspersed with multiregional spikes and sharp waves over both hemispheres, usually with a highly disorganized and asynchronous appearance. It is most frequent during Non-REM sleep, followed by waking and arousal, and is absent or minimal during REM sleep. Variations include asymmetry, predominant single focus (within widespread abnormalities), episodes of attenuation or fragmentation, increased periodicity and preservation of interhemispheric synchrony (all termed ‘modified hypsarrhythmia’). Comment: the ictal pattern of spasms usually consists of one or more of following: diffuse high amplitude slow wave, low to medium amplitude fast activity, or electrodecrement (see low amplitude fast activity, electrodecrement).	**Ipsaritmia**: caratteristico pattern EEG intercritico tipicamente osservabile, ma non costantemente né esclusivo, nei bambini con spasmi infantili (sindrome di West). Consiste in diffuse onde lente irregolari, di ampiezza molto elevata (>300 µV), sulle quali si inscrivono punte ed onde puntute su entrambi gli emisferi, di solito in maniera altamente disorganizzata ed asincrona. Tale pattern è più frequente durante il sonno NREM, ma si osserva anche in veglia e al risveglio, mentre è assente o minimamente rappresentato durante il sonno REM (N.d.T.: e durante il grappolo di spasmi). Il pattern può presentarsi con alcune varianti caratterizzate da una o più delle seguenti caratteristiche: asimmetria, singola focalità predominante nell'ambito di anomalie diffuse, tratti di attenuazione o frammentazione, incremento della periodicità e conservazione della sincronia interemisferica (queste varianti prendono tutte il nome di 'ipsaritmia modificata').

	Commento: il pattern EEG critico degli spasmi di solito consiste in uno o più dei seguenti: onda lenta diffusa di ampiezza elevata, attività rapida di ampiezza medio-bassa o attività elettrodecrementale (vedi: attività rapida di bassa ampiezza, attività elettrodecrementale).

**Ictal EEG pattern:** See seizure pattern, EEG.	**Pattern EEG ictale:** vedi pattern critico, EEG.

**Impedance meter:** An instrument used to measure impedance (see electrode impedance).	**Impedenziometro: **uno strumento usato per misurare l'impedenza (vedi impedenza dell'elettrodo).

**Inactivity, record of electrocerebral**: See electrocerebral inactivity.	**Inattività, registrazione dell'inattività elettrocerebrale:** vedi inattività elettrocerebrale.

**Incidence:** Descriptor used to characterize how often a transient or isolated discharge is seen throughout the recording. Suggested ranges for describing this: ≥1/10 s are abundant, ≥1/min but less than 1/10 s are frequent, ≥1/h but less than 1/minute are occasional, and <1/h are rare. Comment: the equivalent descriptor for rhythmic EEG patterns is prevalence. Synonym: quantity. (See prevalence).	**Incidenza:** parametro che descrive la frequenza con cui un transiente o una scarica isolata si riscontra durante una registrazione. Intervalli suggeriti per descriverlo: ≥1/10 s sono abbondanti, ≥1/min ma meno di 1/10 s sono frequenti, ≥1/h ma meno di 1/min sono occasionali, e <1/h sono rari. Commento: il parametro equivalente per i pattern EEG ritmici è la prevalenza. Sinonimo: quantità. (Vedi prevalenza).

**Independent (temporally)**: Not dependent on some other EEG activity. Synonym: asynchronous.	**Indipendente (temporalmente):** non dipendente da qualche altra attività EEG. Sinonimo: asincrono.

**Index:** Percent of time an EEG activity is present in an EEG sample. For example: alpha index, spike wave index, daily pattern index.	**Indice:** percentuale di tempo in cui un'attività EEG è presente in un campione di un tracciato EEG. Per esempio: indice alfa, indice dei complessi punta-onda, indice giornaliero di un determinato pattern.

**Infraslow activity:** EEG activity with frequencies in the EEG below 0.1 Hz. Synonym: subdelta activity.	**Attività ultralenta:** attività EEG con frequenze lente inferiori a 0,1 Hz. Sinonimo: attività subdelta.

**In-phase signals:** Waves with no phase difference between them (see common mode signal – not a synonym).	**Segnali in fase:** onde senza differenza di fase tra loro (vedi segnale a modo comune - non è un sinonimo).

**Input:** The signal fed into an EEG amplifier (see input terminal 1; input terminal 2).	**Ingresso (input):** il segnale di ingresso in un amplificatore EEG (vedi terminale di ingresso 1; terminale di ingresso 2).

**Input circuit:** System consisting of the EEG electrodes and intervening tissues, the electrode leads, jack box, input cable, and electrode selectors.	**Circuito in ingresso:** sistema costituito dagli elettrodi EEG e dai tessuti circostanti, dalle derivazioni degli elettrodi, dalla scatola dei jack, dal cavo di ingresso e dai selettori degli elettrodi.

**Input impedance:** Impedance that exists between the two inputs of an EEG amplifier. Measured in ohms (generally mega-ohms, MΩ) with or without the additional specification of input shunt capacitance (measured in picofarads, pF). Comment: not a synonym of electrode impedance.	**Impedenza d'ingresso:** impedenza che esiste tra i due ingressi di un amplificatore EEG. Misurata in ohms (generalmente mega-Ohm, MΩ) con o senza la specifica aggiuntiva della capacità di shunt di ingresso (misurata in picofarad, pF). Commento: non è un sinonimo di impedenza dell'elettrodo.

**Input terminal 1:** The input terminal of the differential EEG amplifier at which negativity, relative to the other input terminal, produces an upward trace deflection (see polarity convention). Synonyms: “grid 1” (G1), black lead (use of terms discouraged). Comment: customarily the connection of an electrode to the input terminal 1 of the EEG amplifier is represented in diagrams as a solid line.	**Terminale d'ingresso 1:** il terminale d'ingresso dell'amplificatore EEG differenziale al quale la negatività, rispetto all'altro terminale d'ingresso, produce una deflessione della traccia verso l'alto (vedi convenzione di polarità). Sinonimi: “griglia 1” (G1), cavo nero (l'uso dei termini è sconsigliato). Commento: di solito il collegamento di un elettrodo al terminale d'ingresso 1 dell'amplificatore EEG è rappresentato nei diagrammi come una linea continua.

**Input terminal 2:** The input terminal of the differential EEG amplifier at which negativity, relative to the other input terminal, produces a downward trace deflection (see polarity convention). Synonyms: “grid 2” (G2), white lead (use of terms discouraged). Comment: the connection of an electrode to the input terminal 2 of the EEG amplifier is represented in diagrams as a dotted or dashed line.	**Terminale d'ingresso 2:** il terminale d'ingresso dell'amplificatore EEG differenziale al quale la negatività, rispetto all'altro terminale d'ingresso, produce una deflessione della traccia verso il basso (vedi convenzione di polarità). Sinonimi: “griglia 2” (G2), “cavo bianco” (l'uso dei termini è sconsigliato). Commento: il collegamento di un elettrodo al terminale d'ingresso 2 dell'amplificatore EEG è rappresentato nei diagrammi come una linea tratteggiata o punteggiata.

**Input voltage:** Potential difference between the two input terminals of a differential EEG amplifier (see differential amplifier).	**Tensione d'ingresso:** differenza di potenziale tra i due terminali d'ingresso di un amplificatore EEG differenziale (vedi amplificatore differenziale).

**Inter-electrode distance:** Spacing between pairs of electrodes. Comment: distances between adjacent electrodes placed according to the standard 10–20 system or more closely spaced electrodes are frequently referred to as short or small inter-electrode distances (i.e. 10–10 system). Larger distances (such as double) between standard electrode placements are often termed widely spaced, long or large inter-electrode distances.	**Distanza inter-elettrodo:** distanza tra coppie di elettrodi. Commento: le distanze tra elettrodi adiacenti posizionati secondo il sistema standard 10-20 o gli elettrodi più ravvicinati sono spesso indicate come distanze inter-elettrodiche brevi (per esempio secondo il sistema 10-10). Distanze maggiori (fino al doppio) tra elettrodi posizionati secondo il sistema standard sono spesso definite distanze inter-elettrodiche ampie o molto ampie.

**Interhemispheric derivation:** Recording between a pair of electrodes located on opposite sides of the head (for example F3-F4).	**Derivazione interemisferica:** registrazione tra una coppia di elettrodi situati su lati opposti del capo (per esempio F3-F4).

**Intermittent photic stimulation**: Delivery of intermittent flashes of light to the eyes of a subject. Used as EEG activation procedure. Synonym: photic stimulation (PS).	**Stimolazione luminosa intermittente:** erogazione di flash luminosi intermittenti davanti agli occhi di un soggetto. Utilizzata come prova di attivazione EEG. Sinonimo: stimolazione luminosa (PS, photic stimulation).

**Intermittent slow activity:** Slow EEG activity that occurs intermittently and is not caused by drowsiness (usually >100 µV). Intermittent slow activity varies by more than 50% or regresses completely between times of its appearance, and can be polymorphic, arrhythmic or rhythmical (see continuous slow activity).	**Attività lenta intermittente:** attività EEG lenta che si manifesta in modo intermittente e non è causata dalla sonnolenza (di solito > 100 µV). L'attività lenta intermittente varia di più del 50% oppure può regredire completamente tra una comparsa e l'altra e può essere polimorfa, aritmica o ritmica (vedi attività lenta continua).

**Intracerebral depth electroencephalogram**: See: depth electroencephalogram.	**Elettroencefalogramma intracerebrale di profondità:** vedi: elettroencefalogramma di profondità.

**Intracerebral electrode:** Various conducting devices for recording EEG from the surface or within the substance of the brain. Examples include epicortical/subdural, epidural, foramen ovale, and stereotactic [stereotaxic] implanted depth electrodes. Synonym: depth electrode.	**Elettrodo intracerebrale:** dispositivi di vario tipo idonei per la registrazione dell'EEG dalla superficie o all'interno delle strutture cerebrali. Esempi: elettrodi di profondità impiantati con modalità stereotassica. Sinonimo: elettrodo di profondità.

**Irregular:** Applies to EEG waves and complexes of inconstant period and/or uneven contour or morphology.	**Irregolare:** si applica a onde e complessi EEG con un incostante periodismo e/o morfologia irregolare.

**Isoelectric:** (1) The record obtained from a pair of equipotential electrodes (see equipotential). (2) Use of term discouraged when describing record of electrocerebral inactivity (see electrocerebral inactivity).	**Isoelettrico:** (1) la registrazione ottenuta da una coppia di elettrodi equipotenziali (vedi equipotenziale); (2) l'uso del termine è sconsigliato quando si descrivono registrazioni di inattività elettrocerebrale (vedi inattività elettrocerebrale).

**Isolated:** Occurring singly.	**Isolato:** che si verifica singolarmente.

**Isopotential:** See synonym equipotential.	**Isopotenziale:** sinonimo di equipotenziale (vedi).

**K complex:** A normal graphoelement. A well delineated negative sharp wave followed by a positive component standing out from the background EEG, with total duration ≥0.5 s, usually maximal in amplitude when recorded from fronto-central derivations and often associated with a sleep spindle. (See vertex sharp transient or vertex sharp wave).	**Complesso K:** grafoelemento normale. Un'onda aguzza negativa ben definita seguita da una componente positiva che si distingue dall'attività di fondo, con durata totale ≥ 0,5 s, solitamente di ampiezza massima quando registrata dalle derivazioni fronto-centrali e spesso associata a un fuso del sonno. (Vedi punte al vertice).

**Lambda wave**: A normal graphoelement. Diphasic sharp transient occurring over the occipital regions of the head of awake subjects during visual exploration. The main component is positive relative to other areas. Time-locked to saccadic eye movements. Amplitude varies but is generally below 50 µV. Greek letter: λ (note morphology resembling the Greek capital letter lambda).	**Onda lambda:** grafoelemento normale. Onda a morfologia bifasica che compare sulle regioni occipitali nei soggetti in veglia durante l'esplorazione visiva. La componente principale è positiva. È temporalmente correlata ai movimenti oculari saccadici. L'ampiezza varia ma è generalmente inferiore a 50 µV. Lettera greca: λ (la morfologia dell’onda è simile alla lettera greca lambda).

**Laplacian montage:** Montage that consists of a mathematical transformation involving the second spatial derivative; the Laplacian source of the potential may be approximated by using the weighted average of all the neighbouring electrodes as a reference for each site or electrode. This montage may be used for localization of focal abnormalities on digital EEG (see common average reference).	**Montaggio laplaciano:** montaggio che è il risultato di una trasformazione matematica relativa alla seconda derivata spaziale; la sorgente laplaciana del potenziale può essere approssimata usando la media ponderata di tutti gli elettrodi circostanti come referenza per ogni sito o elettrodo. Questo montaggio può essere utilizzato per la localizzazione di anomalie focali sull'EEG digitale (vedi referenza comune media).

**Lateralized:** Independently involving the right and/or left side of the head (or body) (see unilateral).	**Lateralizzato:** coinvolgimento indipendente del lato destro e/o sinistro del capo (o del corpo) (vedi unilaterale).

**Lateralized periodic discharges (LPDs):** LPDs are unilateral surface negative discharges of spike, sharp or sharp slow-wave polyphasic morphology, usually lasting from 100 to 300 ms that typically recur at quasiperiodic intervals of up to 3/s. The incidence of clinical or electrographic seizures associated with LPDs is high, ranging from 50 to 100%, but there is debate as to whether they represent seizures proper. When contralateral motor movements are time-locked to LPDs they are considered to represent seizure patterns. Most LPDs are ephemeral phenomena occurring with both acute focal destructive lesions (for example cerebral infarcts, tumors or herpes simplex encephalitis) and more subacute/chronic pathologies (for example epilepsy and vascular compromise). Synonym: periodic lateralised epileptiform discharges (use of term discouraged). (See discharge, periodic discharges).	**Scariche periodiche lateralizzate (LPDs, lateralized periodic discharges):** sono scariche unilaterali a polarità negativa, caratterizzate da punte, onde puntute o complessi onda puntuta-onda-lenta a morfologia polifasica, della durata da 100 a 300 ms, che tipicamente ricorrono a intervalli quasi periodici fino a 3 Hz. L'incidenza di crisi cliniche o elettriche associate alle LPDs è elevata, dal 50 al 100%, ma è controverso se le LPDs abbiano un significato critico di per sé. Tuttavia, nel caso in cui siano temporalmente correlate a dei movimenti involontari controlaterali, esse rappresentano un pattern critico. La maggior parte delle LPDs sono manifestazioni transitorie in correlazione con lesioni cerebrali focali acute (per esempio infarti cerebrali, tumori o encefalite da herpes simplex) o con patologie subacute/croniche (per esempio epilessia da encefalopatia vascolare). Sinonimo: scariche epilettiformi periodiche lateralizzate (l'uso del termine è sconsigliato). (Vedi scarica, scariche periodiche).

**Lead:** Strictly: wire connecting an electrode to the electroencephalograph. Loosely: synonym of electrode, its wire and connector.	**Cavo:** in senso stretto, filo che collega un elettrodo all'elettroencefalografo. In senso lato, sinonimo di elettrodo, del suo filo e connettore.

**Light sleep:** Non-REM (NREM) sleep stages N1 and N2, which are characterized by sinusoidal eye movements, low amplitude mixed frequency EEG activity, vertex sharp waves, K complexes and sleep spindles. (See deep sleep).	**Sonno leggero:** è costituito dallo stadio N1 e N2 del sonno non REM (NREM), caratterizzato da movimenti oculari sinusoidali, attività EEG di bassa ampiezza e frequenza mista, punte al vertice, complessi K e fusi del sonno (vedi sonno profondo).

**Linkage:** The connection of a pair of electrodes to the two respective input terminals of a differential EEG amplifier (see derivation).	**Collegamento:** connessione di una coppia di elettrodi ai due rispettivi terminali di ingresso di un amplificatore EEG differenziale (vedi derivazione).

**Longitudinal bipolar montage**: A montage consisting of contiguous channels of electrode pairs along longitudinal, mainly antero-posterior, arrays (for example, Fp1-F3, F3-C3, C3-P3, P3-O1 etc). Synonym: “double-banana” montage.	**Montaggio longitudinale bipolare:** montaggio costituito da coppie di elettrodi di canali contigui a disposizione longitudinale, tipicamente in senso antero-posteriore (per esempio, Fp1-F3, F3-C3, C3-P3, P3-O1 ecc.) Sinonimo: montaggio “doppia banana”.

**Low frequency filter (high pass filter)**: A circuit that reduces the sensitivity of the EEG signal to relatively low frequencies (for example below 0.5 Hz). For each position of the low frequency filter control, this attenuation is expressed as percent reduction of the signal at a given stated frequency, relative to frequencies unaffected by the filter, i.e. in the mid-frequency band of the channel. Comment: at present low frequency filter designations and their significance are not yet standardized for instruments of different manufacturers. For instance, in a given instrument a low frequency filter setting designated 1 Hz may indicate a 30% (3 dB), or other stated percent, reduction in sensitivity at 1 Hz, compared to the sensitivity for example at 10 Hz. The same position of the low frequency filter setting may also be designated by the time constant. Synonym: high pass filter.	**Filtro basse frequenze (filtro passa alto):** sistema che riduce la sensibilità del segnale EEG alle frequenze relativamente basse (per esempio inferiori a 0,5 Hz). Il filtro è espresso come riduzione percentuale del segnale a una data frequenza dichiarata, rispetto alle frequenze non influenzate dal filtro, cioè nella banda delle frequenze medie del canale. Commento: attualmente le denominazioni dei filtri a bassa frequenza ed il loro significato non sono ancora standardizzati per strumenti di diversi produttori. Per esempio, in un dato strumento l'impostazione del filtro a 1 Hz può indicare una riduzione del 30% (3 dB) della sensibilità a 1 Hz, o un'altra percentuale dichiarata, rispetto alla sensibilità per esempio a 10 Hz. La stessa impostazione del filtro a bassa frequenza può anche essere designata dalla costante di tempo. Sinonimo: filtro passa alto.

**Low frequency response:** Sensitivity of an EEG channel to relatively low frequencies. Determined by the low frequency response of the amplifier and by the low frequency filter (time constant) used. Expressed as percent reduction in output trace deflection at certain stated low frequencies, relative to other frequencies in the mid-frequency band of the channel (see low frequency filter, time constant).	**Risposta alle basse frequenze:** sensibilità di un canale EEG a frequenze relativamente basse; determinata dalla risposta alle basse frequenze dell'amplificatore e dal filtro utilizzato per le basse frequenze (costante di tempo); espressa come riduzione percentuale della deflessione della traccia di output a specifiche basse frequenze, rispetto ad altre frequenze nel range delle medie frequenze di un canale (vedi filtro basse frequenze, costante di tempo).

**Low pass filter:** Synonym: high frequency filter.	**Filtro passa-basso:** sinonimo: filtro ad alta frequenza.

**Low voltage EEG:** A normal variant. Waking record characterized by activity of amplitude not greater than 20 µV over all head regions. With appropriate instrumental sensitivities this activity can be shown to be composed primarily of beta, theta and, to a lesser degree, delta waves, with or without alpha activity over the posterior areas. Comments: (1) low voltage EEGs are susceptible to change under the influence of certain physiological stimuli, sleep, pharmacological agents and pathological processes. (2) They should be clearly distinguished from tracings of electrocerebral inactivity, suppression and low voltage fast activity (see electrocerebral inactivity, suppression and low voltage fast activity).	**EEG di basso voltaggio:** una variante normale. Registrazione in veglia caratterizzata da un'attività di ampiezza non superiore a 20 µV su tutte le derivazioni. Con la sensibilità appropriata, si può dimostrare che questa attività è composta principalmente dalla banda beta, theta e, in minor misura, delta, con o senza attività alfa sulle derivazioni posteriori. Commenti: (1) le registrazioni EEG di basso voltaggio sono maggiormente suscettibili di modificazione sotto l'influenza di alcuni stimoli fisiologici, sonno, agenti farmacologici e processi patologici; (2) devono essere chiaramente distinti dai tracciati caratterizzati da inattività elettrocerebrale, soppressione e attività rapida di basso voltaggio (vedi inattività elettrocerebrale, soppressione e attività rapida di basso voltaggio).

**Low voltage fast activity:** Refers to fast activity (beta rhythm and above), often recruiting, which can be recorded at the onset of an ictal discharge, particularly in intra-cranial depth EEG recording of a seizure.	**Attività rapida di basso voltaggio:** si riferisce all'attività rapida (ritmo beta o superiore), spesso reclutante, che può essere registrata all'inizio di una scarica critica, in particolare nelle registrazioni EEG mediante elettrodi di profondità intracranici di una crisi.

**Magnetoencephalography (MEG)**: Recording of magnetic fields generated from the cortical neurons.	**Magnetoencefalografia (MEG):** registrazione dei campi magnetici generati dai neuroni corticali.

**Map, voltage**: Topographical display of the voltage distribution on the scalp, using equipotential lines and color-codes to express the steps of gradient changes between the peak negativity and the peak positivity. The voltage difference between peak negativity and peak positivity is 100%, and the fall-off of the potential is shown in arbitrary steps of, for example, 10% of the maximum amplitude. Usually blue color symbolizes negativity, and red color positivity. Inspecting voltage maps allows estimation of the location and orientation of the source. Comment: it is recommended to calculate voltage maps using common average reference (that include all electrodes on the scalp and preferably the inferior temporal electrode chain too). (See Quantitative EEG). Synonyms: diagram of equipotential lines, isopotential map or amplitude map.	**Mappa, voltaggio:** visualizzazione topografica della distribuzione del voltaggio sullo scalpo, mediante linee di equipotenziale e un codice colore che esprime i cambiamenti di gradiente nel passaggio da un picco negativo ad uno positivo. La differenza di voltaggio tra un picco negativo ed uno positivo è il 100%, mentre la modificazione del potenziale è mostrata, per esempio, in una scala arbitraria con step del 10% dell'ampiezza massima. Di solito il colore blu simboleggia la negatività, e il colore rosso la positività. La valutazione delle mappe permette la stima della posizione e dell'orientamento della sorgente. Commento: si raccomanda di calcolare le mappe di voltaggio usando una referenza media comune (che include tutti gli elettrodi sullo scalpo e preferibilmente anche la serie di elettrodi sulle regioni temporali inferiori. (Vedi EEG quantitativo). Sinonimi: diagramma con le linee equipotenziali, mappa isopotenziale o mappa di ampiezza.

**Mismatch negativity (MMN):** Is an automatic (i.e. attention independent) event-related response to physically deviant auditory stimuli occurring among frequent (standard) stimuli (e.g. tones or phonetic stimuli). MMN is a surface negative potential with an onset latency of about 130 ms and lasting 250–300 ms, with maximal amplitude over the fronto-central region. (See event-related potential).	**Mismatch negativity (MMN):** è una risposta evento-correlata automatica (cioè indipendente dall'attenzione) a stimoli uditivi fisicamente devianti che si verificano tra gli stimoli frequenti (standard) (per esempio toni o stimoli fonetici). La MMN è un potenziale negativo di superficie con una latenza di circa 130 ms e una durata di 250-300 ms, con ampiezza massima sulle regioni fronto-centrali. (Vedi potenziale evento-correlato).

**Monorhythmic delta activity:** A normal graphoelement in preterm infants (24–34 weeks of post menstrual age). Characterized by relatively stereotyped delta activity (up to 200 µV) predominantly over the posterior regions (occipital, temporal and central).	**Attività delta monoritmica:** grafoelemento normale nei neonati pretermine (24-34 settimane di età gestazionale). È caratterizzata da un'attività delta relativamente stereotipata (fino a 200 µV) prevalente sulle regioni posteriori (occipitale, temporale e centrale).

**Montage:** The arrangement or array of channels on the EEG machine display, defined by the exploring and reference electrodes (for example see bipolar and referential montages).	**Montaggio:** disposizione dei canali sul monitor del sistema EEG, costituito dagli elettrodi registranti e di riferimento (per esempio vedi montaggi bipolari e referenziali).

**Morphology:** Refers to the form of EEG waves (i.e. their shape and physical characteristics).	**Morfologia:** si riferisce alla forma delle onde EEG (cioè forma e caratteristiche fisiche).

**Motor evoked potential (MEP)**: Evoked potential recorded from muscle following direct stimulation of the exposed motor cortex, or transcranial stimulation of the motor cortex, either magnetically or electrically.	**Potenziale evocato motorio (PEM):** potenziale registrato da un muscolo ed evocato dalla stimolazione diretta della corteccia motoria esposta, o dalla stimolazione transcranica, sia magnetica che elettrica, della corteccia motoria.

**Mu rhythm:** Rhythm at 7–11 Hz, composed of arch-shaped waves occurring over the central or centro-parietal regions of the scalp during wakefulness. Amplitude varies but is mostly below 50 µV. Blocked or attenuated most clearly by contralateral movement, thought of movement, readiness to move or tactile stimulation. Greek letter: μ. Synonyms: rhythm rolandique en arceau, comb rhythm (use of terms discouraged).	**Ritmo mu:** ritmo a 7-11 Hz, composto da onde a forma di arco che si registra sulle derivazioni centrali o centro-parietali dello scalpo e in veglia. L'ampiezza varia ma è per lo più inferiore a 50 µV. È bloccato o almeno attenuato per lo più dal movimento controlaterale, dall'ideazione del movimento, dalla preparazione al movimento o dalla stimolazione tattile. Lettera greca: μ. Sinonimi: ritmo rolandico en arceau, ritmo a pettine (l'uso dei termini è sconsigliato).

**Multifocal:** Three or more spatially separated independent foci (see focal).	**Multifocale:** presenza di tre o più focolai indipendenti e spazialmente separati (vedi focale).

**Multiple spike-and-slow-wave complex:** Use of term discouraged. An epileptiform graphoelement consisting of two or more spikes associated with one or more slow waves (see epileptiform pattern). Synonym: polyspike and-slow-wave complex (preferred term).	**Complessi di punta e onda lenta multipli:** l'uso del termine è sconsigliato. Grafoelemento epilettiforme che consiste in due o più punte associate a una o più onde lente (vedi anomalia epilettiforme). Sinonimo: complesso di polipunte e onde lente (termine preferito).

**Multiple spike complex:** Use of term discouraged. A sequence of two or more spikes. Synonym: polyspike complex (preferred term).	**Complesso di punte multiple:** l'uso del termine è sconsigliato. Una sequenza di due o più punte. Sinonimo: complesso di polipunte (termine consigliato).

**Multiregional:** Three or more lobar foci (see regional).	**Multiregionale:** tre o più focolai lobari (vedi regionale).

**Nasopharyngeal electrode:** Rod electrode introduced through the nose and placed against the nasopharyngeal wall with its tip lying near the body of the sphenoid bone. (See basal electrode).	**Elettrodo nasofaringeo:** elettrodo introdotto attraverso le cavità nasali e posizionato contro la parete nasofaringea con la sua estremità vicino al corpo dell'osso sfenoide (vedi elettrodo basale).

**Needle electrode:** Small needle inserted into the subdermal layer of the scalp.	**Elettrodo ad ago:** piccolo ago inserito nello strato subdermico dello scalpo.

**Noise, EEG channel:** Small fluctuating output of an EEG channel recorded when high sensitivities are used, even if there is no input signal. Measured in microvolts (µV), referenced to the input.	**Rumore, canale EEG:** segnale in uscita (output) da un canale EEG, di modesta entità e fluttuante, registrato quando si usano alte sensibilità, anche se non c'è alcun segnale in ingresso (input). Misurato in microvolt (µV), riferito all'input.

**Non-cephalic reference:** Reference electrode that is placed on body parts other than the head (for example sternospinal reference).	**Referenza non-cefalica:** elettrodo di riferimento che viene posto su parti diverse del corpo (per esempio riferimento sternospinale).

**Non-REM sleep (NREM)**: Term summarizing all sleep stages except REM sleep (see REM sleep).	**Sonno non REM (NREM):** termine che riassume tutte le fasi del sonno tranne il sonno REM (vedi sonno REM).

**Notch filter:** A filter that selectively attenuates a very narrow frequency band, thus producing a sharp notch in the frequency response of an EEG signal. Commonly applied to attenuate electrical noise from mains interference (the frequency of which differs between countries, 50 or 60 Hz), which may occur under unfavorable technical conditions.	**Filtro notch:** filtro che attenua selettivamente un intervallo di frequenza molto ristretto producendo così un taglio netto di quella frequenza nel segnale EEG. Comunemente applicato per attenuare il rumore elettrico da interferenze di rete (la cui frequenza varia tra Paesi, 50 o 60 Hz), che può verificarsi in condizioni tecniche sfavorevoli.

**Nyquist theorem:** Accurate digital representation of an EEG signal requires that the sampling rate is at least twice the highest frequency of the signal, i.e. a frequency component of 30 Hz requires at least a sampling rate of 60 Hz. Comment: sampling at twice the Nyquist frequency only ensures an accurate representation of frequency content. Tolerable reproduction of waveforms requires at least a sampling rate 5 times above the fastest frequency components present.	**Teorema di Nyquist:** una registrazione digitale accurata di un segnale EEG richiede che la frequenza di campionamento sia almeno il doppio della frequenza più alta contenuta nel segnale, per es. la registrazione di una frequenza pari a 30 Hz richiede almeno una frequenza di campionamento di 60 Hz. Commento: il campionamento al doppio della frequenza di Nyquist assicura solo una registrazione accurata delle frequenze contenute in quel segnale. Una riproduzione accettabile della forma di un'onda richiede invece una frequenza di campionamento 5 volte superiore alle frequenze più alte.

**Occipital intermittent rhythmic delta activity (OIRDA)**: Fairly regular or approximately sinusoidal waves, mostly occurring in bursts at 2–3 Hz over the occipital areas of one or both sides of the head. Frequently blocked or attenuated by eye opening. An abnormal pattern seen in children’s EEGs more frequently than adults, often but not exclusively in association with genetic generalized epilepsies.	**Attività delta ritmica intermittente occipitale (OIRDA, occipital intermittent rhythmic delta activity):** attività abbastanza regolare e simil-sinusoidale, tipicamente in sequenze a circa 2-3 Hz, che compare sulle derivazioni occipitali, sia mono che bilaterale; spesso bloccata o attenuata dall'apertura degli occhi. Si tratta di un pattern EEG anormale, più comune nei bambini che negli adulti, che spesso, ma non esclusivamente, si associa alle epilessie generalizzate genetiche.

**Ohmmeter**: An instrument used to measure resistance (see electrode resistance).	**Ohmmetro:** uno strumento usato per misurare la resistenza elettrica (vedi resistenza degli elettrodi).

**Ordinate period:** Time in milliseconds (ms) elapsing between two successive sampling points in digital EEG. (See bin width).	**Periodo di campionamento:** tempo in millisecondi (ms) che intercorre tra due punti di campionamento successivi nell'EEG digitale. (Vedi dimensione del bin).

**Organization:** Degree to which the posterior dominant rhythm (PDR) conforms to certain characteristics displayed by a majority of subjects in the same age group, without personal or family history of neurologic and psychiatric diseases, or other illnesses that might be associated with dysfunction of the brain. Comments: the organization of PDR progresses from birth to adulthood.	**Organizzazione:** valutazione del grado di conformità del ritmo posteriore dominante (PDR, posterior dominant rhythm) a certe caratteristiche comuni alla maggioranza dei soggetti della stessa fascia d'età, senza storia personale o familiare di malattie neurologiche e psichiatriche, o altre malattie che potrebbero essere associate a disfunzioni cerebrali. Commenti: l'organizzazione del PDR si modifica nel corso della vita, dalla nascita all'età adulta.

**Out-of-phase signals:** Two waves of opposite phases (see differential signal; phase reversal - not a synonym).	**Segnale fuori fase:** due onde caratterizzate da fase opposta (vedi segnale differenziale; il termine “inversione di fase” non è un sinonimo).

**Output voltage:** The voltage across the trace display of an EEG channel.	**Voltaggio in uscita:** voltaggio rappresentato nella traccia di un canale EEG.

**Overbreathing:** Synonym: hyperventilation.	**Iperpnea:** sinonimo: iperventilazione.

**Overload:** Condition caused by applying voltage differences which are larger than the channel is designed for or set to handle by the input terminals of an EEG amplifier. Displays clipping of EEG waves and/or blocking of the amplifier depending on its magnitude (see clipping, blocking).	**Sovraccarico:** condizione causata dall'applicazione di una differenza di potenziale superiore a quella per cui il canale dell'amplificatore EEG è progettato o impostato. La visualizzazione del tracciato EEG viene interrotta e/o l'amplificatore si blocca, a seconda dell'ordine di grandezza del sovraccarico (vedi clipping, blocco).

**P3 or P300:** Is an event-related potential response usually elicited using the oddball paradigm, in which low-probability target stimuli are mixed with high-probability non-target (or standard) stimuli. P3 is a surface positive potential with an onset latency of about 250 to 500 ms and maximal amplitude over the centro-parietal region, with two subcomponents denoted P3a and P3b. Synonym: late positive component (LPC). (See event-related potential).	**P3 o P300:** è un potenziale evento-correlato, elicitato da stimoli rilevanti rari (paradigma oddball), caratterizzati da stimoli target a bassa probabilità mescolati a stimoli non target ad alta probabilità (o standard). P3 è un potenziale positivo con una latenza a circa 250-500 ms e un'ampiezza massima sulle regioni centro-parietali, composto da due sottocomponenti denominate P3a e P3b. Sinonimo: componente positiva tardiva (LPC, late positive component). (Vedi potenziale evento-correlato).

**Pad electrode:** Metal electrode covered with a cotton or felt and gauze pad, held in position by a head cap or harness.	**Elettrodo a tampone:** elettrodo metallico coperto da un cuscinetto di cotone, feltro o garza, tenuto in posizione da una cuffia.

**Paper speed:** Velocity of movement of paper through an analogue EEG machine. Expressed in centimeters per second (cm/s) or millimeters per second (mm/s). Synonym: time base (in digital EEG).	**Velocità di scorrimento della carta:** velocità di avanzamento della carta attraverso un elettroencefalografo analogico. Espressa in centimetri al secondo (cm/s) o millimetri al secondo (mm/s). Nell'EEG digitale si fa riferimento alla base dei tempi.

**Paroxysm:** Graphoelement phenomenon with sudden onset, rapid attainment of a maximum, and abrupt termination; distinguished from background activity. Comment: commonly used to refer to epileptiform and seizure patterns (see epileptiform pattern and seizure pattern).	**Parossismo:** comparsa improvvisa di un grafoelemento che rapidamente raggiunge il massimo, cessa bruscamente e si distingue dall'attività di fondo. Commento: tipicamente si riferisce a un pattern epilettiforme e a un pattern critico (vedi).

**Paroxysmal fast:** Fast frequencies in the beta range or above occurring in trains (see paroxysm, low voltage fast activity).	**Attività rapida parossistica:** treni di attività di frequenza beta o superiore (vedi parossismo, attività rapida di basso voltaggio).

**Pattern:** Any characteristic regular or repetitive EEG activity of approximately constant period (see regular and rhythmic).	**Pattern:** qualsiasi attività EEG regolare o ripetitiva con un periodo approssimativamente costante (vedi regolare e ritmico).

**Peak**: Point of maximum amplitude of a wave.	**Picco:** punto di massima ampiezza di un'onda.

**Period:** Duration of complete cycle of individual graphoelement in a sequence of regularly repeated EEG waves or complexes. Comment: the period of the graphoelement of an EEG rhythm is the reciprocal of the frequency of the rhythm. (For example, the duration of a spike-and-slow-wave complex in 3 c/s spike-and-slow-waves is 1/3 = 0.333).	**Periodo:** durata del ciclo completo di un singolo grafoelemento in una sequenza di onde o complessi EEG che si ripetono con regolarità. Commento: il periodo di un grafoelemento in un ritmo EEG è il reciproco della frequenza del ritmo (per esempio, la durata di un complesso punta-onda-lenta in una sequenza a 3 Hz è 1/3 = 0,333).

**Periodic:** Applies to: (1) EEG waves or complexes occurring in a sequence at an approximately regular rate, (2) EEG waves or complexes occurring intermittently at approximately regular intervals, generally of one to several seconds. (See periodic discharges).	**Periodico:** si applica a: (1) onde o complessi EEG che si ripetono in una sequenza ad intervalli per lo più regolari; (2) onde o complessi EEG intermittenti che si ripetono ad intervalli approssimativamente regolari, generalmente da uno a diversi secondi. (Vedi scariche periodiche).

**Periodic discharges (PDs)**: Repetition of a waveform with relatively uniform morphology and duration, with a quantifiable inter-discharge interval between consecutive waveforms, and recurrence of the waveform at nearly regular intervals. Comments: PDs may be generalized (GPDs), lateralized (LPDs), bilateral independent (BIPDs). Old nomenclature for these new terms are GPEDs (=GPDs), PLEDs (=LPDs) and BIPLEDs (=BIPDs). The use of “epileptiform” as an interpretative term is now avoided, since these periodic patterns may or may not be associated with clinical seizures (Hirsch et al., 2013).	**Scariche periodiche (PDs, periodic discharges):** ripetizione di grafoelementi a morfologia e durata relativamente uniforme, con un intervallo interscarica quantificabile e ripetizione a intervalli quasi regolari. Commenti: Le PDs possono essere generalizzate (GPDs, generalized periodic discharges), lateralizzate (LPDs, lateralized periodic discharges), bilaterali indipendenti (BIPDs, bilateral independent periodic discharges). La precedente nomenclatura comprendeva i termini di GPEDs (= GPDs), PLEDs (= LPDs) e BIPLEDs (= BIPDs) (N.d.T.: nell’acronimo “E” sta per epilettiforme). L'uso del termine “epilettiforme” è sconsigliato, poiché questi pattern periodici possono essere o no associati a crisi cliniche (Hirsch et al., 2013).

**Periodic lateralized epileptiform discharges (PLEDs)**: Use of term discouraged. See lateralized periodic discharges (preferred term).	**Scariche epilettiformi periodiche lateralizzate (PLEDs, periodic lateralized epileptiform discharges)**: l'uso del termine è sconsigliato. Vedi scariche periodiche lateralizzate (termine consigliato).

**Phase**: (1) Time or polarity relationships between a point on a wave displayed in a derivation and the identical point on the same wave recorded simultaneously in another derivation. (2) Time or angular relationships between a point on a wave and the onset of the cycle of the same wave. Usually expressed in degrees or radians.	**Fase:** (1) relazione temporale o di polarità tra un punto di un'onda registrata su una derivazione e un punto identico sulla medesima onda simultaneamente registrata su un'altra derivazione; (2) relazione temporale o angolare tra un punto su un'onda e l'inizio del ciclo della stessa onda; solitamente viene espresso in gradi o radianti.

**Phase reversal:** Simultaneous trace deflections in opposite directions from two or more channels in a bipolar recording montage. Assuming a single generator, phase reversal is due to the same signal being applied to the input terminal 2 of one differential amplifier and to the input terminal 1 of the other amplifier. Comment: When observed in two linked bipolar channels, phase reversal indicates that the potential field is maximal or minimal at or near the electrode common to such derivations. A phase reversal seen in a referential recording, when assessed using mapping of the potential fields, indicates that the dipole source is horizontally located in the sulcal wall across the borderline of the two fields of opposite polarity. (See bipolar and referential montages, dipole, input terminal).	**Inversione di fase:** simultanea deflessione in direzione opposta di una traccia registrata da due o più canali in montaggio bipolare. Se si assume un unico generatore, l'inversione di fase è dovuta allo stesso segnale elettrico che simultaneamente è in ingresso al terminale 2 di un amplificatore differenziale e al terminale 1 di un altro amplificatore. Commento: quando si registra su due canali bipolari collegati, l'inversione di fase indica che il potenziale elettrico è massimo o minimo in corrispondenza o in prossimità dell'elettrodo comune a tali derivazioni. Un'inversione di fase in una registrazione referenziale, quando si valuta il mappaggio dei potenziali di campo, indica che la sorgente del dipolo ha una disposizione orizzontale nella parete del solco all'interfaccia fra i due campi di polarità opposta. (Vedi montaggi bipolari e referenziali, dipolo, terminale di ingresso).

**Photic driving:** Physiologic response consisting of periodic activity elicited over the posterior regions of the head, usually induced by repetitive photic stimulation at frequencies of about 1–30 Hz. Comments: (1) term should be limited to activity time-locked to the stimulus and of frequency identical or harmonically related to the stimulus frequency. (2) Photic driving should be distinguished from the visual evoked potentials elicited by isolated flashes of light or flashes repeated at low frequencies (<5 Hz). (See photic stimulation).	**Trascinamento fotico:** risposta fisiologica che consiste in un'attività periodica che si registra sulle regioni posteriori dello scalpo, tipicamente indotta dalla stimolazione luminosa intermittente (SLI) alle frequenze di circa 1-30 Hz. Commenti: (1) il termine dovrebbe essere limitato all'attività tempo-correlata allo stimolo fotico e con una frequenza identica oppure un'armonica della frequenza dello stimolo stesso. (2) Il trascinamento fotico dovrebbe essere distinto dai potenziali evocati visivi elicitati da flash isolati oppure da flash ripetuti in treni a basse frequenze (<5 Hz). (Vedi stimolazione fotica).

**Photic evoked potential (PEP**): Evoked potential generated in the occipital cortex in response to flash stimulation. Synonym: Flash EP.	**Potenziale evocato visivo da flash (PEV-F):** potenziale evocato generato nella corteccia occipitale in risposta alla stimolazione con flash. Sinonimo: potenziale evocato da stimolo fotico.

**Photic stimulation**: Delivery of intermittent flashes of light to the eyes of a subject, usually from 1 to 60 Hz. Used as EEG activation procedure. Synonym: intermittent photic stimulation (IPS).	**Stimolazione luminosa intermittente (SLI):** metodica di attivazione dell'EEG caratterizzata dall'esposizione del paziente a treni di flash alle frequenze fra 1 e 60 Hz. Sinonimo: stimolazione fotica intermittente (IPS, intermittent photic stimulation).

**Photic stimulator:** Device for delivering intermittent flashes of light.	**Stimolatore luminoso:** dispositivo per la generazione di flash di luce intermittente.

**Photomyogenic response**: A non-cerebral response to intermittent photic stimulation characterized by the appearance in the record of brief repetitive muscle spikes (electromyography artifact) over the anterior regions of the head. These often increase gradually in amplitude as stimuli are continued and cease promptly when the stimulus is withdrawn. Comments: (1) this response is frequently associated with flutter of the eyelids and vertical oscillations of the eyeballs and sometimes with discrete jerking mostly involving the musculature of the face and head, (2) it is a physiological artifact contaminating the EEG.	**Risposta fotomiogenica:** un tipo di risposta alla stimolazione luminosa intermittente, di origine non cerebrale, caratterizzata dalla comparsa di attività EMGrafica artefattuale sulle derivazioni anteriori dello scalpo. Questi potenziali muscolari aumentano gradualmente in ampiezza quando gli stimoli luminosi si protraggono e cessano bruscamente al termine della stimolazione. Commenti: (1) questa risposta è frequentemente associata ad una contrazione ripetitiva delle palpebre, ad una deviazione verticale dei globi oculari e talora ad una contrazione evidente della muscolatura del volto e del capo; (2) è ritenuta un artefatto di significato fisiologico che contamina l'EEG.

**Photoparoxysmal response (PPR)**: Abnormal response to intermittent photic stimulation characterized by spike-and-slow-wave or polyspike-and-slow-wave complexes. Responses are subclassified in to 4 phenotypically different types, from focal occipital spikes (type 1 PPR) time-locked to the flashes to generalized (type 4 PPR) epileptiform discharges, which may outlast the stimulus by a few seconds. Comment: only the more generalized spike-and-wave responses (type 3 and 4 PPRs) show a strong association with epilepsy.	**Risposta fotoparossistica (PPR, photoparoxysmal response):** risposta anomala alla stimolazione luminosa intermittente caratterizzata da complessi di punta-onda lenta e polipunta-onda-lenta. La classificazione ne prevede 4 tipi: dalle punte focali occipitali che sono tempo correlate al flash (tipo 1) alle scariche epilettiformi generalizzate (tipo 4), che possono peraltro persistere dopo la fine della stimolazione per alcuni secondi. Commento: solo le risposte caratterizzate da complessi punta-onda-lenta più generalizzati (tipo 3 e 4) mostrano una significativa associazione con alcune forme di epilessia.

**Polarity convention:** International agreement whereby differential EEG amplifiers are constructed so that negativity at input terminal 1 relative to input terminal 2 of the same amplifier results in an upward trace deflection. For example, for a bipolar derivation C3-Cz (input 1 terminal -input terminal 2), an ‘upward deflection’ implies that C3 is more negative than Cz, while a ‘downward deflection’ implies that Cz is more negative than C3. Comment: this convention is contrary to that prevailing in some other biological and non-biological fields. (See input terminal 1 and 2).	**Convenzione di polarità:** accordo internazionale in base al quale gli amplificatori EEG differenziali sono costruiti in modo che la negatività all'ingresso 1 rispetto all’ingresso 2 dello stesso amplificatore provochi una deflessione della traccia verso l'alto. Per esempio, per una derivazione bipolare C3-Cz (ingresso 1 - ingresso 2), una “deflessione verso l'alto” implica che C3 è più negativo di Cz, mentre una “deflessione verso il basso” implica che Cz è più negativo di C3. Commento: questa convenzione è contraria a quella prevalente in alcuni altri campi biologici e non biologici. (Vedi terminale d'ingresso 1 e 2).

**Polarity, EEG wave:** Sign of potential difference, either positive or negative, existing at a given time between one electrode and another electrode (see polarity convention); which may be an exploring and reference electrode in a referential derivation, or two exploring electrodes in a bipolar derivation.v	**Polarità, onda EEG:** segno della differenza di potenziale, positivo o negativo, esistente in un dato momento tra un elettrodo ed un altro elettrodo (vedi convenzione di polarità); può essere un elettrodo attivo ed uno di riferimento nel caso di un montaggio referenziale, o due elettrodi attivi nel caso di un montaggio bipolare.

**Polygraphic recording:** Simultaneous monitoring of multiple physiological parameters such as the EEG, respiration, electrocardiogram, electromyogram, eye movements (electrooculogram), oxygen saturation, and leg movements, etc. Comment: some may be part of routine EEG recording but are recommended in polysomnography. (See polysomnography).	**Registrazione poligrafica:** monitoraggio simultaneo di più parametri fisiologici come l'EEG, la respirazione, l'elettrocardiogramma, l'elettromiografia, l'elettro-oculogramma, la saturazione di ossigeno, i movimenti delle gambe, ecc. Commento: alcuni possono far parte della registrazione EEG di routine ma sono raccomandati nella polisonnografia. (Vedi polisonnografia).

**Polymorphic activity:** Irregular EEG waves having multiple forms, which may also vary in frequency and amplitude. Synonym: irregular.	**Attività polimorfa:** onde EEG irregolari con morfologia variabile, anche con variazione in frequenza e ampiezza. Sinonimo: irregolare.

**Polyphasic wave:** Wave consisting of more than two phases developed on alternating sides of the baseline (for example see triphasic wave).	**Onda polifasica:** onda composta da più di due fasi con attraversamento della linea di base (per esempio vedi onda trifasica).

**Polysomnography (PSG**): Polygraphic recording of sleep including EEG, electrooculogram, electromyogram (chin and leg), airflow parameters and oxygen saturation, along with video. A test used to diagnose sleep disorders. Synonym: sleep studies.	**Polisonnografia (PSG):** registrazione poligrafica del sonno che include EEG, elettro-oculogramma, elettromiografia (mento e gamba), flusso oro-nasale e saturazione dell'ossigeno, associati alla video registrazione. Test utilizzato per diagnosticare i disturbi del sonno. Sinonimo: studi del sonno.

**Polyspike-and-slow-wave complex:** An epileptiform pattern consisting of two or more spikes associated with one or more slow waves (see epileptiform pattern). Synonym: multiple spike-and-slow-wave complex (use of term discouraged).	**Complesso di polipunte ed onde lente:** un grafoelemento epilettiforme che consiste in due o più punte associate ad una o più onde lente (vedi grafoelemento epilettiforme). Sinonimo: complessi di punta e onda lenta multipli (l'uso del termine è sconsigliato).

**Polyspike complex:** A sequence of two or more spikes. Comment: may or may not be an epileptiform pattern (for examples see generalized paroxysmal fast activity and wicket spikes, respectively). Synonym: multiple spike complex (use of term discouraged).	**Complesso di polipunte:** una sequenza di due o più punte. Commento: può essere o non essere un pattern epilettiforme (per esempio vedi attività rapida e parossistica generalizzata e 'wicket spikes', rispettivamente). Sinonimo: complesso di punte multiple (l'uso del termine è sconsigliato).

**Positive occipital sharp transient of sleep (POSTS)**: A normal graphoelement. Sharp transient maximal over the occipital regions, positive relative to other areas, apparently occurring spontaneously during sleep. May be single or repetitive. Amplitude varies but is generally below 50 µV.	**Potenziale occipitale puntuto positivo del sonno (POSTS, positive occipital sharp transient of sleep):** un grafoelemento fisiologico. Di ampiezza massima sulle regioni occipitali, positivo rispetto ad altre aree, che si osserva spontaneamente durante il sonno. Può essere singolo o presentarsi in sequenze. L'ampiezza varia ma è generalmente inferiore a 50 µV.

**Positive rolandic sharp waves (PRSW)**: Abnormal transients in neonatal period, surface positive, broad-based sharp waves with duration of <0.5 s, localized to central regions (C3/C4/Cz). Associated with white matter injury in preterm infants. Synonym: positive sharp wave transients.	**Onde puntute rolandiche positive (PRSW, positive rolandic sharp waves):** potenziali anomali nel periodo neonatale, positivi, di morfologia aguzza ma con base di durata di <0,5 s, localizzati alle regioni centrali (C3/C4/Cz). Associati a lesioni della sostanza bianca nei neonati pretermine. Sinonimo: transienti di onde aguzze positive.

**Posterior basic rhythm (PBR)**: Synonym: posterior dominant rhythm.	**Ritmo di base posteriore (PBR, posterior basic rhythm):** sinonimo: ritmo dominante posteriore.

**Posterior dominant rhythm (PDR)**: Rhythmic activity seen at the occipital or parietal regions predominantly during wakefulness while the eyes are kept closed. Comment: usually in the alpha frequency band in healthy adults. Synonym: posterior basic rhythm.	**Ritmo dominante posteriore (PDR, posterior dominant rhythm):** attività ritmica presente nelle regioni occipitali o parietali prevalentemente durante la veglia mentre gli occhi sono tenuti chiusi. Commento: di solito nella banda di frequenza alfa negli adulti sani. Sinonimo: ritmo di fondo.

**Posterior slow waves of youth:** A normal graphoelement. Isolated slow waves intermixed with the posterior dominant rhythm in young people (typically 4–25 years of age). See slow-fused transients.	**Onde lente posteriori del giovane:** grafoelemento fisiologico. Onde lente isolate mescolate al ritmo dominante posteriore nei giovani (tipicamente 4-25 anni). Vedi onde lente intermittenti.

**Potential:** (1) Strictly: voltage. (2) Loosely: synonym of electrical activity (waveforms) generated by the nervous system.	**Potenziale:** (1) in senso stretto: voltaggio; (2) secondo la terminologia EEG: sinonimo di attività elettrica (forme d'onda) generata dal sistema nervoso.

**Potential field:** Amplitude distribution of the negative and positive potentials of an EEG signal at the surface of the head, or cerebral cortex or in the depth of the brain, measured at a given instant in time. Represented in diagrams by color codes for negativity and positivity, and by equipotential lines (see map isopotential, power spectrum).	**Campo di potenziale:** distribuzione di ampiezza dei potenziali negativi e positivi di un segnale EEG sullo scalpo, sulla corteccia cerebrale o nella profondità del cervello, misurata in un dato istante di tempo. Rappresentata nei diagrammi da codici colore per la negatività e la positività, e dalle linee equipotenziali (vedi mappa isopotenziale, spettro di potenza).

**Power Spectrum:** Display of the distribution of frequency-specific power (i.e. amplitude squared), with the waveform frequency plotted on the abscissa and the power plotted on the ordinate of a spectrogram display. (See frequency spectrum, quantitative EEG).	**Spettro di potenza:** visualizzazione della distribuzione della potenza specifica della frequenza (cioè l'ampiezza al quadrato), con la frequenza della forma d'onda rappresentata sull'ascissa e la potenza rappresentata sull'ordinata di uno spettrogramma. (Vedi spettro di frequenza, EEG quantitativo).

**Premature temporal theta:** Normal graphoelement of preterm infants (24–34 weeks post menstrual age, highest incidence 29–31 weeks). Temporal sharp transients occurring in bursts of rhythmic sharp waves at 4–7 c/s (25–125 µV). Typically bilateral but often asynchronous. Synonyms: rhythmic temporal theta, temporal sawtooth waves.	**Theta temporale del prematuro:** grafoelemento normale dei neonati pretermine (24-34 settimane di età gestazionale, massima incidenza 29-31 settimane). Sequenze di onde ritmiche puntute a 4-7 Hz (25-125 µV). Tipicamente bilaterali ma spesso asincrone. Sinonimi: theta temporale ritmico, onde a dente di sega temporali.

**Prevalence:** Proportion of the record or a specified epoch that includes a particular EEG pattern. For example: ≥90% is continuous, 50–89% is abundant, 10–49% is frequent, 1–9% is occasional, and <1% is rare. Comment: the equivalent descriptor for transients or isolated discharges is incidence or quantity. (See incidence, quantity).	**Prevalenza:** percentuale della registrazione o di una determinata epoca che include un particolare grafoelemento EEG. Per esempio: ≥90% è continuo, 50-89% è abbondante, 10-49% è frequente, 1-9% è occasionale, e <1% è raro. Commento: il descrittore equivalente per i transienti o le scariche isolate è l'incidenza o la quantità. (Vedi incidenza, quantità).

**Propagation:** The active neural process whereby electric activity spreads from one area of the brain to another. For example, propagation from a focus to the contralateral, homologous brain region, which then leads to bilateral synchronous discharges, is called secondary bilateral synchrony or secondary generalization. (See secondary bilateral synchrony, volume conduction).	**Propagazione:** il processo neurale attivo per cui l'attività elettrica si diffonde da un'area del cervello ad un'altra. Per esempio, la propagazione da un focus alla regione cerebrale omologa controlaterale, che poi porta a scariche sincrone bilaterali, si chiama sincronia bilaterale secondaria o generalizzazione secondaria. (Vedi sincronia bilaterale secondaria, volume condotto).

**Psychomotor variant:** Use of term discouraged. Synonym: rhythmic temporal theta burst of drowsiness.	**Variante psicomotoria:** uso del termine sconsigliato. Sinonimo: burst ritmico temporale theta della sonnolenza.

**Quantitative EEG (qEEG)**: Processing and analysis of portions of digitized EEG data, such as frequency specific power typically derived by Fourier transform, displayed in various formats. Statistical variables can be compared, such as wave phase and coherence. Clinically it is most used in the intensive care unit to evaluate cerebral function trends and following treatment interventions. (See map voltage, power spectrum, continuous EEG).	**EEG quantitativo (qEEG, quantitative EEG):** elaborazione ed analisi di porzioni di dati EEG digitalizzati, come la potenza specifica di frequenza tipicamente derivata dalla trasformata di Fourier, visualizzata in vari formati. Le variabili statistiche possono essere confrontate, come la fase dell'onda e la coerenza. Clinicamente è più utilizzato nell'unità di terapia intensiva per valutare l'andamento della funzione cerebrale spontanea od in seguito ad interventi di trattamento. (Vedi mappa di voltaggio, spettro di potenza, EEG continuo).

**Quantity:** Amount of EEG activity with respect to number of transients or waves. For example, interictal epileptiform discharges: ≥1/10 s are abundant, ≥1/min but less than 1/10 s are frequent, ≥1/h but less than 1/min are occasional, and <1/h are rare. Synonym: incidence. (See also prevalence).	**Quantità:** quantità di attività EEG di fondo rispetto al numero di transienti o onde. Per esempio, scariche epilettiformi interictali: ≥1/10 s sono abbondanti, ≥1/min ma meno di 1/10 s sono frequenti, ≥1/h ma meno di 1/min sono occasionali, e <1/h sono rare. Sinonimo: incidenza. (Vedi anche prevalenza).

**Quasiperiodic: **Loosely: applies to EEG waves or complexes that occur at random intervals, which only approach regularity and that are not an exact repeating frequency. Strictly: determined by quantitative computer analysis and defined as having a cycle length (i.e. period) varying by 25–50% from one cycle to the next in the majority (>50%) of cycle pairs. (Synonym: pseudoperiodic). (Hirsch et al., 2013).	**Quasiperiodico:** genericamente: si applica ad onde o complessi EEG che si presentano ad intervalli casuali, che si avvicinano solo alla regolarità e che non presentano una frequenza di ripetizione esatta. In senso stretto: determinato dall'analisi quantitativa al computer e definito come avente una lunghezza del ciclo (cioè il periodo) che varia del 25-50% da un ciclo all'altro nella maggioranza (>50%) delle coppie di cicli. (Sinonimo: pseudoperiodico). (Hirsch et al., 2013).

**Quiet sleep:** Normal sleep stage in neonates characterized by eye closure, absence of rapid eye movements, and scant body movements, except for occasional sucking activity or myoclonic jerks. The EEG shows tracé alternant in term and near term infants and tracé discontinue (discontinuous pattern) in preterm infants; the interburst interval depend on the post menstrual age. (See active sleep, tracé alternant and tracé discontinue).	**Sonno quieto:** fase fisiologica del sonno nei neonati, caratterizzata dal punto di vista comportamentale dalla presenza di occhi chiusi, assenza di movimenti oculari rapidi e da scarsi movimenti del corpo, ad eccezione di occasionale attività di suzione o di fulminee e parcellari contrazioni muscolari. L'EEG si caratterizza nei neonati a termine e quasi a termine per un pattern di “tracciato alternante” (sonno quieto alternante) (N.d.T.: oppure per un'attività di ampio voltaggio lenta). Nei neonati pretermine è presente invece un “tracciato discontinuo”; la durata delle attenuazioni (N.d.T.: se si tratta di tracciato alternante) o soppressioni (N.d.T.: se si tratta di tracciato discontinuo) dipende dall'età gestazionale. (Vedi sonno attivo, tracciato alternante, tracciato discontinuo).

**Reactivity:** A phenomena in which the EEG pattern clearly and reproducibly changes with sensory (visual, auditory or noxious) stimulation. Changes may occur in frequency, morphology and/or amplitude, including attenuation of activity after the stimulus. Comment: appearance of muscle activity or eye blink artifacts or heart rate does not qualify as reactive. In general terms reactivity of the EEG in comatose patients is a favorable prognostic sign.	**Reattività:** un fenomeno in cui l'attività EEG si modifica in modo chiaro e riproducibile con la stimolazione sensoriale (visiva, uditiva o nociva). I cambiamenti possono verificarsi in frequenza, morfologia e/o ampiezza, compresa l'attenuazione dell'attività dopo lo stimolo. Commento: la comparsa di attività muscolare o di artefatti di battito degli occhi o di frequenza cardiaca non si qualifica come reattività. In termini generali la reattività dell'EEG nei pazienti in coma è un segno prognostico favorevole.

**Record:** The end product of the EEG recording process. Synonyms: recording, tracing.	**Tracciato:** il prodotto finale del processo di registrazione EEG. Sinonimo: registrazione.

**Recording:** (1) The process of obtaining an EEG record. Synonym: tracing. (2) The end product of the EEG recording process, most commonly on to digital storage media. Synonyms: record, tracing.	**Registrazione:** (1) il processo di acquisizione di una registrazione EEG. Sinonimo: tracciato; (2) il prodotto finale del processo di registrazione EEG, più comunemente su un supporto di memorizzazione digitale. Sinonimo: tracciato.

**Record of electrocerebral inactivity**: See electrocerebral inactivity.	**Registrazione di inattività elettrocerebrale:** vedi inattività elettrocerebrale.

**Reference electrode:** (1) In general: any electrode against which the potential variations of another electrode are measured. (2) Specifically: a suitable reference electrode is historically connected to the input terminal 2 of an EEG amplifier and placed so as to minimize the likelihood of recording the same EEG activity as detected by an exploring electrode (connected to the input terminal 1 of the same amplifier), or of other activities. Comments: (1) Whatever the location of the reference electrode, the possibility that it might be affected by appreciable EEG potentials should always be considered. (2) A reference electrode connected to the input terminal 2 of all EEG amplifiers is referred to as a common reference electrode. (See exploring electrode).	**Elettrodo di referenza: **(1) in generale: qualsiasi elettrodo rispetto al quale si misurano le variazioni di potenziale di un altro elettrodo; (2) in particolare: un elettrodo di riferimento (o referenza) adeguato è storicamente collegato all'ingresso 2 di un amplificatore EEG e posizionato in modo da ridurre al minimo la probabilità di registrare la stessa attività EEG rilevata dall'elettrodo esplorante (collegato all'ingresso 1 dello stesso amplificatore), o di altre attività. Commenti: (1) qualunque sia la posizione dell'elettrodo di referenza, la possibilità che possa essere interessato da potenziali EEG apprezzabili dovrebbe sempre essere considerato; (2) un elettrodo di referenza collegato all'ingresso 2 di tutti gli amplificatori EEG viene definito elettrodo di referenza comune. (Vedi elettrodo di esplorazione, elettrodo di referenza comune).

**Referential derivation:** Recording from a pair of electrodes consisting of an exploring electrode historically connected to the input terminal 1 and a reference electrode usually connected to the input terminal 2 of an EEG amplifier (see exploring and reference electrode, input terminal 1 and 2, referential montage).	**Derivazione referenziale**: registrazione da una coppia di elettrodi costituita da un elettrodo esplorante collegato all'ingresso 1 e da un elettrodo di riferimento solitamente collegato all'ingresso 2 di un amplificatore EEG (vedi elettrodo esplorante e di riferimento, terminale di ingresso 1 e 2, montaggio referenziale, elettrodo di referenza comune).

**Referential montage:** A montage consisting of referential derivations. Comment: a referential montage in which the reference electrode is common to multiple derivations is referred to as a common reference montage (see referential derivation).	**Montaggio referenziale:** un montaggio composto da derivazioni referenziali. Commento: un montaggio referenziale in cui l'elettrodo di referenza è comune a più derivazioni è indicato come un montaggio di referenza comune (vedi derivazione referenziale, elettrodo di referenza comune).

**Reformatting:** Transformation of digitized EEG into different montages. Reformatting requires that the raw EEG signal is recorded to a common reference electrode. Only those electrodes can be included in the reformatting montages which are connected to amplifier input 1.	**Riformattazione:** trasformazione dell'EEG digitalizzato in diversi montaggi. La riformattazione richiede che il segnale EEG grezzo sia registrato su un elettrodo di referenza comune. Nei montaggi referenziali possono essere inclusi solo quegli elettrodi che sono collegati all'ingresso 1 dell'amplificatore.

**Regional:** EEG activity that is limited to a region of the scalp overlying a lobe (i.e. frontal, temporal, parietal, occipital). (See focal, multiregional).	**Regionale:** attività EEG limitata a una regione dello scalpo sovrastante un lobo (per esempio frontale, temporale, parietale, occipitale). (Vedi focale, multiregionale).

**Regular:** Applies to waves or complexes of approximately constant period and relatively uniform appearance. Synonyms: rhythmic, monomorphic (use of latter term discouraged).	**Regolare:** si applica a onde o complessi di periodo approssimativamente costante e di aspetto relativamente uniforme. Sinonimi: ritmico, monomorfo (l'uso di quest'ultimo termine è sconsigliato).

**REM:** Rapid eye movements characterizing REM sleep. Conjugate, irregular, sharply peaked eye movements with an initial deflection lasting <0.5 s (see REM sleep). Comment: not to be confused with saccadic eye movements in an awake subject during visual scanning.	**REM (rapid eye movements):** movimenti oculari rapidi che caratterizzano il sonno REM. Movimenti oculari coniugati, irregolari, a picco, con una deflessione iniziale della durata di <0,5 s (vedi sonno REM). Commento: da non confondere con i movimenti oculari saccadici in un soggetto sveglio durante la scansione visiva.

**REM atonia:** Normal reduction of tonic skeletal muscle activity during REM sleep.	**Atonia REM:** normale riduzione dell'attività muscolare scheletrica tonica soprattutto assiale durante il sonno REM.

**REM sleep:** Sleep stage characterized by low amplitude mixed frequency EEG activity, episodic bursts of predominantly horizontal rapid eye movements (REM) and reduction of axial tonic muscle activity; frequently associated with dreams; phasic muscle activity, sawtooth waves and changes in respiration may occur. (See active sleep, Non-REM sleep).	**Sonno REM:** fase del sonno caratterizzata da attività EEG a bassa ampiezza e frequenza mista, burst episodici di movimenti oculari rapidi prevalentemente orizzontali (REM) e riduzione dell'attività muscolare tonica assiale; spesso associata ai sogni; possono verificarsi attività muscolare fasica, onde a dente di sega e cambiamenti nella respirazione. (Vedi sonno attivo, sonno non REM).

**Resistance-capacitance (RC) coupled amplifier:** A multistage amplifier wherein successive stages of amplifiers are connected (coupled) using a combination of a resistor and a capacitor. (See also Direct coupled amplifier).	**Amplificatore accoppiato resistenza-capacità (RC):** un amplificatore multistadio in cui gli amplificatori sono collegati (accoppiati) utilizzando una combinazione di una resistenza e un condensatore. (Vedi anche Amplificatore ad accoppiamento diretto).

**Resolution:** The resolution of an analogue–digital (AD) converter (see digital EEG) is specified in binary digits or “bits”, which approximates to the fineness of detail in the amplitude domain. For example, a dynamic range of ±1023 µV (a total span of 2046 µV), converted at 12-bit resolution, will allow the digitized signal to take on values every 0.5 µV.	**Risoluzione:** la risoluzione di un convertitore analogico-digitale (AD) (vedi EEG digitale) è specificata in cifre binarie o “bit”, che determina l'entità del dettaglio della risoluzione nel dominio dell'ampiezza. Per esempio, una gamma dinamica di ±1023 µV (uno span totale di 2046 µV), convertita con una risoluzione di 12 bit, permetterà al segnale digitalizzato di assumere valori ogni 0,5 µV.

**Rhythm:** EEG activity consisting of waves of approximately constant period.	**Ritmo:** attività EEG costituita da onde con un periodo approssimativamente costante.

**Rhythmic:** Applied to regular waves occurring at a constant period and of relatively uniform morphology. Synonyms: regular, monomorphic (use of latter term discouraged).	**Ritmico:** termine applicato a onde regolari che si ripetono con un periodo costante e di morfologia relativamente uniforme. Sinonimi: regolare, monomorfo (l'uso di quest'ultimo termine è sconsigliato).

**Rhythmic temporal theta:** A normal graphoelement in preterm infants (GA 24–34 weeks of post menstrual age, maximal 29–32 weeks). Consisting of brief theta bursts (4.5–6 c/s) over the temporal regions, typically symmetrical but not necessarily synchronous. Synonyms: premature temporal theta, temporal sawtooth bursts.	**Theta temporale ritmico:** un grafoelemento normale nei neonati pretermine (24-34 settimane di età gestazionale, massimo 29-32 settimane). Composto da brevi burst di attività theta (4,5-6 Hz) sulle regioni temporali, tipicamente simmetriche ma non necessariamente sincrone. Sinonimi: theta temporale prematuro, burst temporali a dente di sega.

**Rhythmic temporal theta burst of drowsiness:** A normal variant. Characteristic burst of 4–7 c/s waves whose morphology is frequently notched by faster waves, occurring over the temporal regions of the head during drowsiness, bilaterally or independently. Synonyms: rhythmic midtemporal discharge, psychomotor variant pattern (use of terms discouraged).	**Burst theta temporale ritmico della sonnolenza:** variante fisiologica. Caratteristico burst di onde a 4-7 Hz la cui morfologia è frequentemente dentellata da onde più rapide, che si verificano sulle regioni temporali dello scalpo durante la sonnolenza, bilateralmente o indipendentemente. Sinonimi: scarica ritmica medio-temporale, pattern di variante psicomotoria (l'uso dei termini è sconsigliato).

**Rhythm of alpha frequency:** (1) In general: any rhythm in the alpha band. (2) Specifically: term should be used to designate those activities in the alpha band which differ from the alpha rhythm as regards their topography and/or reactivity and do not have specific appellations (such as mu rhythm and alpha coma) (see alpha rhythm).	**Ritmo di frequenza alfa:** (1) in generale: qualsiasi ritmo nella banda alfa; (2) in particolare: il termine dovrebbe essere usato per designare quelle attività nella banda alfa che differiscono dal ritmo alfa per quanto riguarda la loro topografia e/o reattività e non hanno appellativi specifici (come ritmo mu e coma alfa) (vedi ritmo alfa).

**Ripples:** Part of the high frequency oscillations (HFOs) bandwidth, usually defined as being in the range of 80–250 Hz (see high frequency oscillations).	**Ripples:** parte della larghezza di banda delle oscillazioni ad alta frequenza, di solito nell'intervallo di frequenza di 80-250 Hz (vedi oscillazioni ad alta frequenza).

**Rolandic spikes:** Uni-or bilateral triphasic sharp waves in the centro-temporal area seen in childhood epilepsy with centro-temporal spikes. They often have a tangential (horizontal) dipole oriented with negativity in centro-temporal/parietal areas and positivity in the frontal region, and increase during sleep with a tendency to appear in series. Synonym: centro-temporal spikes or discharges (see benign epileptiform discharges of childhood: use of term discouraged).	**Punte rolandiche:** onde puntute trifasiche unilaterali o bilaterali nell'area centro-temporale, di riscontro nell'epilessia infantile con punte centro-temporali. Spesso hanno un dipolo tangenziale (orizzontale) orientato con negatività nelle aree centro-temporali/parietali e positività nella regione frontale, aumentano durante il sonno con una tendenza a comparire in scariche. Sinonimo: punte o scariche centro-temporali (vedi scariche epilettiformi benigne dell'infanzia: l'uso del termine è sconsigliato).

**Sampling rate:** Frequency in Hz used for sampling the digital EEG. Sampling rates in the 250–500 Hz range are common. Higher sampling rates may be appropriate for specific applications, for example 1000–2000 Hz in intra-cranial depth EEG. (See analog-to-digital conversion, Nyquist theorem).	**Frequenza di campionamento:** frequenza in Hz utilizzata per indicare il campionamento dell'EEG digitale. Le frequenze di campionamento nell'intervallo 250-500 Hz sono comuni. Frequenze di campionamento più elevate possono essere appropriate per applicazioni specifiche, per esempio 1000-2000 Hz nell'EEG intracranico. (Vedi conversione analogico-digitale, teorema di Nyquist).

**Sawtooth (saw-tooth) waves:** Brief runs of rhythmic sharp waves of 4–7 c/s, often of quite high amplitude (up to 125 µV). (See premature temporal theta).	**Onde a dente di sega:** brevi sequenze di onde ritmiche aguzze a 4-7 Hz, spesso di ampiezza piuttosto elevata (fino a 125 µV). (Vedi theta temporale prematuro).

**Scalp electrode:** Electrode held against, attached to, or needle inserted in the scalp.	**Elettrodo dello scalpo:** elettrodo applicato (a tampone, etc.) od inserito (ago) nello scalpo.

**Scalp electroencephalogram:** Record of electrical activity of the brain by means of electrodes placed on the surface of the head. The term should be used only to distinguish between scalp and other electroencephalograms, such as intra-cranial depth electroencephalograms. In all other instances, a scalp electroencephalogram should be referred to simply as an electroencephalogram (EEG).	**Elettroencefalogramma di scalpo:** registrazione dell'attività elettrica del cervello per mezzo di elettrodi posti sulla superficie dello scalpo. Il termine dovrebbe essere usato solo per distinguere tra lo scalpo e altri elettroencefalogrammi, come gli elettroencefalogrammi di profondità intracranici. In tutti gli altri casi, un elettroencefalogramma derivato dallo scalpo dovrebbe essere indicato semplicemente come un elettroencefalogramma (EEG).

**Scalp electroencephalography:** Technique of recording scalp electroencephalograms. Should be referred to simply as electroencephalography (EEG).	**Elettroencefalografia da scalpo:** tecnica di registrazione degli elettroencefalogrammi da scalpo. Dovrebbe essere indicato semplicemente come elettroencefalogramma (EEG).

**Secondary bilateral synchrony:** Spreading by propagation of an initially focal or regional epileptiform discharge to become generalized (see generalized). Synonym: secondary generalization.	**Sincronia bilaterale secondaria:** diffusione per propagazione di una scarica epilettiforme inizialmente focale o regionale che diventa generalizzata (vedi generalizzata). Sinonimo: generalizzazione secondaria.

**Seizure pattern, EEG:** Phenomenon consisting of repetitive epileptiform EEG discharges at >2 c/s and/or characteristic pattern with quasi-rhythmic spatio-temporal evolution (i.e. gradual change in frequency, amplitude, morphology and location), lasting at least several seconds (usually >10 s). Two other short duration (<10 s) EEG seizure patterns are: electrodecrement and low voltage fast activity seen during clinically apparent epileptic seizures. Frequent interictal epileptiform discharges are usually not associated with clinical seizures and thus should be differentiated from EEG seizure patterns. Comment: EEG seizure patterns unaccompanied by clinical epileptic manifestations should be referred to as electrographic or subclinical seizures. (See electrodecrement and low voltage fast activity). Synonym: ictal EEG pattern.	**Pattern di crisi, EEG:** fenomeno costituito da scariche epilettiformi EEG ripetitive a >2 Hz e/o con evoluzione spazio-temporale quasi ritmica (cioè che presentano un cambiamento graduale di frequenza, ampiezza, morfologia e localizzazione), della durata di almeno diversi secondi (di solito >10 s). Altre due tipologie di pattern EEG critici di breve durata (<10 s) sono: il pattern elettrodecrementale e l'attività rapida a basso voltaggio osservata durante crisi epilettiche clinicamente apparenti. Scariche epilettiformi interictali ad alta frequenza di solito non sono associate a crisi cliniche e quindi dovrebbero essere differenziate dalle crisi EEG. Commento: le crisi EEG non accompagnate da manifestazioni cliniche dovrebbero essere indicate come scariche elettrografiche o subcliniche. (Vedi elettrodecremento e attività rapido di basso voltaggio). Sinonimo: pattern EEG ictale.

**Sensitivity:** Ratio of input voltage to output trace deflection in an EEG channel. Sensitivity is measured in microvolts per millimeter (µV/mm). Example: Sensitivity=input voltage/output trace deflection=50μV/10mm=5μV/mm	**Sensibilità:** rapporto tra la tensione di corrente (voltaggio) di ingresso e la deflessione della traccia in un canale EEG. La sensibilità si misura in microvolt per millimetro (µV/mm). Esempio: Sensibilità=tensione d'ingresso/deflessione della traccia d'uscita=50μV/10mm=5μV/mm

**Sharp wave:** An epileptiform transient clearly distinguished from the background activity, although amplitude varies. A pointed peak at a conventional time scale and duration of 70–200 ms, usually with a steeper ascending phase when compared to the descending phase. Main component is generally negative relative to other areas, and may be followed by slow wave of the same polarity. Comments: (1) term should be restricted to epileptiform discharges, and does not apply to: (a) distinctive physiological events such as vertex sharp transients, lambda waves and positive occipital sharp transients of sleep, (b) sharp transients poorly distinguished from background activity (without or with a slow wave for example six Hz spike-and-slow-wave). (2) Sharp waves should be differentiated from spikes, i.e. transients having similar characteristics but shorter duration. However, it should be kept in mind that this distinction is largely arbitrary and primarily serves descriptive purposes.	**Onda puntuta:** un grafoelemento epilettiforme chiaramente distinto dall'attività di fondo, anche di ampiezza variabile. Deve avere un picco appuntito ed una durata di 70-200 ms, di solito con una branca ascendente più ripida rispetto alla branca discendente. La componente principale è generalmente negativa rispetto ad altre aree, e può essere seguita da un'onda lenta della stessa polarità. Commenti: (1) il termine dovrebbe essere limitato alle scariche epilettiformi, e non si applica a: (a) eventi fisiologici distintivi come le punte vertice, le onde lambda ed i transienti aguzzi occipitali positivi del sonno, (b) transienti aguzzi scarsamente distinti dall'attività di fondo (con o senza un'onda lenta, per esempio i complessi punta-onda-lenta a 6 Hz). (2) Le onde puntute devono essere differenziate dalle punte, cioè i transienti che hanno caratteristiche simili ma una durata inferiore. Tuttavia, va tenuto presente che questa distinzione è in gran parte arbitraria e serve principalmente a scopi descrittivi.

**Sharp-and-slow-wave complex:** An epileptiform pattern consisting of a sharp wave and an associated following slow wave, clearly distinguished from background activity. May be single or multiple. (See sharp wave).	**Complesso di onde puntute e onde lente**: un grafoelemento epilettiforme che consiste in un'onda puntuta seguita da un'onda lenta associata, chiaramente distinta dall'attività di fondo. Può essere singolo o multiplo. (Vedi onda puntuta).

**Simultaneous:** Occurring at the same time. Synonym: synchronous.	**Simultaneo:** che si verifica allo stesso tempo. Sinonimo: sincrono.

**Sine wave:** Wave having the form of a sine curve, describing a smooth periodic repetitive oscillation.	**Onda sinusoidale:** onda avente la forma di una curva sinusoidale, che descrive un'oscillazione periodica regolare e ripetitiva.

**Sinusoidal:** Term applies to EEG waves resembling sine waves (see sine wave).	**Sinusoidale:** termine che si applica alle onde EEG che assomigliano alle onde sinusoidali (vedi onda sinusoidale).

**Six Hz spike-and-slow-wave**: Spike-and-slow-wave complexes at 4–7 c/s, but mostly at 6 c/s occurring generally in brief bursts bilaterally and synchronously, symmetrically or asymmetrically, and either confined to or of larger amplitude over the posterior or anterior regions of the head. Amplitude varies but is generally smaller than that of spike-and-slow-wave complexes repeating at slower rates. Comment: when low amplitude, posterior and during drowsiness this pseudo-epileptiform pattern should be distinguished from epileptiform discharges. Synonym: phantom spike-and-wave (use of term discouraged).	**Complessi punta-onda-lenta a 6 Hz:** complessi punta-onda-lenta a 4-7 Hz, ma soprattutto a 6 Hz che si presentano generalmente in brevi scariche bilaterali e sincrone, simmetriche o asimmetriche, localizzate o di maggiore ampiezza sulle regioni posteriori o anteriori della testa. L'ampiezza varia ma è generalmente minore di quella dei complessi punta-onda-lenta che si ripetono a ritmi più lenti. Commento: quando è di bassa ampiezza, posteriore e durante la sonnolenza questo pattern pseudo-epilettiforme deve essere distinto dalle scariche epilettiformi. Sinonimo: punta-onda fantasma (l'uso del termine è sconsigliato).

**Sleep onset REM (SOREM)**: Occurrence of REMs less than 15 min after falling asleep, typically seen in association with narcolepsy (but may be seen in apneic patients and even healthy volunteers). (See REM).	**Insorgenza del sonno in fase REM (SOREM, sleep onset REM):** insorgenza di sonno REM entro 15 minuti dall'addormentamento, tipicamente si osserva in pazienti con narcolessia (ma può essere visto anche in pazienti con apnee e anche in soggetti sani). (Vedi REM).

**Sleep spindle:** A normal graphoelement. Train of distinct waves with frequency at 11–16 Hz (most commonly at 12–14 Hz) with a duration ≥0.5 s. Generally higher amplitude over the central regions of the head, occurring during sleep. Amplitude varies but is maximal using central derivations.	**Fuso del sonno:** un grafoelemento fisiologico. Treno di onde con frequenza a 11-16 Hz (più comunemente a 12-14 Hz) con una durata ≥0,5 s. Generalmente di maggiore ampiezza sulle regioni centrali, che si osserva/registra durante il sonno. L'ampiezza varia ma è massima nelle derivazioni centrali.

**Sleep stages:** Distinctive phases of sleep best demonstrated by polygraphic recordings of the EEG and other variables, including at least eye movements and activity of certain voluntary muscles. Comment: classified by various systems (see Iber et al., 2007, Silber et al., 2007, based on the frameworks of Dement and Kleitman, 1957, Rechtschaffen and Kales, 1968).	**Stadi del sonno:** fasi distinte del sonno meglio dimostrate da registrazioni poligrafiche dell'EEG e di altre variabili, compresi almeno i movimenti oculari e l'attività di alcuni muscoli volontari. Commento: classificato secondo vari criteri (vedi Iber et al., 2007, Silber et al., 2007, Dement and Kleitman, 1957, Rechtschaffen and Kales, 1968).

**Slow activity:** Any activity of frequency less than alpha rhythm, i.e. theta and delta bands.	**Attività lenta:** qualsiasi attività di frequenza inferiore al ritmo alfa, cioè le bande theta e delta.

**Slow alpha variant rhythms:** A normal variant. Rhythms mostly at 4–5 Hz, recorded over the posterior regions of the head, that behave like the posterior dominant rhythm in response to activation (i.e. are blocked or attenuated by attention, especially eye opening and mental effort). Generally alternate, or are intermixed, with the alpha rhythm to which they often are harmonically related. They may have a notched morphology but are not generally considered abnormal. Amplitude varies but is frequently close to 50 µV. Comment: slow alpha variant rhythms should be distinguished from posterior slow waves of youth, characteristic of children and adolescents and occasionally seen in young adults (see posterior dominant rhythm, posterior slow waves of youth).	**Ritmi lenti varianti dell'alfa:** rappresentano una variante fisiologica. Ritmi per lo più a 4-5 Hz, registrati nelle regioni posteriori, che si comportano come il ritmo dominante posteriore in risposta alle attivazioni (cioè sono bloccati o attenuati dall'attenzione, soprattutto dall'apertura degli occhi e dalla attività mentale). Generalmente si alternano, o sono frammiste, con il ritmo alfa al quale spesso sono armonicamente legati. Possono avere una morfologia dentellata ma non sono generalmente considerati anormali. L'ampiezza varia ma è spesso vicina ai 50 µV. Commento: i ritmi lenti alfa varianti devono essere distinti dalle onde lente posteriori del giovane, caratteristiche dei bambini e degli adolescenti e occasionalmente viste nei giovani adulti (vedi ritmo dominante posteriore, onde lente posteriori del giovane).

**Slow-fused transient:** A normal graphoelement. A sharply contoured component of the normal posterior dominant rhythm precedes a posterior slow wave of youth, giving the false impression of a spike-and-slow-wave.	**Slow-fused transient:** rappresenta un grafoelemento normale. Si intende una variante aguzza del normale ritmo dominante posteriore che precede un'onda lenta posteriore del giovane, dando la falsa impressione di un complesso punta-onda-lenta.

**Slow wave:** Wave with duration longer than alpha waves, i.e. over 1/8 s (>125 ms).	**Onda lenta:** un'onda con durata più lunga delle onde alfa, cioè oltre 1/8 s (>125 ms).

**Slow wave sleep:** Non-REM sleep stage N3. Synonym: deep sleep. (See deep sleep, REM sleep).	**Sonno ad onde lente:** stadio N3 del sonno non REM. Sinonimo: sonno profondo. (Vedi sonno profondo, sonno REM).

**Small sharp spikes (SSS)**: A normal variant. Small sharp spikes of very short duration (<50 ms) and low amplitude (<50 µV), often followed by a small theta wave, occurring in the temporal regions during drowsiness and light sleep. Synonym: benign epileptiform transients of sleep (use of term discouraged).	**Small sharp spikes (SSS):** sono una variante normale. Punte di piccola ampiezza di durata molto breve (<50 ms) e di bassa ampiezza (<50 µV), spesso seguite da una piccola onda theta, che si registrano nelle regioni temporali durante la sonnolenza e il sonno leggero. Sinonimo: transienti epilettiformi benigni del sonno (l'uso di questo termine è sconsigliato).

**Somatosensory evoked potential (SEP)**: Evoked potential in response to somatosensory stimulus, usually electrical stimulation of a sensory or mixed nerve (see evoked potential).	**Potenziale evocato somatosensoriale (PES, somatosensory evoked potential):** potenziale evocato in risposta a uno stimolo somatosensoriale, di solito la stimolazione elettrica di un nervo sensitivo puro o misto (vedi potenziale evocato).

**Special electrode:** Any electrode other than standard ten-twenty system scalp electrodes (for example surface sphenoidal or anterior “cheek” electrode, and closely spaced electrodes; see ten-ten system).	**Elettrodo speciale:** qualsiasi elettrodo diverso dagli elettrodi standard posizionati secondo il sistema 10-20 (per esempio un elettrodo sfenoidale o un elettrodo zigomatico; oppure elettrodi posizionati a minor distanza, vedi sistema 10-10).

**Sphenoidal electrode:** Strictly needle or wire electrode inserted through the soft tissues of the face below the zygomatic arch so that its tip lies near the base of the skull in the region of the foramen ovale, designed to record from the medial temporal lobe structures. (See basal electrode).	**Elettrodo sfenoidale:** elettrodo ad ago od a filo inserito attraverso i tessuti molli del viso sotto l'arco zigomatico in modo che la sua punta si trovi vicino alla base del cranio, nella regione del forame ovale. Progettato per registrare dalle strutture del lobo temporale mediale. (Vedi anche elettrodo basale).

**Spike:** A transient, clearly distinguished from background activity, with pointed peak at a conventional time scale and duration from 20 to less than 70 ms. Amplitude varies but typically >50 μV. Main component is generally negative relative to other areas. Comments: (1) term should be restricted to epileptiform discharges. EEG spikes should be differentiated from sharp waves, i.e. transients having similar characteristics but longer durations. However, it should be kept in mind that this distinction is largely arbitrary and primarily serves descriptive purposes. (2) EEG spikes should be clearly distinguished from the brief unit spikes recorded from single cells with microelectrode techniques. (See sharp wave).	**Punta:** definisce un grafoelemento, chiaramente distinto dall'attività di fondo, con picco appuntito (in una scala temporale convenzionale) e di durata da 20 a meno di 70 ms. L'ampiezza varia ma tipicamente è >50 μV. La componente principale è generalmente negativa rispetto alle altre aree. Commenti: (1) il termine dovrebbe essere limitato alle scariche epilettiformi. Le punte EEG dovrebbero essere differenziate dalle onde definite puntute, cioè dai transienti che hanno caratteristiche simili ma durate più lunghe. Tuttavia, questa distinzione è in gran parte arbitraria e serve principalmente a scopi descrittivi; (2) le punte EEG dovrebbero essere chiaramente distinte dalle punte registrate dalle singole cellule con tecniche microelettrodiche. (Vedi onda puntuta).

**Spike-and-slow-wave complex:** An epileptiform pattern consisting of a spike and an associated following slow wave, clearly distinguished from background activity. May be single or multiple.	**Complesso di punta-onda-lenta:** un grafoelemento epilettiforme che consiste in una punta e in un'onda lenta associate, chiaramente distinte dall'attività di fondo. Può essere un complesso singolo o multiplo.

**Spindle:** Group of rhythmic waves characterized by a progressively increasing, then gradually decreasing, amplitude (see sleep spindle).	**Fuso:** gruppo di onde ritmiche caratterizzate da un'ampiezza progressivamente crescente e poi gradualmente decrescente (vedi fuso del sonno).

**Spread:** Propagation of EEG waves from one region of the scalp and/or brain to another (see generalization, propagation).	**Diffusione:** propagazione delle onde EEG da una regione dello scalpo e/o del cervello a un'altra (vedi generalizzazione, propagazione).

**Standard electrode**: Conventional scalp electrode (see disk electrode, needle electrode, pad electrode, special electrode).	**Elettrodo standard:** elettrodo convenzionale per lo scalpo (vedi elettrodo a coppetta, elettrodo ad ago, elettrodo a tampone, elettrodo speciale).

**Standard electrode placement:** Scalp electrode location(s) determined by the ten-twenty system (see ten-twenty system).	**Posizionamento standard dell'elettrodo:** posizione dell'elettrodo sullo scalpo determinata dal sistema dei 10-20 (vedi sistema 10-20).

**Status epilepticus, EEG:** The occurrence of virtually continuous or repetitive epileptiform seizure pattern in an EEG. Term should be distinguished from clinical status epilepticus, although they may co-exist. (See seizure pattern). Synonym: electrographic status epilepticus.	**Stato epilettico, EEG:** Il verificarsi di attività epilettiformi praticamente continue o ripetitive in un EEG. Il termine dovrebbe essere distinto dallo stato epilettico clinico, anche se possono coesistere. (Vedi pattern di crisi). Sinonimo: stato epilettico elettrografico.

**Stereotactic (stereotaxic) electroencephalogram (SEEG)**: Intracerebral EEG recordings using electrodes implanted stereotactically, thus permitting the calculation of electrode coordinates that can be projected on a stereotactic brain atlas or magnetic resonance images to create three-dimensional pictures. Note the abbreviation SDEEG is also used for stereotactic depth electroencephalogram. Synonym: stereoelectroencephalography.	**Elettroencefalografia stereotassica (SEEG):** registrazioni EEG intracerebrali con elettrodi impiantati in modo stereotassico, permettendo così il calcolo delle coordinate degli elettrodi che possono essere proiettate su un atlante cerebrale stereotassico o su immagini di risonanza magnetica per creare immagini tridimensionali. Si noti che l'abbreviazione SEEG è usata anche per l'elettroencefalogramma stereotassico con elettrodi di profondità (depth). Sinonimo: stereoelettroencefalogramma.

**Stereotactic (stereotaxic) electroencephalography (SEEG)**: Technique of recording stereotactic (stereotaxic) electroencephalograms. Synonym: stereoelectroencephalogram.	**Elettroencefalogramma stereotassico (SEEG):** tecnica di registrazione stereotassica del segnale EEG (stereotassico). Sinonimo: stereoelettroencefalogramma.

**Sternospinal reference:** A non-cephalic reference achieved by interconnecting two electrodes placed over the right sternoclavicular junction and the spine of the seventh cervical vertebra, respectively, and balancing the voltage between them by means of a potentiometer to reduce ECG artifact.	**Referenza sterno-spinale:** referenza non-cefalica ottenuta collegando due elettrodi posti rispettivamente sulla giunzione sternoclavicolare destra e sulla colonna vertebrale a livello della settima vertebra cervicale, e bilanciando il voltaggio tra loro per mezzo di un potenziometro per ridurre l'artefatto ECG.

**Stimulus-induced rhythmic, periodic or ictal discharges (SIRPIDs):** Sharp transients that are rhythmic, periodic or ictal appearing discharges in comatose patients that are consistently induced by alerting stimuli including: auditory and other sensory stimulation, such as noxious (airway suctioning) and other patient-care activities. SIRPIDs may be regional or lateralized, bilateral or generalized, and of variable duration. Their pathophysiology and clinical significance is uncertain, but on occasions may be associated with clinical seizures.	**Scariche ritmiche, periodiche o ictali indotte da stimolo (SIRPIDs, stimulus-induced rhythmic, periodic or ictal discharges):** scariche ritmiche, periodiche o ictali che si manifestano nei pazienti in coma e che sono costantemente indotte da stimoli di allerta tra cui: stimoli uditivi e altri stimoli sensoriali, come quelli nocicettivi (aspirazione delle vie aeree) ed altre attività di cura del paziente. Le SIRPID possono essere regionali o lateralizzate, bilaterali o generalizzate e di durata variabile. La loro fisiopatologia e il loro significato clinico sono incerti, ma in alcune occasioni possono essere associati a crisi cliniche.

**Subclinical rhythmic discharges of adults (SREDA):** This paroxysmal pattern is usually seen in the adult age group (typically over 50 years) and consists of a mixture of frequencies, often predominantly in the theta range, lasting 40–80 s. It may resemble a seizure discharge but is not accompanied by any clinical signs or symptoms. The significance of this pattern is uncertain, but it should be distinguished from an epileptiform seizure pattern.	**Scariche ritmiche subcliniche degli adulti (SREDA, subclinical rhythmic electrographic discharges of adults):** questo pattern parossistico è di solito osservato in soggetti di età adulta (tipicamente oltre i 50 anni) e consiste in un mix di frequenze, spesso prevalentemente nella gamma theta, della durata di 40-80 s. Può assomigliare a una scarica critica ma non è accompagnata da alcun segno o sintomo clinico. Il significato di questo pattern è incerto, ma dovrebbe essere distinto da un pattern epilettiforme critico.

**Subdural electrode:** Electrode inserted under the dural covering of the cerebrum for recording of electrocorticogram as a pre-surgical evaluation of medically intractable partial epilepsy, usually in the form of electrode strips. Synonym: epicortical electrode (use of term discouraged).	**Elettrodo subdurale:** elettrodo inserito sotto la dura madre per la registrazione dell'elettrocorticogramma come valutazione prechirurgica dell'epilessia focale farmacoresistente, di solito sotto forma di strisce di elettrodi. Sinonimo: elettrodo epicorticale (l'uso del termine è sconsigliato).

**Suppression:** Entirety of an EEG record showing activity below 10 µV (reference derivation). (See burst suppression pattern).	**Soppressione:** registrazione EEG che mostra una attività al di sotto di 10 µV (in montaggio referenziale). (Vedi burst-suppression).

**Symmetry**: Approximately equal amplitude, frequency and form of EEG activities over homologous areas on opposite sides of the head.	**Simmetria:** ampiezza, frequenza e forma approssimativamente identiche delle attività EEG su aree omologhe sui lati opposti dello scalpo.

**Synchrony:** The simultaneous occurrence of EEG waves over distinct regions on the same or opposite sides of the head with the same speed and phase. Comment: term simultaneous only implies a lack of possible delay that is measurable with standard computer display. Certain electrodes are so close (for example Fp1-Fp2 and 01–02 respectively) that volume conduction can affect the signal on the other side, making these electrodes unsuitable for assessing synchrony). (See volume conduction).	**Sincronia:** l'occorrenza simultanea di onde EEG su regioni distinte su uno stesso lato o su lati opposti dello scalpo con la stessa frequenza e fase. Commento: il termine simultaneo implica solo la mancanza di un possibile ritardo misurabile con la visualizzazione standard del computer. Alcuni elettrodi sono così vicini (per esempio Fp1-Fp2 e O1-O2 rispettivamente) che il fenomeno del 'volume condotto' può influenzare il segnale sull'altro lato, rendendo questi elettrodi inadatti a valutare la sincronia). (Vedi il significato di 'volume condotto').

**Temporal intermittent rhythmic delta activity (TIRDA)**: An EEG pattern characterized by short trains of intermittent and rhythmic delta activity (1–3.5 Hz), often with sawtooth morphology, recorded predominantly over the anterior temporal regions. TIRDA usually occurs during drowsiness and light sleep and may either be unilateral or bilateral, occurring independently. It is associated with temporal lobe epilepsy, and when unilateral is virtually indicative of ipsilateral pathology.	**Attività delta ritmica intermittente temporale (TIRDA, temporal intermittent rhythmic delta activity):** un quadro EEG caratterizzato da brevi treni di attività delta intermittente e ritmica (1-3,5 Hz), spesso con morfologia a dente di sega, registrata prevalentemente nelle regioni temporali anteriori. Il pattern TIRDA si verifica di solito durante la sonnolenza ed il sonno leggero e può essere unilaterale o bilaterale, anche in modo indipendente. È associato all'epilessia del lobo temporale, e quando è unilaterale è praticamente indicativo di una patologia omolaterale.

**Temporal slow activity of the elderly:** Pattern of uncertain significance but usually not considered abnormal. Unilateral (most often on the left side) or bilateral, short runs of theta or delta activity intermixed with the background activity over the temporal region(s), in subjects >50 years of age, without clinical abnormalities. Often accentuates during drowsiness and hyperventilation.	**Attività lenta temporale degli anziani:** quadro di significato incerto ma di solito non considerato anormale. Unilaterale (più spesso sul lato sinistro) o bilaterale, brevi sequenze di attività theta o delta mescolate con l'attività di fondo sulla/e regione/i temporale/i, in soggetti >50 anni di età, non associate a manifestazioni cliniche. Spesso si accentua durante la sonnolenza e l'iperventilazione.

**Ten-ten (10–10) system:** System of standardized scalp electrode placement. According to this system, additional scalp electrodes are placed at half distance between the standard electrodes of the ten-twenty system, i.e. 10 percentile increments of the reference curve (see ten-twenty system, closely spaced electrodes). Comment: use of additional supplementary scalp electrodes is indicated for instance during epilepsy monitoring, theoretically in order localize epileptiform discharges more precisely (for example surface sphenoidal or anterior “cheek” electrode). (See special electrode).	**Sistema dieci-dieci (10-10):** sistema di posizionamento standardizzato degli elettrodi di scalpo. Secondo questo sistema, gli elettrodi supplementari sono posizionati a metà della distanza tra gli elettrodi standard del sistema 10-20, vale a dire a distanze reciproche del 10% rispetto alla distanza nasion-inion (vedi sistema 10-20). Commento: l'uso di elettrodi supplementari di scalpo è indicato per esempio durante il monitoraggio dell'epilessia, teoricamente per localizzare più precisamente le scariche epilettiformi (per esempio elettrodi sfenoidali o zigomatico anteriore). (Vedi elettrodo speciale).

**Ten-twenty (10–20) system:** System of standardized scalp electrode placement recommended by the International Federation of Clinical Neurophysiology. According to this system, the placement of electrodes is determined by measuring the head from 4 external landmarks and taking 10 or 20 percentiles of these measurements. Comment: the use of additional supplementary scalp electrodes, such as electrodes in the inferior temporal chain of electrodes, is indicated in various circumstances (for example focal epilepsy investigation).	**Sistema dieci-venti (10-20):** sistema di posizionamento standardizzato degli elettrodi di scalpo raccomandato dalla Federazione Internazionale di Neurofisiologia Clinica. Secondo questo sistema, il posizionamento degli elettrodi è determinato misurando la testa da 4 punti di riferimento esterni e utilizzando il 10 o 20% di queste misure. Commento: l'uso di elettrodi supplementari di scalpo, come gli elettrodi della catena temporale inferiore, è indicato in varie circostanze (per esempio nell'indagine dell'epilessia focale).

**Theta band:** Frequency band from 4−<8 Hz. Greek letter: θ.	**Banda theta:** banda di frequenza da 4-<8 Hz. Lettera greca: θ.

**Theta rhythm:** Rhythm with a frequency of 4−<8 Hz.	**Ritmo theta**: ritmo con una frequenza di 4−<8 Hz.

**Theta wave:** Wave with duration of 1/4 to over 1/8 s (125–250 ms).	**Onda theta:** onda con durata da 1/4 a più di 1/8 s (125-250 ms).

**Three Hz or three per second spike-and-slow-wave complex**: Characteristic ictal paroxysmal pattern consisting of a regular sequence of spike-and-slow-wave complexes which: (1) repeat at around 3–3.5 c/s (measured during the first few seconds of the paroxysm), (2) are bilaterally synchronous in their onset and termination (i.e. “generalized”), and usually of maximal amplitude over the frontal areas, and (3) remain approximately synchronous and symmetrical on the two sides of the head throughout the paroxysmal burst. Amplitude varies but can reach values of 1000 µV (1 mV). Characteristic seizure pattern associated with Childhood Absence Epilepsy.	**Complessi di punta-onda-lenta a 3 Hz:** quadro parossistico ictale caratteristico che consiste in una sequenza regolare di complessi di punta-onda-lenta che: (1) si ripetono a circa 3-3,5 Hz (misurati durante i primi secondi del parossismo); (2) sono bilateralmente sincroni nella loro insorgenza e fine (cioè “generalizzati”), e solitamente di ampiezza massima sulle aree frontali, e (3) rimangono approssimativamente sincroni e simmetrici sui due lati dello scalpo per tutto il burst. L'ampiezza varia ma può raggiungere valori di 1000 µV (1 mV). Pattern critico caratteristico associato all'epilessia con assenza dell'infanzia.

**Time constant (TC):** Historically the product of the values of the resistance (in mega-ohms, MΩ) and the capacitance (in microfarads, μF) which make up the time constant control of an EEG channel. This product represents the time required for the trace to fall to 37% of the deflection initially produced when a DC voltage difference is applied to the input terminals of the amplifier. Expressed in seconds (s). Comment: for a simple Resistance-Capacitance coupling network, the TC is related to the percent reduction in sensitivity of the channel at a given stated low frequency by the equation TC =1/2πf, where f is the frequency at which a 30% (3 dB) attenuation occurs. For instance, for a TC of 0.3 s, an attenuation of 30% (3 dB) occurs at 0.5 Hz. Thus, either the time constant or the percent attenuation at a given stated low frequency can be used to designate the same position of the low frequency filter of the EEG channel (see low frequency filter). In the digital era this process is internally governed by the software of the system.	**Costante di tempo (TC, time constant):** storicamente il prodotto dei valori della resistenza (in mega-ohm, MΩ) e della capacità (in microfarad, μF) che determinano la costante di tempo di un canale EEG. Questo prodotto rappresenta il tempo necessario affinché la traccia scenda al 37% della deflessione prodotta inizialmente quando una differenza di tensione continua viene applicata all'amplificatore. Espresso in secondi (s). Commento: per una semplice rete di accoppiamento resistenza-capacità, la TC è correlata alla riduzione percentuale della sensibilità del canale ad una data bassa frequenza dichiarata dall'equazione TC =1/2πf, dove f è la frequenza alla quale si verifica un'attenuazione del 30% (3 dB). Per esempio, per un TC di 0,3 s, un'attenuazione del 30% (3 dB) si verifica a 0,5 Hz. Così, la costante di tempo o l'attenuazione percentuale a una determinata bassa frequenza può essere utilizzata per designare il valore del filtro a bassa frequenza del canale EEG (vedi filtro a bassa frequenza, filtro passa alto). Nei sistemi digitali questo processo è regolato internamente dal software del sistema.

**Topography:** Spatial distribution of EEG features (rhythm, voltage fields, spectra, evoked potential etc.) over the scalp or cerebral cortex. (See map voltage).	**Topografia:** distribuzione spaziale del segnale EEG (ritmo, mappa di voltaggio, spettri, potenziale evocato ecc.) sullo scalpo o sulla corteccia cerebrale. (Vedi mappa di voltaggio).

**Tracé alternant:** Neonatal EEG pattern of quiet (non-REM) sleep seen in term and near term infants from 34 to 36 weeks post menstrual age onwards, which may persist up to 4 weeks after birth. It is characterized by high amplitude bursts of predominantly slow waves (50–150 µV) alternating with periods of relatively low amplitude activity mixed activities (25–50 µV). (See quiet sleep).	**Tracciato alternante**: pattern EEG neonatale di sonno tranquillo (NREM) osservato nei neonati a termine e quasi a termine dalla 34 alla 36 settimana gestazionale in poi, che può persistere fino a 4 settimane dopo la nascita. È caratterizzato da burst con predominanza di onde lente di ampio voltaggio (50-150 µV) alternate a periodi di attività con frequenze miste di ampiezza relativamente bassa (25-50 µV). Sinonimo: tracé alternant. (Vedi sonno quieto).

**Tracé continu:** Continuous background activity, consisting of theta and/or delta frequencies (>25 µV), replacing a previously markedly intermittent record during evolution of EEG in preterm infants. Present in active sleep (see active sleep).	**Tracciato continuo**: attività di fondo continua, costituita da attività con frequenze theta e/o delta (>25 µV), che durante la maturazione dell'EEG nei neonati pretermine sostituisce il precedente “tracciato discontinuo”. Presente anche nel sonno attivo. Sinonimo: tracé continu. (Vedi sonno attivo, sonno quieto e tracciato discontinuo)

**Tracé discontinu:** Normal discontinuous pattern in preterm infants which is characterized by bursts of high amplitude (up to 300 µV) mixed frequency activity regularly interrupted by low amplitude interburst activity (<25 µV). The pattern can still be called discontinuous if there is a modest amount of EEG activity in a single channel or a single transient in multiple channels. The interburst interval depends on the post menstrual age (PMA). It is seen in wakefulness, active and quiet sleep until 30 weeks PMA, then only in quiet sleep, and is rarely seen in infants of 38 weeks PMA. (See active and quiet sleep).	**Tracciato discontinuo**: pattern fisiologico nei neonati pretermine, caratterizzato da burst di attività con frequenze miste (N.d.T.: lente e rapide), di alto voltaggio (fino a 300 µV) interrotte regolarmente da periodi di attività di bassa ampiezza (<25 µV), detti “inter-burst”. Il pattern può ancora essere chiamato discontinuo anche in presenza di una modesta quantità di attività in un singolo canale o un singolo transiente in più canali. La durata dei periodi inter-burst dipende dall'età gestazionale (EG). Si vede in veglia, in sonno attivo e sonno quieto fino a 30 settimane di EG, poi solo in sonno quieto (N.d.T.: in questo caso caratterizzando il “sonno quieto alternante”), ed è raramente visto in neonati a 38 settimane di EG. Sinonimi: tracé discontinu. (Vedi sonno attivo e sonno quieto).

**Tracing:** Synonyms: record, recording.	**Tracciato:** sinonimi: registrazione.

**Transient, EEG**: Any isolated wave or complex, distinguished from background activity.	**Transiente EEG:** qualsiasi onda o complesso isolato, distinto dall'attività di fondo.

**Transverse bipolar montage:** A montage consisting of contiguous channels of electrode pairs along transverse/coronal (i.e. side-to-side) arrays (for example F7-F3, F3-Fz, Fz-F4, F4-F8, etc.). Synonym: coronal bipolar montage.	**Montaggio bipolare trasversale:** un montaggio costituito da canali contigui di coppie di elettrodi lungo le linee trasversali/coronali (cioè da un lato all'altro dello scalpo) (per esempio F7-F3, F3-Fz, Fz-F4, F4-F8, ecc.). Sinonimo: montaggio bipolare coronale.

**Triangular bipolar montage:** A historic montage consisting of derivations from pairs of electrodes in a group of 3 electrodes arranged in a triangular pattern. Use of this montage is discouraged, because false lateralization may occur.	**Montaggio bipolare triangolare:** un montaggio storico che consiste in derivazioni da coppie di elettrodi in un gruppo di 3 elettrodi disposti a triangolo. L'uso di questo montaggio è sconsigliato, perché può favorire una falsa lateralizzazione.

**Triphasic wave (TW)**: High amplitude (over 70 µV) positive sharp transients (with respect to common average), which are preceded and followed by relatively low amplitude negative waves. The first negative wave generally has lower amplitude than the negative afterwave, and has a steeper slope, which on occasions may be sharp. Usually bilateral with an antero-posterior or a postero-anterior lag, and repetition rate of about 1−<2 c/s. TWs occur typically in runs, and can either regress or increase with arousal or noxious stimuli. Often there is little ongoing or low amplitude (<40 µV) slow background activity between TWs. They are seen in a range of conditions, often present concurrently, associated with metabolic encephalopathy. The patient is usually comatose, and TW may decrease with sleep and after intravenous benzodiazepines. Synonym: continuous 2/s GPDs (with triphasic morphology).	**Onda trifasica (TW, triphasic wave):** grafoelementi positivi di ampiezza elevata (oltre 70 µV, in riferimento alla media comune), che sono preceduti e seguiti da onde negative di ampiezza relativamente bassa. La prima onda negativa ha generalmente un'ampiezza inferiore rispetto all'onda negativa successiva, e ha una pendenza più ripida, che in alcune occasioni può essere aguzza-puntuta. Solitamente bilaterale con un ritardo antero-posteriore o postero-anteriore, e frequenza di ripetizione di circa 1-2 Hz. le onde TW si presentano tipicamente in serie, e possono regredire o aumentare con l'arousal o con stimoli nocicettivi. Spesso c'è scarsa attività di fondo lenta o di bassa ampiezza (<40 µV) tra i complessi TW. Sono osservati in una serie di condizioni, spesso presenti contemporaneamente, associati a encefalopatia metabolica. Il paziente è solitamente in stato comatoso e i complessi TW possono diminuire durante il sonno e dopo somministrazione di benzodiazepine per via endovenosa. Sinonimo: GPD continui 2/s (con morfologia trifasica).

**Unilateral:** Confined to one side of the head (or body). Comments: (1) unilateral EEG activities may be focal, regional or lateralized to one hemisphere. (2) They are said to be lateralized to the right or left side of the head. (See lateralized).	**Unilaterale:** confinato a un lato della testa (o del corpo). Commenti: (1) le attività EEG unilaterali possono essere focali, regionali o lateralizzate a un emisfero. (2) Si dice che siano lateralizzate al lato destro o sinistro dello scalpo. (Vedi lateralizzato).

**Vertex sharp transient or vertex sharp wave (V wave)**: A normal graphoelement. Sharply contoured wave with duration <0.5 s, maximal at the vertex, negative relative to other areas, apparently occurring spontaneously during light sleep or in response to a sensory stimulus (usually auditory). Vertex sharp waves may be single or repetitive. Amplitude varies but rarely exceeds 250 µV. (See light sleep, K complex).	**Punte al vertice (onda V):** un grafoelemento fisiologico. Onda dal contorno appuntito con durata <0,5 s, massima al vertice, negativa rispetto ad altre aree, che apparentemente si verifica spontaneamente durante il sonno leggero od in risposta a uno stimolo sensoriale (di solito uditivo). Le punte al vertice possono essere singole o ripetitive. L'ampiezza varia ma raramente supera i 250 µV. (Vedi sonno leggero, complesso K).

**Visual evoked potential:** Cortical evoked potential generated in response to visual stimulus, either unstructured diffuse flashes or patterned (for example pattern-reversal stimulus). (See evoked potential).	**Potenziale evocato visivo:** potenziale evocato corticale generato in risposta a stimoli visivi, sia flash che pattern (per esempio stimolo pattern-reversal). (Vedi potenziale evocato).

**Voltage:** The difference in electric potential between two points (units: volts). (See amplitude).	**Voltaggio:** la differenza di potenziale elettrico tra due punti (unità: volt). (Vedi ampiezza).

**Volume conduction:** The passive process by which electrical activity, originating from a generator, spreads through a conductive medium to be detected quite widely by distant (i.e. far-field) recording electrodes, without being mediated by neural activity (for example, see Brainstem Auditory Evoked Potential). (See propagation).	**Volume condotto:** il processo passivo attraverso il quale l'attività elettrica, originata da un generatore, si diffonde attraverso un mezzo conduttivo per essere rilevata da elettrodi di registrazione distanti (cioè far-field), dal generatore neurale (per esempio, vedi potenziale evocato uditivo del tronco encefalico). (Vedi propagazione).

**Wave:** Any change of the potential difference between pairs of electrodes in EEG recording, which may arise in the brain (an EEG wave) or outside of it (i.e. extracerebral potential).	**Onda:** qualsiasi cambiamento della differenza di potenziale tra coppie di elettrodi nella registrazione EEG, che può verificarsi nel cervello (un'onda EEG) o al di fuori di esso (cioè un potenziale extracerebrale).

**Waveform (wave form)**: The shape or morphology of an EEG wave (see morphology).	**Forma d'onda (wave form):** la forma o morfologia di un'onda EEG (vedi morfologia).

**Wicket spikes or wicket waves:** Spike-like monophasic surface negative single waves or trains of waves occurring over the temporal regions, typically unilateral, during drowsiness that have an arcuate or mu-like appearance. These are mainly seen in older individuals and represent a benign physiological variant, but may also be seen in patients with a clinical diagnosis of epilepsy.	**Wicket spikes o wicket waves:** onde singole monofasiche a polarità negativa o treni di onde, tipicamente unilaterali, che compaiono sulle regioni temporali durante la sonnolenza e che hanno un aspetto arcuato o mu. Si osservano principalmente in individui anziani e rappresentano una variante fisiologica benigna, ma possono anche essere registrate in pazienti con una diagnosi clinica di epilessia.

**Writer:** Historically a system for direct write-out of the output of an EEG channel. Most writers used ink delivered by a pen, but in certain instruments the ink was sprayed in a jet stream, and in others the pen writer used carbon paper. Used infrequently since the advent of digital EEG, where laser printers are used to write-out EEGs.	**Pennino EEG:** storicamente un sistema per la scrittura diretta dell'uscita di un canale EEG su supporto cartaceo. Il sistema scrivente era costituito di solito da pennini od ugelli attraverso i quali fuoriusciva l'inchiostro, ma in alcuni strumenti veniva usato come supporto la carta carbone. Viene usato sempre più raramente dopo l'avvento dell'EEG digitale, dove le stampanti laser sono usate per stampare gli EEG.

**Appendix A. EEG report**	**Appendice A. referto EEG**
The purpose of the EEG report is to document clinically relevant items, and this determines its format: patient demographics, reason for EEG, specification of techniques, description of pattern seen, and finally the clinical interpretation and conclusion within that clinical context.	Lo scopo del referto EEG è quello di documentare elementi clinicamente rilevanti, e questo determina il suo formato: dati demografici del paziente, motivo dell'EEG, dettagli sulle tecniche utilizzate, descrizione dei pattern visti, e infine l'interpretazione clinica e la conclusione in quel contesto clinico.

Standardizing the structure and content of the report is essential for quality assurance. A standardized report format helps communication between the patient’s primary physician and EEG departments, and lists all items that have clinical relevance. The importance of the report goes beyond communicating the results: it also points out the features that have to be assessed in clinical EEG recordings. Several EEG report-templates have been previously proposed (Noachtar et al., 1999, Noachtar et al., 1999, American Clinical Neurophysiology Society, 2006, Kaplan and Benbadis, 2013, Shibasaki et al., 2014, Tatum et al., 2016). More recently computer-based reporting systems have been developed, where the reviewer is guided through the structured reporting system by choosing the features from a predefined list. SCORE (Standardized Computer-based Organized reporting of EEG) has been developed under the auspices of ILAE and IFCN (Beniczky et al., 2013). We suggest the following template for reporting EEG in clinical practice:	La standardizzazione della struttura e del contenuto del referto è essenziale per la garanzia della qualità. Un formato di referto standardizzato aiuta la comunicazione tra i clinici, ed elenca tutte le voci che hanno rilevanza clinica. L'importanza del referto va oltre la comunicazione dei risultati: esso indica anche le caratteristiche che devono essere valutate nelle registrazioni EEG cliniche. Diversi modelli di referti EEG sono stati proposti in precedenza (Noachtar et al., 1999, Noachtar et al., 1999, American Clinical Neurophysiology Society, 2006, Kaplan and Benbadis, 2013, Shibasaki et al., 2014, Tatum et al., 2016). Più recentemente sono stati sviluppati sistemi di reporting basati sul computer, dove il revisore è guidato attraverso il sistema di reporting strutturato scegliendo le caratteristiche da una lista predefinita. SCORE (Standardized Computer-based Organized reporting of EEG) è stato sviluppato sotto gli auspici di ILAE e IFCN (Beniczky et al., 2013). Suggeriamo il seguente modello per la refertazione EEG nella pratica clinica:

**Dati demografici e anamnesi del paziente**

**Table:** 

**Dati del report**	**Informazioni essenziali/spiegazione**
ID	Identificativo come richiesto dalle linee guida locali
Età e data di nascita	Per i neonati, anche l’età gestazionale
Medico inviante	
Medico di famiglia	
Anamnesi	
Diagnosi	Inclusa la condizione neurologica
Neuroimmagini	
Precedenti EEG	
Terapia farmacologica	Con particolare attenzione ai farmaci attivi sul SNC
Informazioni relative all’eventuale deprivazione di sonno	
Ultima crisi	
Quesito clinico	

**Registrazione**

**Table:** 

**Dati del report**	**Informazioni essenziali/spiegazione**
Montaggio	Tipo di montaggio
	Specificare i canali poligrafici
Tipo di registrazione e durata	Standard in veglia/in sedazione/deprivazione di sonno/ambulatoriale/monitoraggio video-EEG a lungo termine
Procedure di attivazione/ modulazione	Chiusura occhi (obbligatoria per ogni età, attiva o passiva); iperventilazione; stimolazione luminosa intermittente; stimolazione sensoriale esterna; farmaci somministrati durante la registrazione
Livello di coscienza/vigilanza	Includere la capacità di collaborazione del paziente

**Descrizione**

**Table:** 

**Dati del report**	**Informazioni essenziali/spiegazione**
Attività di fondo	Simmetria, sincronia
Veglia	Ritmo dominante posteriore
	Altri ritmi
Sonno	Stadi del sonno raggiunti
Attività intercritica	Descrivere le seguenti caratteristiche per ogni tipo di pattern intercritico osservato:
	*morfologia* (specificare il tipo di anomalie epilettiformi, i rallentamenti anormali o pattern specifici – utilizzando la terminologia del glossario)
	*localizzazione*
	*caratteristiche tempo-correlate*: quanto spesso il pattern descritto si presenta durante la registrazione; se si presenta in una singola scarica oppure in burst o sequenze (in questo caso specificare durata e frequenza)
Episodi clinici/crisi	Semeiologia; EEG critico; classificazione delle crisi
Varianti normali/pattern di significato incerto	
Artefatti	Impatto sul valore diagnostico del tracciato
Effetto delle prove di attivazione/modulazione	Modificazioni del tracciato con le prove di attivazione e il sonno

**Interpretazione**

**Table:** 

**Dati del report**	**Informazioni essenziali/spiegazione**
Classificazione	Normale/non chiare anormalità/anormale
Sinopsi	Breve riassunto dei reperti essenziali
Significato diagnostico	Interpretazione dei reperti EEG nel contesto clinico
Commenti clinici	Risposte a specifici quesiti; suggerimenti per eventuali ulteriori accertamenti

**Acronimi**

**Table:** 

AEP, auditory evoked potential
BAEP, brainstem auditory evoked potential
BETS, benign epileptiform transient of sleep
BIPDs, bilateral independent periodic discharges
BIPLEDs, bilateral independent periodic lateralized epileptiform discharges
BSI, brain symmetry index
cEEG, continuous EEG
CFM, cerebral function monitoring
CNV, contingent negative variation
CMRR, common mode rejection ratio
CSWS, continuous spike and waves during sleep
EP, evoked potential
ERP, event-related potential
ESES, electrical status epilepticus during sleep
FIRDA, frontal intermittent rhythmic delta activity
GPDs, generalized periodic discharges
GPEDs, generalized periodic epileptiform discharges
GPFA, generalized paroxysmal fast activity
GRDA, generalized rhythmic delta activity
HFO, high frequency oscillations
LPC, late positive component
LPDs, lateralized periodic discharges
MMN, mismatch negativity
OIRDA, occipital intermittent rhythmic delta activity
PBR, posterior basic rhythm
PDR, posterior dominant rhythm
PDs, periodic discharges
PLEDs, periodic lateralized epileptiform discharges
POSTS, positive occipital sharp transient of sleep
PPR, photoparoxysmal response
PRSW, positive rolandic sharp waves
PS, photic stimulation
qEEG, quantitative EEG
REM, rapid eye movements
SEP, somatosensory evoked potential
SIRPIDs, stimulus-induced rhythmic, periodic or ictal discharges
SOREM, sleep onset REM
SREDA, subclinical rhythmic electrographic discharges of adults
SSS, small sharp spikes
TIRDA, temporal intermittent rhythmic delta activity

## Declaration of Competing Interest

The authors declare that they have no known competing financial interests or personal relationships that could have appeared to influence the work reported in this paper.
